# Modern arc-like water content in the source of 3.1-billion-year-old volcanic rocks

**DOI:** 10.1038/s41467-026-74653-1

**Published:** 2026-07-07

**Authors:** Eric D. Vandenburg, Oliver Nebel, R. Hugh Smithies, Peter A. Cawood, Laura A. Miller, Marc-Alban Millet, Fabio A. Capitanio

**Affiliations:** 1 Australian Critical Minerals Research Centre, Department of Earth Sciences, School of Physics, Chemistry and Earth Sciences, Adelaide University, Adelaide, SA Australia; 2 ARC Training Centre in Critical Resources for the Future, Perth, WA Australia; 3 School of Earth, Atmosphere and Environment, Monash University, Clayton, VIC Australia; 4 GEOMAR Helmholtz Centre for Ocean Research Kiel, Kiel, Germany; 5 Geological Survey of Western Australia, Department of Mines, Industry Regulation and Safety, Perth, WA Australia; 6 Timescales of Mineral Systems Group, Curtin Frontier Institute of Geoscience Solutions, School of Earth and Planetary Science, Curtin University, Bentley, WA Australia; 7 Research School of Earth Sciences, Australian National University, Canberra, ACT Australia; 8 School of Earth and Environmental Sciences, Cardiff University, Cardiff, UK

**Keywords:** Geochemistry, Petrology, Precambrian geology

## Abstract

Whether Archean arc-like volcanism reflects subduction remains debated. We present high-resolution geochemical data from a well-preserved 3.13-3.10 Ga arc-like volcanic succession in Australia’s Pilbara Craton, a rare Archean analog of modern arc volcanism retaining fluid-mobile element concentrations consistent with primary magmatic values. The sequence records three primitive lava series typical of modern arcs: tholeiitic, calc-alkaline, and the oldest stratigraphically extensive genuine boninites. Geochemical modelling shows this melt diversity requires at least two mantle sources with distinct depletion histories. The mantle H_2_O required for fluid-assisted melting to produce these lavas substantially exceeds primitive mantle, approaching the H_2_O-saturated solidus of modern mantle wedges. We infer hydrous melting was triggered by dripduction, the short-lived inclined foundering of hydrated lithosphere without laterally continuous plate boundaries, in an off-plateau setting. Dripduction locally recycled surface water and generated arc-like magmas without self-sustained plate tectonics, possibly promoting mantle-ocean-atmosphere volatile exchange during the Archean.

## Abstract (web summary)

Long before plate tectonics, the Earth was already building arc-like volcanoes and recycling seawater into its interior; 3.1-billion-year-old Australian rocks reveal “dripduction,” where hydrated crust drips into the mantle.

## Introduction

Recycling lithospheric material into the mantle is a crucial process that shapes the physico-chemical evolution of Earth and its habitability. Subduction zones are the primary sites for recycling during the Phanerozoic eon (0.5 Ga-present), contributing to the growth of new continental crust through arc magmatism and accretion^1,2^, mantle re-enrichment, and the regulation of habitability-critical processes, such as Earth’s water budget^3^ and the global nitrogen cycle^4,5^. However, the subduction record becomes increasingly obscure in the Archean (4.0–2.5 billion years ago; Ga) eon, especially before 2.8 Ga. Crucially, debates on the mechanisms that facilitated lithosphere-asthenosphere interactions and volatile recycling during Earth’s infancy rely on samples from cratons; areas of cratons older than 2.8 Ga comprise only seven percent of Earth’s present-day continents^6^. Most of the crust from that time is missing^7,8^; thus, new insights from rare but possibly representative crustal sections are critical to unravel the tectonic processes underpinning the global geochemical cycling of elements necessary to sustain life on early Earth.

The processes operating in the Archean that acted as triggers for continental crust formation remain controversial. Evidence from Archean locales suggest that the earliest continental crust was formed in non-plate tectonic environments akin to mafic-ultramafic plateaux^9–11^, alternative interpretations have suggested that subduction played a key role. These alternative interpretations primarily rely upon estimates of H_2_O content and oxygen fugacity from zircons in Archean granitoids (e.g., ref. ^12^), particularly of the tonalite-trondhjemite-granodiorite (TTG) series. Although they may occur in Archean subduction-like settings, TTGs are not diagnostic, given that they are melts of pre-existing mafic crust that have not interacted with mantle peridotite^10,13^ and only provide genuine evidence of some form of hydrous melting of mafic crust, which may not require subduction (e.g., refs. ^10,14,15^.). Rather, the detailed investigation of trace element systematics of mantle-derived lavas (e.g., ref. ^16^) using process-indicative proxies could be diagnostic for early Earth-style subduction.

Benioff-style subduction is mechanically challenging if not impossible in the Archean due to the rheological differences imparted by a hotter Earth^17^. Whilst this does not prevent a direct comparison of Archean geochemical data with modern analogs, it makes investigations and identifications of early proto-subduction challenging, as reasonable geodynamic constraints need to be satisfied to explain certain geochemical patterns. Recent numerical modeling experiments demonstrate that mantle tractions could cause the inclined foundering of ductile, hydrated lithosphere in an early Earth with a sluggish lid. This process of proto-subduction, called dripduction^18–21^, resembles intermittent, localized subduction without continuous plate margins, manifested by a down-going slab prone to frequent breakoff and short-lived bursts of hydrous magma production. Only seven pre-2.8 Ga sites with putative proto-subduction origins have been identified in the volcanic record^21^, making up an insignificant proportion of the Archean crust. Crucially, all but one of these sites are of off-proto-cratonic plateau affinity, characterized by juvenile crust that developed without a pre-existing felsic basement. This is important as these sites do not contain abundant earlier TTG basements, which are often used to study subduction, require lower crustal delamination, and occur in slightly thicker crustal sections (>30 km)^9,10^. While the proto-cratonic plateau nuclei, which include TTG and form most of the preserved Archean crust, do not appear to record substantial dripduction processes, it is therefore possible that these processes occurred outside these plateaux, in the comparatively undifferentiated, thin crust now largely missing from the geological record. In rare cases, off-plateau juvenile crust is incorporated during craton assembly^22,23^, helping to preserve isolated fragments within cratons.

Studying primitive melts in modern subduction environments presents a vital opportunity to uncover crucial information on the mantle wedge, temporal changes in slab inputs, and melting depths. This information forms the basis of petrological models for identifying such environments in deep time. Of particular importance are boninites—high Si, high-Mg, and low-Ti lavas—typically produced during the early stages of subduction through the fluid-fluxed melting of refractory harzburgitic mantle^24,25^. Similar lavas have been found in Archean localities, with samples as old as 3.8 Ga^24^, forming the basis of arguments for subduction in the early Archean^16,26^. However, most “boninitic” samples either do not conform to established classification schemes^25^, or are cumulates that cannot be used to infer parental magma composition. True early Archean boninites exist only as isolated samples in volcanic packages that deviate from the magmatic evolutionary trends of modern boninite suites (Supplementary Fig. 1). Moreover, most locations older than 3 Ga have experienced moderate to high degrees of metamorphism, which often overprints the primary fluid-mobile elemental signatures crucial to fingerprinting subduction processes. Therefore, identifying relatively pristine off-plateau locales is fundamental in resolving the nature of Archean lithospheric recycling.

**Fig. 1:**
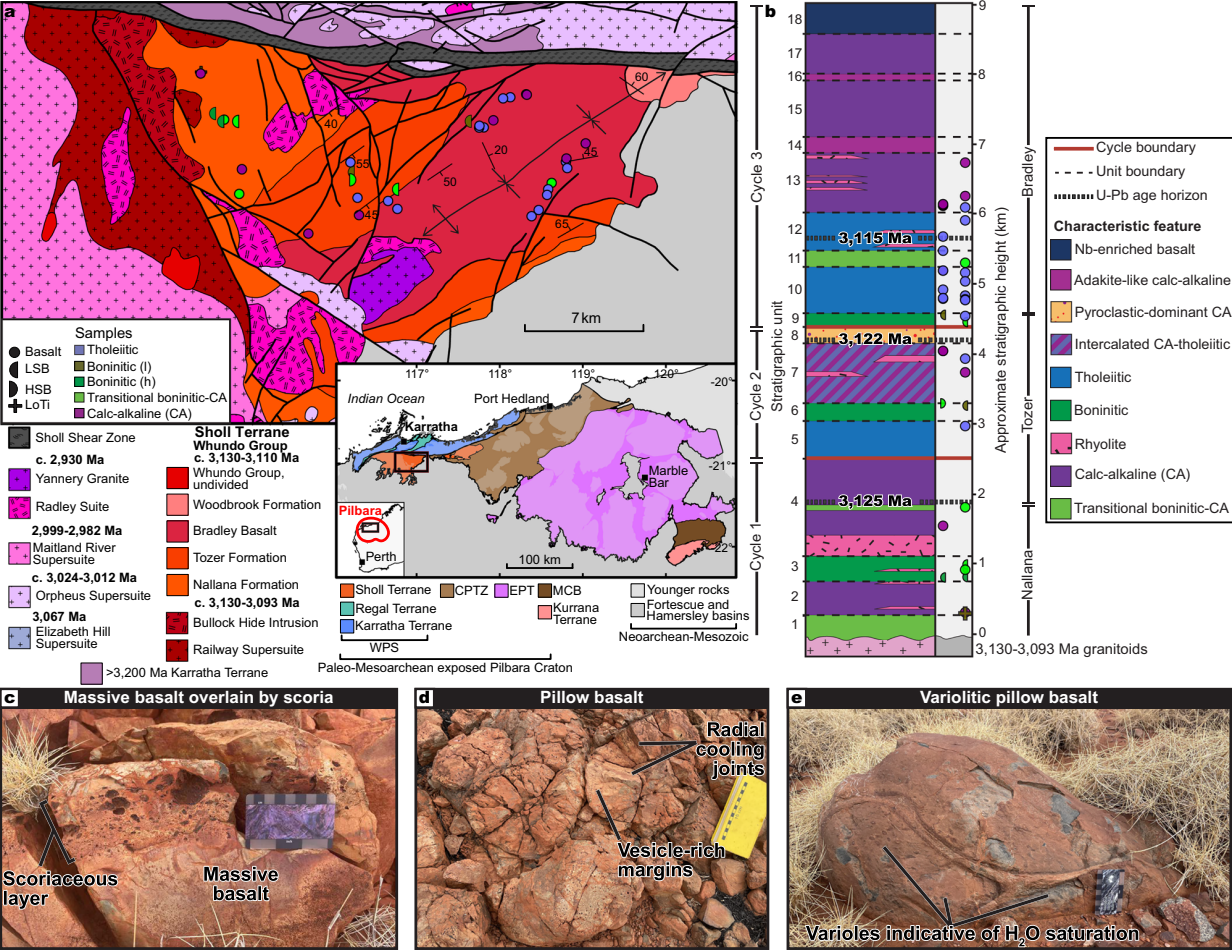
Geological setting and stratigraphy of the Whundo Group, and images demonstrating exceptional preservation of primary textures in Whundo primitive lavas. The Whundo Group was only subjected to low degrees of metamorphism and deformation, thereby preserving macroscopic volcanic textures on a scale rarely observed in Archean rocks. **a** Geological map of the central Sholl Terrane, illustrating the distribution of Whundo Group volcanic rocks. The different symbols show the location of samples discussed in this study as used in the following figures. The inset shows the lithotectonic divisions of the exposed portion of the Pilbara Craton. **b** Schematic chemostratigraphic column of the Whundo Group, showing relative proportions of units and position of specific samples. **c** Massive basalt overlain by scoria; note recessive weathering of larger lapilli-sized fragments. **d** Pillow basalts retain original depositional textures, including radial cooling joints and vesicle-rich margins. **e** Pillow basalt with variolitic texture, indicating H_2_O saturation (i.e., ref. ^98^). U-Pb ages are from ref. ^99^, and maps use data from ref. ^100^. Abbreviations: CPTZ—Central Pilbara Tectonic Zone, EPT—East Pilbara Terrane, HSB—High-Si boninite, LOTI—low-Ti basalt, LSB—Low-Si boninite, MCB—Mosquito Creek Basin, and WPS—Western Pilbara Superterrane.

Here, we provide a detailed account of the origin and formation processes of arc-like primitive melts from the 3.13 to 3.10 Ga Whundo Group of the Pilbara Craton (Fig. 1 and Supplementary Text), including Earth’s oldest known example of widespread and volumetrically significant boninite volcanism. The Pilbara Craton is considered the least altered and most pristine region of pre-2.8 Ga crust on Earth. The Whundo Group is of off-plateau affinity and exists in a region of thin, relatively undeformed mafic crust formed by the breakup of the Ancestral Pilbara Block (APB) proto-cratonic plateau at 3.18 Ga^11^. In addition to boninites, the Whundo Group includes other lithologies commonly associated with modern subduction zones, including the complete calc-alkaline basalt-andesite-dacite-rhyolite spectrum, high-Mg andesites and dacites, adakites, and niobium-enriched basalts^26^ (Fig. 1b). We present high-resolution major and trace element data for a 10-km thick chemostratigraphic succession of volcanic rocks spanning a 30-million-year timespan. In particular, as a result of the relatively low metamorphic grade, testified by alteration screening, the highly fluid-mobile elements in primitive lavas of the Whundo Group retain their primary concentrations (Supplementary Methods, Supplementary Fig. 2, and ref. ^26^), making it an ideal suite to test the hypothesis of Archean dripduction using geochemical modeling.

## Results and discussion

### Whundo primitive lavas

The low metamorphic grade of the Whundo Group is such that the rocks have largely escaped the high degrees of alteration that are typical of rocks older than 3 Ga and fully retain their igneous textures. Many of the basalts are comprised of acicular pyroxenes set in a fine-grained to glassy groundmass, which has been devitrified (Supplementary Fig. 3). Alteration has been largely restricted to locally sericite-dusted plagioclase and minor to moderate degrees of chloritization affecting olivine and (partially) orthopyroxene; clinopyroxene is fully preserved in most samples, which is uncommon for 3.1 Ga rocks. In line with previous findings^26^, alteration screening (Supplementary Methods, Supplementary Fig. 2) suggests that the low degrees of alteration have not substantially affected the large-ion lithophile element (LILE) concentrations of the Whundo lavas, which have values that overlap with modern arc lavas. While there is some compositional scatter, this is expected given the thickness (~10 km), extent (114 km^2^) and age range (20–30 Myr) of the studied package; such scatter is also observed between disparate eruptive centers in modern arcs^27^. We refer the reader to the Supplementary Methods for further discussion on alteration.

**Fig. 2:**
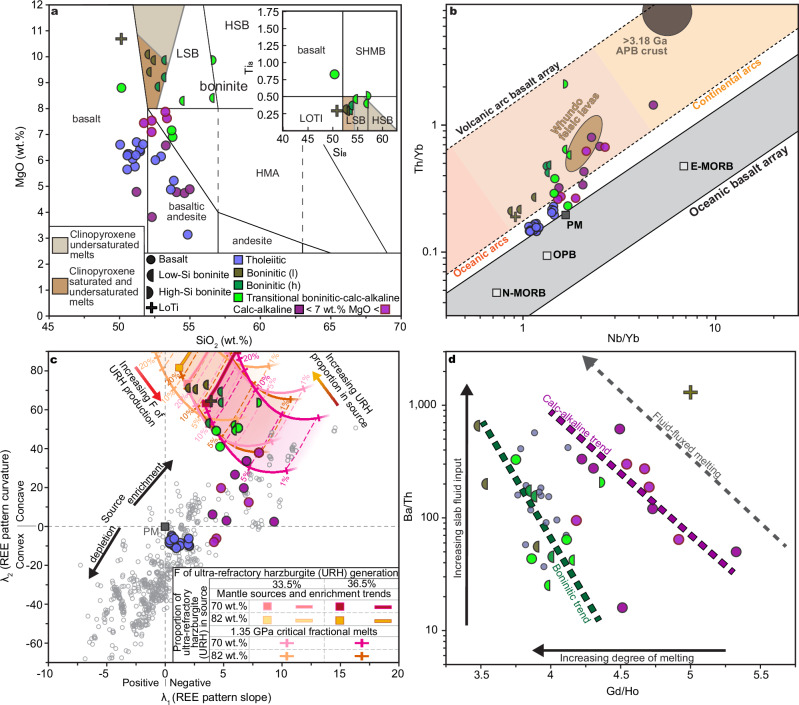
Key characteristics of primitive Whundo Group lavas, including Earth’s oldest extensive boninite suite. **a** Classification of boninites using MgO-SiO_2_ and Ti_8_-Si_8_ (concentrations of SiO_2_ and TiO_2_ for samples with MgO >8 wt.% projected to 8 wt.% MgO; inset) spaces^25^, illustrating that the Whundo boninites represent mixtures of clinopyroxene-saturated and undersaturated melts. **b** Th/Yb-Nb/Yb plot illustrating the signatures observed in Whundo felsic and primitive lavas are irreconcilable with Paleoarchean Ancestral Pilbara Block (APB) crustal contamination and are best explained by a combination of source enrichment and fractional crystallization. >3.18 Ga APB crust and Whundo felsic lava fields are calculated from refs. ^11, 26^, respectively. Arrays and mantle sources are from ref. ^38^ and references therein. **c** Rare earth element (REE) pattern curvature (*λ*_2_) and slope (*λ*_1_) patterns^42^ demonstrating systematics attributed to source enrichment/depletion and partial melting. The lack of covariation between REE shape and MgO for the most primitive calc-alkaline samples (outlined in red) indicates variable enrichment of their mantle source. The patterns observed in the boninitic samples require a hybrid mantle source comprising subordinate ambient upper mantle admixed to a predominant ultra-refractory harzburgite (URH) component, the latter produced by primitive mantle depletion past garnet exhaustion in the garnet stability field. The hydrous critical fractional melting curves for clinopyroxene-poor spinel harzburgite indicate that, depending on the amount of dripduction enrichment, 5–20% melting of a hybrid source containing 70–82 wt.% of the URH component, itself produced by 33.5–36.5% depletion of primitive mantle along a mantle potential temperature (*T*_P_) adiabat of 1605 °C, reproduces the patterns of the boninites. Also shown are the opposing effects of varying URH proportions and *F* of URH production. Open gray circles represent the global array of modern ocean floor basalts^101^. **d** The negative covariations observed in Ba/Th vs. Gd/Ho for the boninitic and calc-alkaline samples are consistent with fluid-fluxed melting, a hallmark of magma generation in subduction zones^27,41,50,51^. However, the tholeiites do not exhibit these covariations, suggesting flux melting was insignificant in their petrogenesis. Abbreviations: HMA – high-Mg andesite, HSB – high-Si boninite, LOTI—low-Ti basalt, LSB – low-Si boninite, OPB – oceanic plateau basalt, PM – primitive mantle.

**Fig. 3:**
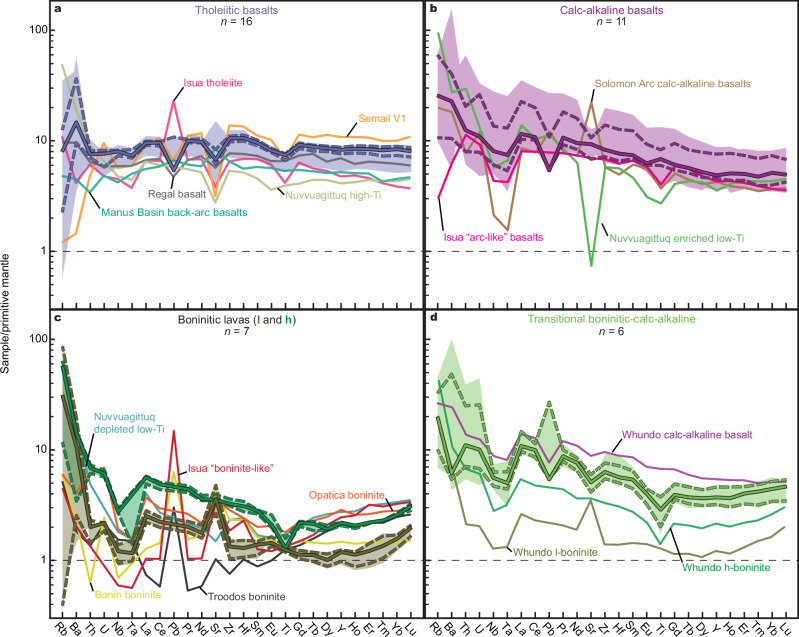
Trace element patterns of primitive Whundo Group lavas compared to selected average lithologies. Primitive mantle-normalized^47^ trace element patterns of tholeiitic basalts (**a**), calc-alkaline basalts (**b**), boninites (**c**), and transitional boninite-calc-alkaline lavas (**d**). The solid and dashed lines represent the medians and 75th (upper) and 25th percentiles (lower) of each magmatic series, respectively, whereas the shaded fields indicate their range. The tholeiitic basalts best resemble Regal Formation basalts from the Regal Terrane of the Pilbara Craton^28,44^, with characteristics similar to those from the V1 series of the Semail Ophiolite^29^. Calc-alkaline basalts share similar features to Solomon Arc calc-alkaline basalts^34^, >3.8 Ga Nuvvuagittuq enriched low-Ti basalts^31^ and c. 3.8 Ga Isua “arc-like” basalts^35^. The LILE-HFSE-L-MREE patterns of the Whundo boninites best resemble those of the c. 2.8 Ga Frotet-Evans (Opatica) boninites^102^ but with spoon-shaped HREE patterns (inflection at Dy), suggesting a unique petrogenesis involving garnet in the mantle source prior to depletion (see Fig. 4). The trace element patterns of transitional boninitic-calc-alkaline samples suggest that they represent magma mixtures of calc-alkaline basalts and l-boninites. Abbreviations: LOTI – low-Ti basalt. Nuvvuagittuq lithologies are from ref. ^31^, Isua tholeiites and “boninite-like” lithologies are from refs. ^30, 103^, respectively, low-Si Troodos boninite is from ref. ^46^, and low-Si Bonin boninites are from ref. ^93^.

The primitive lavas of the Whundo Group (*n* = 40) can be geochemically divided into three distinct magmatic series—tholeiitic, calc-alkaline, and boninitic (Figs. 2, 3, Supplementary Figs. 4–6)—that resemble primitive melts observed in modern oceanic arc-back-arc systems^27^. The complete geochemical classification workflow for the samples is outlined in the Supplementary Methods, and the sample information and geochemical data are presented in Supplementary Data 1.

The tholeiitic basalts (*n* = 16) are easily distinguished from other primitive lavas based on their relatively flat primitive mantle-normalized trace element patterns that lack significant enrichment or depletion (Figs. 2, 3a, Supplementary Fig. 6a). Although tholeiitic basalts are not indicative of any specific setting, their trace element patterns can constrain the petrogenetic processes diagnostic of tectonomagmatic settings. The Whundo tholeiites resemble melts associated with an extensional environment, such as modern-day Manus back-arc basalts^27^ and the APB breakup-associated 3.18 Ga Regal basalts^28^, with some minor characteristics like those of the Semail Ophiolite basalts^29^ (i.e., pronounced negative Pb, Sr and Ti anomalies, unfractionated medium- to heavy rare earth element [MREE to HREE] patterns). There is little resemblance between the Whundo tholeiites and other early Earth counterparts with subduction-like affinities, such as Isua (3.8 Ga North Atlantic Craton^30^) and Nuvvuagittuq (>3.8 Ga Superior Craton^31^), both of which have negative Nb-Ta anomalies^16,32^ (Fig. 3a). Therefore, the lack of resemblance to arc tholeiites suggests they formed in a divergent setting.

The boninitic samples (*n* = 13) are further subdivided into subgroups based on their trace element systematics. This includes high (h; *n* = 3) and low (l; *n* = 4) trace element concentrations, along with mixing products with calc-alkaline magmas (*n* = 6). Although some of these samples were previously referred to as “boninite-like”^26,33^, we demonstrate that the h- and l-boninite subgroups comprise true low-Si boninites (Fig. 2a), which makes them the oldest extensive examples of boninites on Earth. Previous work based on their major element and Nb/Yb-Th/Yb systematics has shown that low-Si arc-basin boninites may be the Whundo boninites’ closest Phanerozoic analogs^25^. These are the most primitive lavas in the Whundo Group and have distinctive trace element patterns; lower in abundance than their tholeiitic and calc-alkaline counterparts, with slight light rare earth element (LREE) and Th enrichment relative to MREE, pronounced negative Nb-Ta anomalies, and distinctive concave-up HREE patterns (Figs. 2c, 3c, Supplementary Fig. 6c). Notably, these patterns barely resemble their Phanerozoic counterparts, but rather more closely resemble other Archean boninite-like lavas. However, Whundo boninites are still unique amongst Archean boninite-like lavas in displaying spoon-shaped HREE patterns, suggesting derivation from a unique mantle source.

The transitional boninitic-calc-alkaline subgroup ranges in composition from high- and low-Si boninite to high-Mg basalt. These lavas are intermediaries between h-boninites and calc-alkaline basalts (Figs. 2c, 3d; Supplementary Fig. 4). Compared to their h-boninitic counterparts, the transitional boninites display distinctive trace element patterns that are more enriched, with significant enrichment in Th and La relative to M-HREE, larger negative Nb-Ta anomalies, and weaker concave-up dish-shaped HREE patterns. On the other hand, the trace element patterns of the transitional basalts exhibit many of the same features as the boninites, albeit more subdued with less Th enrichment (Fig. 3d, Supplementary Fig. 6d). As discussed below, these patterns suggest that this subgroup likely represents mixing of boninitic and calc-alkaline primitive magmas.

The calc-alkaline basalts (*n* = 11) are, on average, more primitive than their tholeiitic counterparts (Fig. 2a, Supplementary Figs. 5–7). They are markedly enriched in incompatible trace elements and depleted in HREE, with primitive mantle-normalized trace element patterns characterized by slight LREE and Th enrichment relative to MREE and slight negative Nb-Ta anomalies (Fig. 3b, Supplementary Fig. 6b). Calc-alkaline (*sensu lato*) basalts are typically found in subduction zones and are more constrained in setting than tholeiites. Our samples share similar geochemical features to modern calc-alkaline basalts^34^ and Eoarchean “arc-like” basalts from Nuvvuagittuq^31^ and Isua^35^, with higher trace element abundances (Fig. 3b). Concerning modern arc basalts, this difference may reflect derivation from a less depleted mantle source. In contrast, the higher average MgO content of comparative Eoarchean samples suggests lower degrees of melting or greater fractionation for the Whundo samples.

Unlike many Archean basalts, evidence for crustal contamination by evolved proto-cratonic basement in the Whundo Group lavas is scarce. The absence of >3.3 Ga Nd isotopic model ages^28^ and >3.25 Ga inherited zircons in the Whundo felsic lavas and later intrusions in the area^26,36^ are consistent with a thin, juvenile basement produced by rifting of the APB at 3.18 Ga. This is also observed in craton-scale variations in lithospheric thickness^37^. Furthermore, the boninites and tholeiitic basalts exhibit array-parallel trends on a Th/Yb-Nb/Yb diagram, inconsistent with crustal contamination (Fig. 2b). While the calc-alkaline basalts exhibit an oblique Th/Nb trend, similarities between the Th/Yb-Nb/Yb systematics of the primitive and felsic^26^ lavas (Fig. 2b) suggest they were not contaminated by ancient crust and therefore, erupted off-plateau. While some degree of contamination by coeval felsic units cannot be ruled out, contamination-sensitive proxies lack covariations with increasing differentiation, indicating that contamination was not a major contributor to the incompatible element budgets of the primitive lavas (Supplementary Fig. 7). Furthermore, some calc-alkaline basalts with the highest MgO also have the highest Th/Nb, thus reconcilable with variable mantle wedge enrichment (Fig. 2b). Therefore, the Th-LREE enrichments and Nb-Ta depletions observed in the boninitic and calc-alkaline lavas likely result from the mantle source’s characteristics, specifically enrichment^25,26,28,36,38,39^.

### Decompression and flux melting explain the origins of Whundo tholeiitic and calc-alkaline basalts

Understanding the origins of the tholeiitic and calc-alkaline basalts requires examination of melt production processes and mantle sources, including their depletion/re-enrichment histories and geochemical budgets. The role of fluids/melts in the melting process can be studied by comparing petrogenetic trace element ratios that track fluid enrichment (e.g. Cs/La, Ba/Th) to those that monitor the degree of melting (e.g., Gd/Ho)^26,40^. Evidence from trace element ratios suggests that the melting style of the calc-alkaline mantle source was affected by higher degrees of metasomatic enrichment. Negative covariations of Ba/Th and Cs/La vs. Gd/Ho indicate that the calc-alkaline magmas were produced by fluxed melting. In contrast, the lack of covariation observed in the tholeiites suggests they were generated by decompression melting (Fig. 2d, Supplementary Fig. 8c). The assertion of fluxed melting is further supported by the trends observed on plots of K_2_O vs K_2_O/Na_2_O and Yb vs Zr, which suggest a fluid-, rather than melt-dominated “slab” component in the source of most Whundo lavas (Supplementary Fig. 8a, b). Fluxed melting is unusual for Archean basalts, which were generally produced by anhydrous melting^19^, but it is a hallmark process in modern subduction settings^27,41^. Our data indicate that fluxed melting was crucial in producing the primitive calc-alkaline magmas throughout the stratigraphy. The constant introduction of fluids/melts to the mantle source for 30 million years implicates significant lithospheric recycling.

Geochemical data demonstrate that the tholeiitic and calc-alkaline melt groups originate from a similar spinel facies mantle source. Both melt types have higher Th/Nb than N-MORB, indicating that the maximum level of prior mantle depletion for the tholeiitic and calc-alkaline sources was less than that of modern depleted mantle (Fig. 2b). This is also reflected in the fact that the primitive mantle-normalized rare earth element (REE) shape parameters (*λ*_1_-*λ*_2_)^42^ for both magmatic series is higher than most modern MORB (Fig. 2c). Mantle enrichment, as indicated by λ_1_-λ_2_ and element anomalies (Fig. 2c, Supplementary Fig. 8d), can explain the differences between the two melt groups. With the tholeiites, the limited range in these parameters and their nearly constant Th/Nb below the arc array (Fig. 2b) suggest derivation from a source with slightly higher degrees of depletion and less re-enrichment. The calc-alkaline lavas exhibit variable, but higher *λ*_1_–*λ*_2_ than the tholeiites. The absence of correlation between REE shape and MgO for the most primitive calc-alkaline samples (>7 wt.% MgO) is explained by variable metasomatic enrichment of the mantle source, and clinopyroxene fractionation during magmatic ascent (Fig. 2c, Supplementary Fig. 8d). Variable enrichment also accounts for the highly variable Th/Nb observed in the calc-alkaline basalts (Fig. 2b, Supplementary Fig. 7), without implicating ancient crustal contamination.

We employed a two-stage modeling approach to investigate the geochemical budgets of the Whundo mantle sources. First, we corrected representative samples from each group for fractional crystallization back to equilibrium with mantle olivine (Fo_86–90_), which enabled us to calculate the trace element compositions of the parental melts. We then used the trace element composition of the melts to back-calculate the composition of the modified mantle source, assuming anhydrous (tholeiitic) and hydrous (calc-alkaline) critical fractional melting. HREE budgets are fluid-immobile and unaffected by subduction enrichment, so we matched the calculated HREE abundances of the modified mantle sources with the melting residues of a primitive mantle source along a mantle potential temperature (*T*_P_) adiabat, in this case 1400 °C. This step was performed using partition coefficient and phase relationship data informed by thermodynamic modeling. This allowed us to estimate the amount of mantle wedge depletion before dripduction and characterize the contribution of the subduction component to mantle budgets of incompatible trace elements. Further details can be found in the Methods, Supplementary Methods, Supplementary Figs. 10, 11, and Supplementary Data 3–5.

**Fig. 4:**
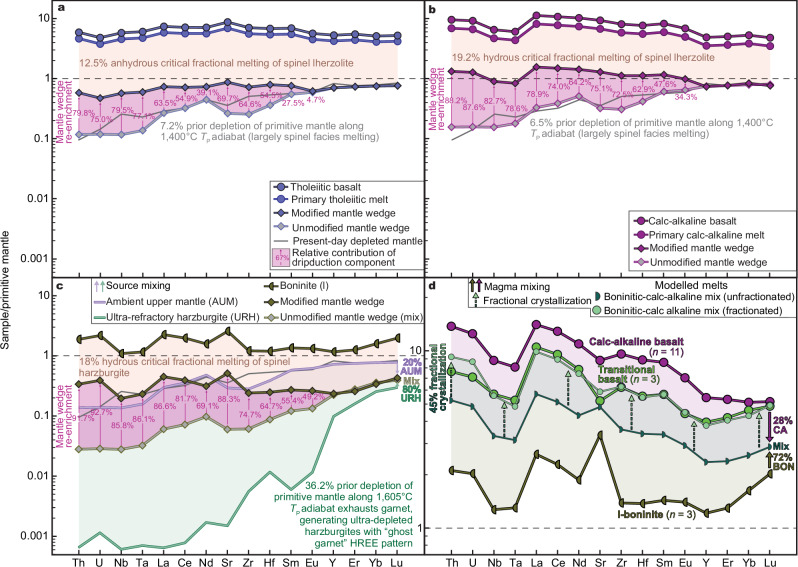
Two-stage mantle melting, subduction enrichment, and mixing to produce primitive Whundo magmas. Primitive mantle-normalized^47^ multi-element plot of incompatible elements showing the compositions of dripduction-modified and unmodified mantle wedge sources, and relative dripduction contributions (percentages) determined using inverse modeling for Whundo tholeiites (**a**), calc-alkaline basalts (**b**), and boninites (**c**). The tholeiitic and calc-alkaline basalts share a nearly identical dripduction-unmodified mantle source (slight differences in depletion), with differences arising from the degree of re-enrichment (and therefore the style of melting), and degree of melting. On the other hand, the concave-up (“ghost garnet”) HREE patterns observed in the modified mantle wedge of Whundo boninites require a unique pre-dripduction mantle wedge source predominantly comprised of ultra-depleted clinopyroxene-free harzburgitic residues produced by melting past garnet-out in the garnet stability field along a high mantle potential temperature (*T*_P_) adiabat. These residues were being transported upwards to the spinel stability field, where 20 wt.% ambient upper mantle is admixed to the residues (i.e., the average of the unmodified calc-alkaline and tholeiitic mantle wedge compositions) to produce a hybrid clinopyroxene-poor harzburgitic mantle source, before finally undergoing modification and melting. The substantial re-enrichment component contributions to the modified mantle wedge source of the Whundo boninites are consistent with other studies on subduction (e.g., ref. ^52^), ruling out upwelling-associated enrichment of an ultra-depleted source (i.e., ref. ^46^). **d** the origin of transitional boninitic-calc-alkaline basalt by mixing 72 wt.% l-boninite with 28 wt.% calc-alkaline basalt, followed by 45% fractional crystallization of a 34% plagioclase –34% orthopyroxene – 26% clinopyroxene – 3% spinel – 3% titanomagnetite assemblage. Depleted MORB mantle (DMM) composition is from ref. ^48^. Modeling details are provided in the Methods, Supplementary Methods, Supplementary Fig. 10, and Supplementary Data 3–10.

Based on mass balance, our calculations demonstrate that the unmodified tholeiitic mantle source was slightly more depleted than the unmodified calc-alkaline source. However, the modified calc-alkaline source experienced greater enrichment (Fig. 4a, b); the source’s lower re-enrichment in Nb and Ta relative to Th, U and La, suggests a predominant slab fluid component, which is consistent with our findings of fluid-fluxed melting. In contrast, the lack of negative high field strength element (HFSE) anomalies, particularly Nb and Ta, in the modeled tholeiitic source suggests re-enrichment by slab-derived melts, a common component in modern subduction zones^43^. Therefore, our results suggest that the mantle sources underwent slight melt-dominated refertilization and decompression melting to generate tholeiitic melts, while greater fluid-dominated refertilization and fluxed melting produced calc-alkaline melts.

### Formation of boninites by fluxing of a garnet-exhausted mantle source

Similar to modern arc-basin boninites^25^, the parental magmas of the l- and h-boninites in the Whundo Group were pooled melts consisting of a subordinate contribution of clinopyroxene-undersaturated melts relative to clinopyroxene-saturated melts (Fig. 2a). The negative covariations observed between fluid enrichment and melting extent proxies suggest a fluxed melting origin, similar to the calc-alkaline samples (Fig. 2d, Supplementary Fig. 8c). Mixing of these melts with calc-alkaline basalts provides a viable origin for the transitional boninitic-calc-alkaline basalts. These samples form an array between the h-boninites and the calc-alkaline cluster in trace element parameters that measure source, melting, and ascent processes (Fig. 2c, d, Supplementary Fig. 4). Based on our data, the average trace element patterns observed in transitional basalts can be replicated by mixing l-boninite with 28 wt.% calc-alkaline basalt, followed by 45% fractional crystallization of a plagioclase-orthopyroxene-clinopyroxene-magnetite assemblage (Fig. 4d).

The boninitic melts were generated in a unique mantle source based on their rare earth element shapes, arrays in element anomaly space, and diagnostic concave-up spoon-shaped HREE patterns (Figs. 2–4, Supplementary Fig. 8d). Based on our two-stage melt modeling of l-boninite, this mantle source consists of two components. The first is a predominant ultra-refractory harzburgite (URH) component that originates as the melt-bearing residue of primitive mantle melting along a 1605 °C *T*_P_ adiabat, where garnet is exhausted in the garnet stability field. The second, a subordinate ambient upper (spinel-facies) mantle component, is the average of the calc-alkaline and tholeiitic unmodified mantle sources and is admixed to the URH component. This produces a hybrid clinopyroxene-poor harzburgitic mantle source that can generate melts in the spinel stability field with the inverse garnet-residual HREE patterns observed in our samples (Fig. 4c). These patterns, here referred to as “ghost garnet” signatures, are not observed in modern boninites. There are two possible reasons for the lack of resemblance with Phanerozoic boninites: (i) the lower average degree of mantle depletion in the Archean, particularly beneath the Pilbara Craton^44^, results in a less refractory bulk source than modern boninites, despite the URH component; and/or (ii) higher mantle potential temperatures in the Archean increased the onset depth and extent of melting in the garnet stability field during decompression melting^33^, enabling garnet exhaustion in the URH component. Our mantle melting models in REE shape (*λ*_1_-*λ*_2_) space indicate the Whundo boninites were derived from 6 to 20% melting of the hybrid clinopyroxene-poor harzburgitic mantle source. These models illustrate that, depending on the amount of re-enrichment to produce the modified mantle source, a hybrid mantle source consisting of 70–82 wt.% of the URH component, itself the residue of 33.5–36.5% melting along the 1605 °C *T*_P_ adiabat, can explain the observed patterns of the Whundo boninites (Fig. 2c).

These melting curves also illustrate that the specifics of this model are, to some degree, non-unique, as decreasing the amount of the URH component can be compensated by decreasing the degree of melting to explain individual samples (Fig. 2c). Indeed, it is important to stress that other models are possible. For example, two-stage melt modeling can also reproduce the patterns observed in the l-boninites if a melt-free refractory harzburgitic solid, produced by high degree melting (past garnet exhaustion) of primitive mantle along a 1610 °C *T*_P_ adiabat, is metasomatized by a small amount (<4 wt.%) of primitive tholeiitic melt (Supplementary Fig. 9a). However, it is evident that several key conditions need to be met, irrespective of the model, to produce the Whundo boninites. These include: (i) a clinopyroxene-poor to exhausted harzburgitic mantle source; (ii) a highly refractory component in the mantle source (comprising either the majority of the source or its entirety) that underwent a prior high-degree depletion event past garnet exhaustion deep in the garnet stability field; and (iii) re-enrichment of the depleted mantle source by a Th-U-LILE-LREE-rich, Nb-Ta- depleted metasomatic agent (Fig. 4c, Supplementary Fig. 9a).

The origins of some pre-3 Ga boninite-like rocks are ascribed to the upwelling-associated mixing of low-degree primitive mantle melts with high-degree melts of ultra-depleted mantle^25,45^, but our samples exhibit evidence of fluxed melting, and have Th/Nb above primitive mantle. Likewise, we can rule out another intraplate origin involving re-enrichment of an ultra-depleted source (e.g., Manihiki^46^) by enriched, OIB-like melts based on our samples’ negative Nb-Ta troughs and MREE depletions. Our two-stage modeling also demonstrates the dominant contribution of metasomatic fluid agents to the incompatible trace element budget of the harzburgitic source of the boninites (up to 93%; Fig. 4c). Pronounced enrichments in fluid-mobile elements (e.g., Sr), even in the alternative model, rule out purely melt re-fertilization of a depleted source. Thus, the Whundo boninites originated from shallow fluxed melting of a hybrid mantle source predominantly comprised of ultra-refractory, garnet-exhausted harzburgite residues; some of these melts variably with calc-alkaline melts during ascent, producing the transitional basalts.

### Off-plateau dripduction as a possible mode of Archean hydrosphere–asthenosphere interaction

**Fig. 5:**
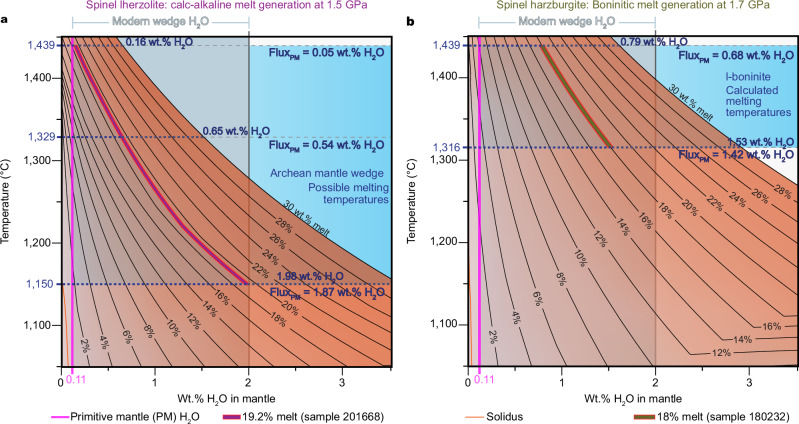
*T*-*X* diagrams demonstrate that the Whundo calc-alkaline basalts and boninites require the addition of water by dripduction. **a** The addition of at least between 0.05 and 1.87 wt.% H_2_O is necessary to produce a 19.2% melt of near-primitive spinel lherzolite at 1.50 GPa under reasonable sub-arc mantle wedge temperatures. This is greater than the water content of the primitive mantle (0.11 wt.% H_2_O)^47^ and well within the range of the mantle water contents of modern subduction zones (0.11–2.00 wt.% H_2_O)^50,51^, requiring the efficient transfer of water from the crust into the mantle. **b** The origins of the most-primitive l-boninites, for which melting temperatures and pressures can be independently calculated (see Methods), provide much more robust constraints on the amount of water in the Whundo sub-arc mantle. Here, the addition of at least between 0.68 and 1.42 wt.% H_2_O is required to produce a 18% flux melt of clinopyroxene-poor spinel harzburgite at 1.70 GPa. Ignoring H_2_O storage capacity in nominally anhydrous minerals (e.g., olivine, clinopyroxene, orthopyroxene), Flux_PM_ denotes the maximum possible H_2_O flux, i.e., water exceeding primitive mantle water content. As a result of H_2_O storage in nominally anhydrous minerals, the actual H_2_O contents will be slightly higher and therefore all H_2_O values presented here represent minimum estimates (see “Methods” and “Supplementary Methods”). The lower bound of subarc mantle wedge temperatures in (**a**) is inferred from the numerical models of ref. ^104^, whereas the upper bound corresponds to the maximum temperature of primary melt generation inferred for (**b**). Modeling details are provided in the Methods, Supplementary Methods, Supplementary Fig. 11, and Supplementary Data 6.

To produce the calc-alkaline basalts at 1.5 GPa, thermodynamic modeling suggests the mantle must at least contain between 0.16 and 1.98 wt.% H_2_O (Fig. 5a), which is higher than the primitive mantle (0.11 wt.% H_2_O)^47^ and at least an order of magnitude above depleted mantle (~0.01 wt.% H_2_O)^48,49^. A more substantial constraint, however, is the water input required to produce the boninites, for which the *P*-*T* conditions of melting can be independently estimated; flux melting of spinel harzburgite at 1.70 GPa requires a water content of at least 0.79 to 1.53 wt.% H_2_O to produce the most primitive l-boninites (Fig. 5b). Using the composition of the harzburgite from the alternative boninite model produces a similar result (0.85 to 1.57 wt.% H_2_O; Supplementary Fig. 9b). Although these models do not account for water stored in nominally anhydrous minerals (e.g., olivine, clinopyroxene, orthopyroxene), which can incorporate H_2_O on the order of several hundreds of ppm^49^, this means that the actual H_2_O contents will be slightly higher. Therefore, these results can be faithfully interpreted to represent a minimum estimate of the sub-Whundo mantle water contents. Crucially, these estimates are within the range of the water contents of mantle wedge peridotite (0.1–2.0 wt.% H_2_O)^50,51^ in modern subduction zones.

However, higher mantle temperatures predicted for the early Earth suggest a weaker crust, precluding Benioff-type subduction^17–19^ and instead favor dripduction, which geochemically resembles modern subduction. Based upon the evidence of protracted fluxed melting recorded in the calc-alkaline and boninitic lavas, magmatic diversity, and degrees of enrichment, the data indeed resemble the range of modern hot-subduction zone boninites and arc basalts^52^, which in Archean time is best explained by dripduction^17–20^. Dripduction is characterized by poorly defined plate margins that permit intermittent subduction, where the ductile down-going plate drips rather than sinking as a rigid slab^19^. Indeed, Archean off-plateau dripduction processes re-enriched the mantle to levels similar to those observed in modern subduction zones^53^. The diversity of magmas modeled here and assigned to dripduction zones reflects the pre-enrichment heterogeneity of the Archean mantle; near-primitive spinel facies lherzolite underwent adiabatic and fluxed melting to produce tholeiitic and calc-alkaline basalts, while refractory garnet-exhausted harzburgite underwent fluxed melting to generate the oldest stratigraphically extensive boninite lavas on Earth.

**Fig. 6:**
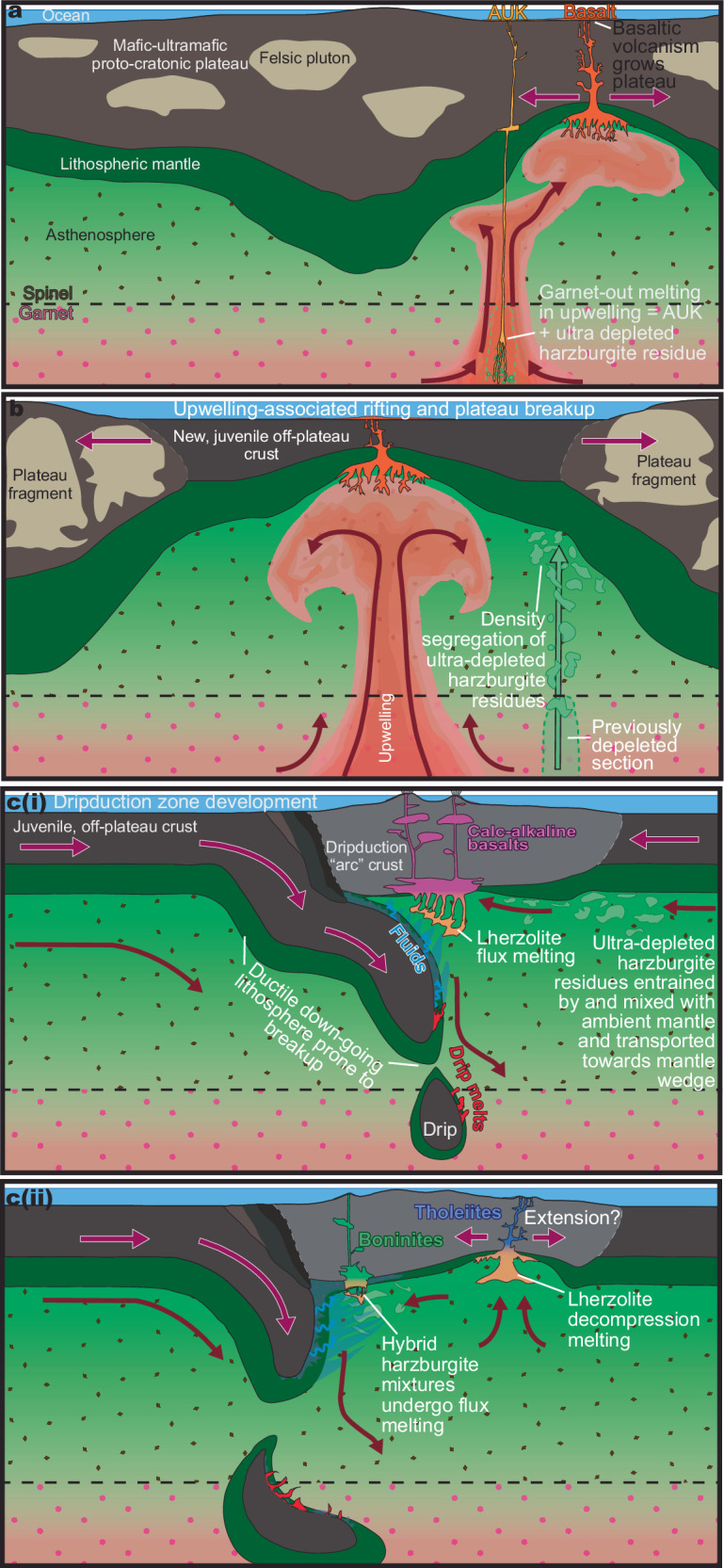
Schematic cartoon of Archean dripduction primitive lava petrogenesis and the origins of Mesoarchean boninites. **a** Upwellings beneath mafic-ultramafic proto-cratonic plateaux predominantly grow the plateau through extensive basaltic volcanism^20,55^ generated at relatively shallow mantle depths but also produce aluminum-undepleted komatiites (AUK) through melting past the point of garnet exhaustion at greater depths^56^, leaving ultra-depleted clinopyroxene-free harzburgite residues with “ghost garnet” signatures. **b** Due to their lower density than the surrounding garnet-bearing ambient mantle, the harzburgites segregate and move upward toward the top of the asthenosphere^57^, possibly being transported away from the proto-cratons through convection. Further upwellings may trigger the breakup of the overlying proto-cratons, producing intervening off-plateau juvenile crust^11,20^. **ci** Tens of millions of years later, a dripduction zone forms within the off-plateau crust, where fluid-fluxed melting of the lherzolitic mantle produces calc-alkaline basalts. Dripduction-induced corner flow of the mantle entrains the ultra-depleted harzburgitic residues, transporting them towards the mantle wedge. Subordinate amounts of ambient mantle are admixed to the ultra-depleted residues, producing hybrid mantle sources with clinopyroxene-poor harzburgitic bulk compositions. **cii** Similar to the modern Tonga arc^59^, boninitic magmas with “ghost garnet” signatures are produced when the entrained harzburgitic hybrid mantle sources enter the arc melting regime and undergo fluxed melting. Local extension triggers decompression melting of re-enriched mantle wedge lherzolite, producing tholeiitic basalts. Note that ci and cii are temporally interchangeable, perhaps even contemporaneous. Panels are not to scale.

The key difference to a modern subduction zone is that the precursory ultra-refractory harzburgite component required to produce some of the lavas sampled here formed deep in the garnet stability field, where primitive fertile mantle underwent very high degrees of melting, in contrast to the slightly depleted spinel lherzolite sources of the calc-alkaline and tholeiitic basalts (i.e., 36.2% vs. 6.5–7.2%; Fig. 4a–c). Given the age of the samples, it is plausible that this refractory reservoir originated from residues generated during early mafic crust production. Earth’s early primitive crust is inferred to have been mafic-ultramafic in composition^54^, formed by extensive volcanism over rifts or mantle upwellings^20,21,55^. Aluminum-undepleted komatiites (AUK), high-degree mantle melts that comprise subordinate proportions of this mafic-ultramafic crust, provide evidence that some of the domains in these upwellings underwent melting past the point of garnet exhaustion^56^, thereby producing ultra-refractory harzburgites with “ghost garnet” signatures (Figs. 2c, 4c, 6a).

As a result of prior melt extraction past the point of garnet exhaustion, garnet-free ultra-refractory harzburgitic residues are less dense than the surrounding ambient mantle, allowing for their upward movement toward the top of the asthenosphere^57^ (Fig. 6b). The subsequent accumulation of the harzburgitic residues at the top of a heterogeneous asthenosphere, where they can mix with ambient upper mantle, then forms the source for boninites during dripduction. Ultimately, these residues may end up in off-plateau settings passively, through the fragmentation of proto-cratonic plateaux, or actively, by convective transport in the upper mantle. Although the mantle beneath the Pilbara Craton is inferred to have been nearly chondritic up to 3.2 Ga^44^, there is isotopic evidence for small volumes of refractory domains^58^. In addition, Eoarchean boninite-like rocks from Isua notably have strongly radiogenic Hf isotope signatures, pointing to a longer-term depletion of their sources^32^.

Irrespective of the mantle composition, once an off-plateau dripduction zone develops, efficient lithosphere-asthenosphere interaction begins. The ductile down-going crustal sections release fluids and melts that induce flux melting in the surrounding mantle (Fig. 6ci), providing up to 93% of the element budget of the wedge (Fig. 4). The mantle mixing induced by dripduction entrains harzburgites into the mantle wedge, promoting mantle heterogeneity. Like modern convergent margins^59^, heterogeneous mantle flow likely plays an essential role in controlling primitive magma compositions in Archean dripduction systems, albeit with a distinct outcome. Calc-alkaline basalts form when the arc melting regime comprises relatively undepleted lherzolite (Fig. 6ci). When the URH-dominated hybrid mantle source enters the dripduction arc melting regime, fluxed melting produces boninites exhibiting the “ghost garnet” memory of the source’s prior depletion history (Fig. 6cii). If the dripduction arc melting regime also contains lherzolite during this time, the resultant calc-alkaline basalts will mix with boninites, producing the transitional boninitic-calc alkaline basalts and boninites.

The tholeiites are likely derived from a refertilized lherzolite source that underwent decompression melting in response to local extension (Fig. 6cii). The enrichment inferred in their source is consistent with lithosphere-asthenosphere interaction, as indicated by elevated Th/Nb at near-constant HREE and the absence of HFSE troughs. Similar proximal contrasts of melting regimes are relatively common in Phanerozoic volcanic arcs^40,60,61^.

As evidenced by the varied, albeit weakly cyclic stratigraphy of the Whundo Group (Fig. 1b), it is important to recognize that these primitive lavas, particularly the calc-alkaline basalts, boninites and transitionals, were erupted diachronously. This contrasts heavily with the typical uniformity and stratigraphic progression from tholeiite to boninite and finally to calc-alkaline BADR lavas that is characteristic of subduction initiation recorded in Phanerozoic forearcs and ophiolites^24,25^. Thus, given the available evidence, including the similarities between the Whundo boninites and their modern arc-basin counterparts^25^, Whundo is unlikely to be an Archean analog of subduction initiation in a forearc setting. Understanding this stratigraphic variability and cyclicity is beyond the scope of this study, but these observations remain reconcilable with Whundo as an Archean dripduction analog of another portion of a convergent margin setting, such as an arc-front or arc-basin setting, where different lavas erupt diachronously^25,27^, even during subduction initiation^62^.

A final note is needed on the often-proposed and widespread mechanism of lower crustal delamination. These sections are very different from the modeled environment of a dripduction zone and must be treated separately. Although lower crustal drips at the base of proto-cratons can facilitate interaction between the lithosphere and asthenosphere^10,20,63^, these drips are unlikely to be the primary source of water required for inducing fluxed melting of the mantle as modeled here. This is because the necessary crustal thickness for lower crustal drip occurrence (>35 km)^63^ is beyond the dehydration point of all mafic-ultramafic crustal lithologies^14^ for geotherms consistent with upwellings, which hinders effective crust-mantle water exchange. As a result, fluids are confined to within the crust and, at most, the lithospheric mantle in mafic-ultramafic proto-cratonic plateaux. These are processes commonly associated with TTG or potassic granite formation^10,13–15^. Instead, we stress here that the combination of thinner crust (~25 km)^64^ and lower geotherms^65^ in off-plateau regions, away from upwellings, offers much more favorable conditions for water transfer to the mantle. The maximum permissible gradient for water to enter the asthenosphere is 600 °C/GPa^14^, well within the range calculated for dripduction^65^. Although dripduction-associated water transfer back into the mantle is less efficient than in modern cool-endmember subduction due to the higher geothermal gradients, it was probably significant in regulating the mass balance of lithosphere-asthenosphere volatile exchange. This marks a clear distinction between the volcanic successions studied here and TTGs, which make up the majority of felsic crust in today’s cratons and are frequently, but perhaps falsely, employed to study Archean subduction dynamics.

Our findings suggest that a moderately hydrated lithosphere, comprised of hydrated mafic rocks and perhaps subordinate chemical sediments, has interacted with a heterogeneous asthenosphere since the Mesoarchean Era in the Pilbara, and possibly earlier elsewhere^16,21^. A heterogeneous mantle, composed of variably depleted sections, some of which previously resided in the garnet stability field, flux-melted to produce a range of modern-arc lava types. Notably, the physicochemical nature of the resultant thin, evolved crust likely made it susceptible to later recycling by post-Archean Benioff-style subduction^66^, thus explaining its scarcity in the geological record. The rare sections of this crust that were preserved appear to have been wedged between older or contemporaneous protocratonic plateaus during craton assembly (e.g., refs. ^22,23^). These sections do not resemble plateau-type crust, nor are they related to the loci of typical TTG formation and possible associated volcanism^67,68^.

The return of surface water to the site of Archean mantle melting through dripduction in off-plateau settings was sufficient to achieve vapor-saturated melting comparable to modern mantle wedges under reasonable *P*-*T* conditions (Fig. 5). This implies that volcanism triggered through vapor-saturated melting may have been as effective in influencing and shaping the Archean hydro- and atmosphere as it is today, at least locally. Likewise, the residues of vapor-saturated melting retain a substantial degree of hydration^50,51^, impacting mantle rheology by providing an effective mechanism to counterbalance dehydration stiffening in the upper mantle driven by prior melt extraction^20,49^. This would have led to a variably depleted, re-hydrated and re-enriched mantle, which, after convection, would have partially contributed to a globally depleted mantle. Given that the missing 50–90% of evolved Archean crust^7,8,64^ could be produced by dripduction, this process could have significantly affected the Archean water budget of the crust-upper mantle system, without leaving a significant complementary footprint in preserved crustal sections.

## Methods

### Whole-rock major and trace element analyses

The samples for this study comprise a subset of a laterally extensive reference collection that spans the entire stratigraphy of the Whundo Group at a barcoding resolution (distance between samples of generally 10–50 m, but no more than 300 m) and includes 29 samples from ref. ^26^. that have been reanalyzed, and 29 new samples collected in 2022 (Fig. 1a; Supplementary Data 1). In the field, care was taken to select the least altered portions of outcrops and samples collected were a minimum of 2 kg to ensure representativeness. The samples collected in 2022 were prepared for analysis at Monash University, Clayton, Victoria, Australia. They were cleaned by trimming off weathering rinds and veins with a diamond blade saw before being crushed with a ceramic jaw and disc crusher and milled in an agate ring mill, minimizing contamination during preparation. Both sample sets were analyzed for major, minor and trace element whole-rock geochemistry at Bureau Veritas, Perth, Western Australia. Major and minor element concentrations (Si, Al, Fe, Ti, Mn, Mg, Ca, Na, K, Cr, Sr, Ba, and P) were determined by X-ray fluorescence spectrometry on a fused glass disk and loss on ignition (LOI) by thermogravimetric analysis. The concentrations of Ag, As, Ba, Be, Bi, Cd, Ce, Co, Cr, Cs, Cu, Dy, Er, Eu, Ga, Gd, Ge, Hf, Ho, La, Lu, Mo, Nb, Nd, Ni, Pb, Pr, Rb, Sb, Sc, Sm, Sn, Sr, Ta, Tb, Th, Tl, Tm, U, V, W, Y, Yb, Zn and Zr were all determined by laser ablation ICP-MS on a fragment of the same glass disc previously used for X-ray fluorescence analysis. Total uncertainties for major elements are ≤1.5%, and those for minor elements are <2.5% (at concentrations >0.1 wt.%). Repeat analysis of the OREAS 24b and Bunbury basalt (BB1) reference materials gave relative standard deviation (RSD) values of <5%, and mean concentrations are within 10% of published values for most elements^69,70^. Blindly inserted single analyses of USGS BIR-1 are also within 10% of published values for most elements^71,72^ (Supplementary Data 2). RSD values for duplicate sample analyses are better than 5% for most elements (Supplementary Data 2), and total procedural blanks were negligible relative to analyzed sample concentrations.

### Alteration screening and classification

Before screening and classification, the major element concentrations of samples were re-calculated on an anhydrous basis following ref. ^25^. As a first pass, we excluded samples with LOI > 5 wt.% (approximating the upper limit for magmatic water contents in arcs^50^) from the study, yielding 24 samples from ref. ^26^ and 16 new samples. Next, we screened for metamorphic and weathering effects using alteration box plots^73^ and mafic-felsic-weathering (MFW^74^) ternary diagrams and by testing Rb, Ba and Cs vs Nb, and Rb vs LOI relationships; the results indicate that the samples retained their magmatic LILE budgets at the whole-rock scale (Supplementary Fig. 2). Lithologies were assigned using a modified TAS diagram for LOI < 2% and Zr/Ti vs Nb/Y^75^ for LOI > 2%, with boninites additionally checked on Ti_8_ vs Si_8_ and MgO vs TiO_2_, following ref. ^25^. Magmatic series assignments used λ_2_ vs Ti/V, trace element patterns, and Th/Yb vs Zr/Y^76^. Details are in the Supplementary Methods and Supplementary Figs. 2-5.

After screening and classification, the suites comprise tholeiitic basalts (*n* = 16), calc-alkaline basalts (*n* = 11), boninites (*n* = 7; h and l subsets), and transitional boninitic–calc-alkaline lavas (*n* = 6). Summary element ranges are given in the “Supplementary Methods”, and Supplementary Fig. 5, with individual trace-element patterns in Supplementary Fig. 6.

### Fractional crystallization corrections

The tholeiitic and calc-alkaline basalts are not primary melts (i.e., they do not have MgO/Mg# in equilibrium with mantle olivine^27^), requiring back-correction for fractional crystallization before modeling. To do this, we used the PRIMACALC2 model^77^. We chose the tholeiitic and calc-alkaline samples with the most representative trace element patterns (174474 and 201668, respectively), using parameters guided by crustal thickness and arc/back-arc analogs: tholeiitic 6 kbar, 0.75 wt% H_2_O, ƒO_2_ = FMQ −1.5; calc-alkaline 5 kbar, 4 wt% H_2_O, ƒO_2_ = FMQ + 0.1; *D*^Ni^ followed published olivine–melt calibrations^78^. These back corrections resulted in magmas with 13.4 and 16.8 wt.% MgO, respectively; this model is discussed further in the Supplementary Methods and results and inputs are provided in Supplementary Data 9. For the l-boninites, we chose the sample with the most refractory trace element pattern (i.e., the highest degree of depletion). This sample (180232) has a high MgO concentration (10 wt%) and Mg# (66) approaching that of primary melts, thus not requiring back-correction.

### Thermodynamic modeling

All thermodynamic modeling was performed using version 1.3.4 of the MAGEMinApp software^79^, carried out in the Na_2_O-CaO-K_2_O-FeO-MgO-Al_2_O3-SiO_2_-H_2_O-TiO_2_-Fe_2_O_3_-Cr_2_O_3_ (NCKFMASHTOCr) chemical system using the internally-consistent thermodynamic dataset (version ds636/G25) for igneous systems^80^. For all systems, we used the following activity-composition models: silicate melt^80^; garnet, clinopyroxene, orthopyroxene, and ilmenite/hematite^81^; olivine^82^; feldspars^83^; and spinel group minerals^84^; pure phases included were quartz, rutile and sphene. For hydrous systems, we additionally included the following activity-composition models: fluid and biotite^80^, and clinoamphibole^85^; H_2_O was also considered as a pure phase. Further details are provided below and in the Supplementary Methods; all major element inputs are provided in Supplementary Data 6.

### Modified mantle wedge composition modeling

Partial melting models were constructed using the non-modal pooled fractional melting equation adapted from ref. ^86^:

1 $$\frac{{C}_{{{\rm{l}}}}}{{C}_{0}}=\frac{1}{F}\left(1-{\left(1-\frac{{PF}}{D}\right)}^{\frac{1}{P}}\right)$$

Where *C*_l_ represents the concentration of a given trace element in the melt fraction; *C*_0_ is the concentration of the same trace element in the source; *D* is the bulk partition coefficient of the starting mineral assemblage for the element in question; *P* is the bulk reaction coefficient; and *F* is the degree of melting of the source.

For each element, *D* is calculated from the sum of individual partition coefficients, K_*i*/l_ of each mineral *i*, weighted according to their mass fractions *x*_*i*_:

2 $$D={\sum }_{i=1}^{n}{x}_{i}{K}_{i/{{\rm{l}}}}$$

Similarly, *P* is calculated from the sum of individual partition coefficients weighted according to reaction coefficients *p*_*i*_:

3 $$P={\sum }_{i=1}^{n}{p}_{i}{K}_{i/{{\rm{l}}}}$$

Using a similar approach to refs. ^52^, ^87^, we calculated the incompatible trace element compositions of the modified mantle wedge (*C*_mw_). We first consider two fundamental melting processes involved in producing Whundo Group lava: anhydrous and hydrous critical fractional melting with 2% retained melt^88^. These produce the tholeiitic and calc-alkaline magmas, respectively. Here, the mantle has a critical porosity, so there is always trapped melt present. This is achieved by treating trapped melt as a mineral with *K*_trappedmelt/melt_ = 1 and *p*_trappedmelt_ = 0 (e.g., ref. ^89^). We consider the mineralogy of both magmas’ mantle sources to be identically composed of spinel lherzolite. We utilize the following equation, rearranged from Eq. 1:

4 $${C}_{{{\rm{mw}}}}=\frac{{C}_{1}F}{1-{\left(1-\frac{{PF}}{D}\right)}^{\frac{1}{P}}}$$

where *C*_*1*_ represents the concentration of a given trace element in each sample, *D* is the bulk partition coefficient of the source, *P* is the bulk reaction coefficient of the melting modes, and *F* is the degree of melting of the source.

Because there is no evidence for fluid-fluxed melting in tholeiitic basalts (Fig. 2d, Supplementary Fig. 8), we propose that they are formed by melting processes resembling those observed in modern mid-ocean ridge basalts (e.g., ref. ^90^) or back-arc basin basalts. This simulates relatively dry, adiabatic mantle melting. Therefore, for the tholeiites, we assume anhydrous melting in the spinel stability field at ~1.5 GPa using anhydrous partition coefficients for *D*, with *P* calculated following melting modes from ref. ^91^; and *F* of 12.5%.

The evidence for fluid-fluxed melting in calc-alkaline samples (Fig. 2d, Supplementary Fig. 8) suggests that the primitive parental magmas were formed by hydrous fluid-fluxed melting, similar to what occurs in modern subduction zone mantle wedges (e.g., ref. ^51^). Thus, we assume hydrous melting in the spinel stability field at ~1.5 GPa using hydrous partition coefficients for *D*, with *P* calculated following melting modes of ref. ^92^ and *F* of 19.2%. A full methodology and its underlying rationale can be found in the Supplementary Methods. The sources for all partition coefficients used in this study are provided in Supplementary Data 4.

Modeling the modified mantle wedge sources of the l-boninites is considerably more difficult due to the unique mineralogies of their modified mantle wedge sources. Boninites are generally regarded to be derived from harzburgitic mantle sources (e.g., ref. ^25^); thus, full consumption of clinopyroxene in the source at *F* < *F*_total_, prevents estimation of the mantle wedge in a single-stage back-calculation. As such, this group’s modified mantle source was calculated using a forward modeling approach. We generated a spreadsheet containing a list of possible concentrations in the mantle wedge for each trace element, increasing by 0.001 ppm intervals. For each possible concentration, the composition of the melt was calculated assuming hydrous, non-modal critical fractional melting with 2% retained melt using the following set of equations:

5 $${C}_{{{\rm{a}}}}=\frac{{C}_{{{\rm{mw}}}}}{{F}_{{{\rm{out}}}}}\left(1-{\left(1-\frac{{P}_{1}{F}_{{{\rm{out}}}}}{{D}_{{{\rm{hy}}}1}}\right)}^{\frac{1}{{P}_{1}}}\right)$$

melt composition for melting interval 0 < *F* ≤ *F*_out_, where ${F}_{{{\rm{out}}}}=\frac{{M}_{\left({\mathrm{lim}},F=0\right)}}{{P}_{1}}$ is the degree of melting required for limiting mineral exhaustion.

6 $${M}_{\left(i,{F}_{{{\rm{out}}}}\right)}=\frac{{M}_{\left(i,F=0\right)}-{F}_{{{\rm{out}}}}{P}_{1}}{1-{F}_{{{\rm{out}}}}}$$

recalculated mass mode of mineral *i* in source at *F* = *F*_out_.

7 $${C}_{{F}_{{{\rm{out}}}}}=\frac{{C}_{{{\rm{mw}}}}}{1-{F}_{{{\rm{out}}}}}\left({\left(1-\frac{{P}_{1}{F}_{{out}}}{{D}_{{hy}1}}\right)}^{\frac{1}{{P}_{1}}}\right)$$

concentration of source at *F* = *F*_out_.

8 $${C}_{{{\rm{b}}}}=\frac{{C}_{{F}_{{{\rm{out}}}}}}{({F}_{{{\rm{total}}}}-{F}_{{{\rm{out}}}})}\left({1-\left(1-\frac{{P}_{2}({F}_{{{\rm{total}}}}-{F}_{{{\rm{out}}}})}{{D}_{{{\rm{hy}}}2}}\right)}^{\frac{1}{{P}_{2}}}\right)$$

melt composition for melting interval *F*_out_ < *F* ≤ *F*_total_.

9 $${C}_{{{\rm{l}}}-{{\rm{calc}}}}=\frac{\left({F}_{{{\rm{out}}}}{C}_{{{\rm{a}}}}\right)+{C}_{{{\rm{b}}}}\left({F}_{{{\rm{total}}}}-{F}_{{{\rm{out}}}}\right)}{{F}_{{{\rm{total}}}}}$$

total melt composition.

Where *C*_mw_ is the original elemental concentration in the modified mantle wedge, *F*_out_ is the degree of melting required to exhaust the limiting mineral, *D*_hy1_ is the bulk partition coefficient for hydrous partial melting of the source before limiting mineral exhaustion, *P*_1_ is the bulk reaction coefficient of the melting reaction before limiting mineral exhaustion, *M*_lim_ is the initial mass mode of the limiting mineral, *M*_*(i, F*=0*)*_ is the initial mass mode of a given mineral in the source, *F*_total_ is the total degrees of melting, *D*_hy2_ is the bulk partition coefficient for hydrous partial melting of the source with modes calculated in Eq. 6 for the second interval of melting, and *P*_2_ is the bulk reaction coefficient of the melting reaction after the limiting mineral exhaustion point. The spreadsheet then finds where *C*_l-calc_ = *C*_l_ for a given trace element and returns its corresponding *C*_mw_ value, therefore yielding the elemental composition of the modified mantle source.

To model the depleted harzburgitic source inferred for l-boninitic primitive magmas, we used an initial source mineralogy from ref. ^87^, where *P*_1_ is the hydrous spinel facies melting modes from ref. ^92^, clinopyroxene-exhausted *P*_2_ modes follow ref. ^93^, and *F*_total_ is assumed to be 18%. In this case, *C*_1_ is the trace element concentration of l-boninite sample 180232. More details are provided in the Supplementary Methods and the flowchart in Supplementary Fig. 10. All parameters and results are presented in Supplementary Data 3, partition coefficients are listed in Supplementary Data 4, and calculation spreadsheets are provided in Supplementary Data 5.

### Pre-dripduction mantle wedge composition modeling

The pre-dripduction mantle wedge is the mantle source of the primitive lavas before any dripduction-related input, assuming a two-stage melting process. The heavy rare earth element (HREE) budget of the primitive lavas should not be affected by dripduction processes, so we assumed the pre-dripduction mantle wedge HREE budget was similar to that of the modified mantle wedge (e.g., refs. ^52,87^). Unlike the inverse modeling approach required to calculate the modified mantle wedge compositions, the use of a forward modeling approach in this step enables the incorporation of thermodynamic models of mantle decompression melting, providing phase proportions necessary for trace element modeling and major element compositions of the system, minerals, and melts that can be used to calculate partition coefficients that vary as melting progresses.

For thermodynamic modeling, we use the estimated major element composition of the primitive mantle from ref. ^94^ as our starting composition. We first determined the pressure-temperature paths for several mantle potential temperature (*T*_P_) adiabats using the PTX interface set to adiabatic equilibrium melting, with melt deselected, ensuring the melt extraction threshold had not been crossed at the starting pressures. Using the resultant *P*-*T* points, we then model fractional melting along the prescribed *T*_P_ adiabats using the fractional melting mode with the melt phase reselected and the melt extraction threshold determined by the pressure at which the path crosses the solidus (see Supplementary Methods).

Using these outputs, we determine the incompatible trace-element compositions of the pre-dripduction mantle wedge (*C*_res_) and the degree of its melt depletion, assuming a starting point of a primitive mantle composition (*C*_0_; ref. ^47^). We use a stepwise approach replicating critical fractional melting with the following set of equations:

10 $${M}_{i}^{{{\rm{res}}}}={M}_{i-1}^{{{\rm{res}}}}-\Delta {{\rm{E}}}{C}_{{{\rm{l}}},i}$$

mass of a given element in the residue at step *i*

11 $${C}_{{{\rm{l}}},i}=\frac{{M}_{i-1}^{{{\rm{res}}}}}{{S}_{i}{D}_{i}+{\phi }_{{{\rm{bef}}},i}}$$

concentration of the element in the melt in equilibrium with the solids at step *i* (12)

12 $${C}_{{{\rm{res}}},i}=\frac{{M}_{i}^{{{\rm{res}}}}}{1-{E}_{i}}$$

concentration of the element in the residue (trapped melt + solids) at step *i* (13)

Where ${M}_{0}^{{{\rm{res}}}}={C}_{0}$, *E*_*i*_ is the fraction of cumulative extracted melt at step *i*, $\Delta {E}_{{i}}={E}_{i}-{E}_{i-1}$ is the instantaneous amount of melt extracted between steps, ${S}_{i}=1-{F}_{i}$ is the solid mass fraction at step *i*, *F*_*i*_ is the total amount of melt at step *i*. ${\phi }_{{{\rm{bef}}},i}={\phi }_{i}+{\Delta {{\rm{E}}}}_{i}$ is the melt present just before extraction at the end of step *i*, and *ϕ*_*i*_ is the fraction of retained melt after extraction at step *i*. Note that in this instance, *D*_*i*_ is the bulk partition coefficient of the solid assemblage at step *i*; hence, unlike with the modified wedge calculations, melt is not included. The individual partition coefficients (*K*_*i*/l_) of each mineral were calculated for each step using the equations/methods listed in Supplementary Data 4.

Here, the degree of melt depletion (*F*) in generating the pre-dripduction mantle wedges is obtained when the HREE concentrations modeled for the pre-dripduction mantle wedges approximately equal those of the modified mantle wedges. This is achieved by minimizing the root mean square of the log ratios of HREE abundances in the unmodified and modified wedge compositions.

For tholeiitic and calc-alkaline lavas, residues produced by fractional melting along a prescribed 1400 °C *T*_P_ adiabat yielded the best fits for the pre-dripduction mantle wedges, where the mantle adiabat crosses the solidus at around 2.42 GPa. The best fit for the unmodified tholeiitic mantle wedge was achieved at an *F* of 7.2%, while the unmodified calc-alkaline mantle wedge required slightly less depletion, at an *F* of 6.5%.

The unmodified mantle source of the boninites requires a mixture of two components to reproduce its unique HREE pattern: a predominant ultra-refractory harzburgite component with a subordinate slightly-depleted lherzolite component. The refractory harzburgite component is produced along a prescribed 1605 °C *T*_P_ adiabat, where melting begins at 6.1 GPa, proceeding to an *F* of 36.2%. The resultant trace element composition of the refractory harzburgite was mixed with 20 wt.% ambient upper mantle (an average of the unmodified tholeiitic and calc-alkaline mantle wedge compositions). This combination of *T*_P_, depletion and mixing proportions yielded the best fit to the modified boninitic mantle wedge.

Major element inputs for the thermodynamic depletion modeling are provided in Supplementary Data 6, major element results are provided in Supplementary Data 7, and trace element results are provided in Supplementary Data 8. A workflow is provided in Supplementary Fig. 10, and details of our calculations, including a full description of the methodology for the alternative boninite model, are found in the Supplementary Methods and Supplementary Data 3.

### Mass balance calculations

The relative contributions of the dripduction components were calculated by taking the difference in incompatible trace element abundances between the modified and pre-dripduction mantle wedges as a percentage relative to the modified wedge abundances using the following equation:

13 $$\Delta C(\%)=\left(\frac{{C}_{{\mathrm{mw}}}-{C}_{{{\rm{s}}}}}{{C}_{{\mathrm{mw}}}}\right)\times 100$$

Where Δ*C* is the contribution of the dripduction component to the budget of a given incompatible trace element, *C*_mw_ is the concentration of that element in the modified mantle wedge, and *C*_s_ is the concentration in the unmodified mantle wedge.

### Magma mixing and fractional crystallization

To produce the patterns observed in the transitional boninitic-calc-alkaline basalts, we mixed geometric means of l-boninite and calc-alkaline basalt in a 72–28 wt.% proportion using the following equation:

14 $${C}_{{{\rm{mix}}}}={C}_{{{\rm{l}}}}\left(1-X\right)+{C}_{{{\rm{CA}}}}X$$

Where *C*_mix_ is the concentration of a given trace element of the mixed magma, *C*_l_ is the concentration of a given trace element in the l-boninite, *C*_CA_ is the concentration of a given trace element in the calc-alkaline basalt, and *X* is the mass fraction of basalt added, in this case, 0.28.

We then accounted for 45% fractional crystallization of 34% plagioclase, 34% orthopyroxene, 26% clinopyroxene, 3% spinel and 3% titanomagnetite using the Rayleigh fractional crystallization equation following ref. ^95^:

15 $${C}_{{{\rm{melt}}}}={C}_{{{\rm{mix}}}}{F}^{\left(D-1\right)}$$

Where *C*_melt_ is the concentration of a given trace element in the resultant fractionated melt, *F* is the mass fraction of melt remaining, in this case, 0.55, and *D* is the bulk partition coefficient calculated using the partition coefficients listed in Supplementary Data 4. A workflow is provided in Supplementary Fig. 10. All results are listed in Supplementary Data 10.

### Lambda modeling

Lambdas, the coefficients of polynomials fitted to REE patterns, were calculated using the spreadsheet included in ref. ^42^. Lambdas for partial melting of clinopyroxene-poor harzburgite were calculated using the approach outlined in ref. ^96^ using the previously outlined parameters, and is further elaborated in the Supplementary Methods. Results are provided in Supplementary Data 11.

### *T*-*X* pseudosections

We calculated melt isopleths in 2 wt% increments for temperature-composition (*T*-*X*) equilibrium phase diagrams modeling 0 to 3.5 wt.% water addition to the mantle between 1050 °C and 1450 °C. We considered two cases (Fig. 5), which are briefly described below, in the Supplementary Methods, and Supplementary Fig. 11. Elemental inputs for *T*-*X* models are provided in Supplementary Data 6.

The first case involves water addition to a spinel lherzolite at 1.5 GPa, simulating the generation of primary calc-alkaline melts (Fig. 5a). We used the major element composition of the modified calc-alkaline mantle wedge (i.e., primitive mantle composition that underwent 6.5% depletion along a 1400 °C *T*_P_ gradient) as the bulk input for this scenario.

The second case involves water addition to a clinopyroxene-poor spinel harzburgite at 1.7 GPa to simulate the generation of primary boninitic melts (Fig. 5b). The major element composition of the admixed 80 wt.% ultra-refractory harzburgite-20 wt.% ambient upper mantle hybrid source (the unmodified boninite mantle wedge composition) was used as the bulk input. We used the Fractionated P-T software^97^ to calculate melting *P*-*T* (independent of one another) conditions for the primary boninitic magmas over a range of Fe^3+^/ΣFe, assuming Fo_92.9_ for mantle olivine in the source (calculated using our thermodynamic modeling). This provided further constraints on the melting temperature range for the boninites, the results of which are provided in Supplementary Data 12.

## Supplementary information

Supplementary Information

Description of Additional Supplementary Files

Supplementary Data 1

Supplementary Data 2

Supplementary Data 3

Supplementary Data 4

Supplementary Data 5

Supplementary Data 6

Supplementary Data 7

Supplementary Data 8

Supplementary Data 9

Supplementary Data 10

Supplementary Data 11

Supplementary Data 12

Supplementary Data 13

Transparent Peer Review file

## Source data

Source Data

## Data Availability

All data necessary to reproduce our results are available in the paper or its Supplementary Data (also available in the Zenodo public repository 10.5281/zenodo.11558771). Source data are provided with this paper.

## Code availability

The software used for phase equilibrium calculations, MAGEMin^79^, the underlying thermodynamic datasets, and associated scripts to run partial melting calculations are available at no cost from https://github.com/ComputationalThermodynamics. PRIMACALC2^77^ is available at no cost from 10.1002/2014GC005329. The Fractionated P-T spreadsheet^97^ is available at 10.1016/j.epsl.2008.12.020.
