# Supplementary material for: Modern arc-like water content in the source of 3.1-billion-year-old volcanic rocks
Source: Nat Commun. 2026 Jul 7;17:5630. doi: 10.1038/s41467-026-74653-1 (PMC13342610; doi:10.1038/s41467-026-74653-1)
Supplement: Supplementary file 1 — Supplementary Information [file 41467_2026_74653_MOESM1_ESM.pdf]

Supplementary Information for

Modern arc-like water content in the source of 3.1-billion-year-old volcanic rocks

Eric D. Vandenburg* Oliver Nebel, R. Hugh Smithies, Peter A. Cawood, Laura A. Miller, Marc-Alban Millet, and Fabio A. Capitanio

Corresponding author: Eric D. Vandenburg (eric.vandenburg@adelaide.edu.au)

This PDF file includes:

- Supplementary Methods
- Supplementary Figures 1-12
- Supplementary Text
    - The Pilbara Craton, Sholl Terrane and Whundo Group
- Supplementary References

1. Supplementary Methods

The methodologies employed in this study are described in depth below and summarized in flowchart form in Supplementary Figures 10-11.

1.1. Alteration screening and geochemical classification of Whundo Group primitive lavas

Due to the difficulty of identifying the lithologies of Archean volcanic rocks, classifications based on hand sample observations are often inaccurate. As such, we rely on geochemical data to provide a more accurate and less ambiguous lithological classification of our samples. During sampling, all reasonable precautions were taken to obtain the most pristine samples possible. Before classification, we examined the effects of alteration and weathering on the chemical compositions of our samples to ensure that their major and trace elements concentrations are representative of their original magmatic compositions and have not been remobilized (Supplementary Fig. 2). As a first pass, we excluded samples with loss on ignition (LOI) > 5% (approximating the upper limit for magmatic water contents in arcs; ref. 2) from the study, yielding 24 samples from ref. 1 and 16 new samples. Substantial metamorphic and hydrothermal overprint can be ruled out for our samples based on the alteration boxplot[3] method (Supplementary Fig. 2a), as they fall within their respective fields for unaltered modern arc lavas (determined using 2σ values for boninites [*n* = 191], basalts [*n* = 3,930], basaltic andesites [*n* = 5,322], and andesites [*n* = 4,081], compiled from the GeoRoc[4] and EarthChem Portal[5] databases). Likewise, any effects of weathering can be ruled out, as our samples plot along the igneous trend on a MFW (mafic-felsic-weathering) ternary diagram[6] (Supplementary Fig. 2b). Large-ion lithophile elements (LILE; e.g., K, Rb, Sr, Ca, Ba) are valuable tools for evaluating subduction processes such as fluxed melting and slab fluid input[7], but their fluid-mobile nature also makes them heavily susceptible to metamorphic redistribution[8,9]. As such, we evaluated the

degree to which, if any, the LILE systematics have been disturbed. Our samples are well within the range of- and follow a trend similar to those observed within modern primitive arc lavas, as illustrated in diagrams of Rb, Cs, and Ba vs Nb, a highly immobile element during alteration (Supplementary Fig. 2c,e,f). Although our samples have variable LILEs, this variability is commonly observed within modern arcs (e.g., refs. 10-13), even at the scale of a single stratovolcano, and is expected due to heterogeneous and time-variable slab inputs, distinct pulses of magma, and plumbing layouts. Thus, given that the Whundo Group spans approximately 20-30 Myr, variations in LILE concentrations are to be expected. Moreover, Rb shows no correlation with loss-on-ignition (LOI), which is often invoked as a proxy for the degree of alteration, particularly in mafic-ultramafic rocks[1]. Based on these geochemical inferences, the low permeability of the rocks in question (thereby inhibiting fluid-mediated redistribution), lack of evidence for extensive fluid flow, low metamorphic grade (Supplementary Fig. 3), and large volume of material collected, we conclude that the Whundo primitive lavas retain their magmatic LILE concentrations on the whole-rock sample scale, as was previously established by ref. 1.

The major element concentrations of our samples were re-normalized to anhydrous following the scheme of ref. 14. Depending on LOI values, two schemes were used for lithological classification (e.g., basalt). For samples $< 2\%$ LOI, lithology was determined using the modified total alkali-silica (TAS) diagram of ref. 14 (Fig. 2a). Samples with $> 2\%$ LOI were classified using a combination of the same modified TAS diagram and the Zr/Ti versus Nb/Y diagram of ref. 15 (inset Supplementary Fig. 4a), with the latter taking precedence in classification where there were discrepancies. Boninites and low-Ti basalts were classified based on the modified TAS, MgO-TiO_2 (not shown) and the Ti_8-Si_8 (inset Fig. 2a) diagrams of ref. 14 to ensure silica mobility does not influence their identification.

Each sample was assigned to one of the three magmatic series using an iterative approach. First, samples classified lithologically as boninite or low-Ti basalt were

assigned to the boninitic magma group. These samples were divided into the h (h-high trace element abundance) and l (l-low trace element abundance) subsets based on the bifurcation in concentrations observed in extended trace element diagrams (Fig. 3, Supplementary Fig. 6). The remaining samples were examined on a plot of λ_2 (the curvature of the rare-earth element patterns[16]) versus Ti/V; three distinct groupings of basalt are observed (Supplementary Fig. 4b). The first group clusters at Ti/V of 20-30 and λ_2 of ~-15- -5 and plots within the tholeiite field on an AFM ternary diagram (not shown). We assign this group to the tholeiitic series. The second group of basalts forms an array of relatively constant Ti/V of ~40 at increasing λ_2, indicating a source distinct from the tholeiites. As such, we assign these basalts to the calc-alkaline series. Unlike the tholeiitic basalts, the calc-alkaline basalts do not form a cluster in λ_1-λ_2 and Dy/Dy*-Dy/Yb_N-Ti/Ti spaces (Supplementary Fig. 8d), easily distinguishing the two suites. These samples fall within the transitional and calc-alkaline fields on the Th/Yb-Zr/Y magmatic affinity diagram of ref. 17 (Supplementary Fig. 3a). Although some boninitic samples plot within the tholeiitic field of the latter three diagrams, these samples show a closer genetic relationship to the calc-alkaline samples than the tholeiites. The third group of basalts falls along an array in λ_2-Ti/V space between boninites and calc-alkaline basalts; this suggests that this group likely represents the mixing of boninitic and calc-alkaline primitive magmas. As such, we assign these samples to the transitional boninitic-calc-alkaline suite.

The tholeiites (n = 16) comprise basalts that vary in anhydrous SiO_2 and MgO abundances between 49.8-58.8 wt.% and 3.1-8.4 wt.%, respectively, at Mg# (100*[molar Mg/{molar Mg + total molar Fe}]) between 35.3-60.4 (Supplementary Figs. 4-6). Samples of boninitic affinity (h and l, n = 7) have anhydrous SiO_2 and MgO abundances between 50.1-53.1 wt.% and 8.8-10.7 wt.%, respectively, at Mg# between 62.4-67.8. The calc-alkaline basalts (n = 11) are, on average, more primitive than their tholeiitic counterparts, varying in anhydrous SiO_2 and MgO abundances respectively between 51.2-55 wt.% and 3.8-7.9 wt.% at Mg# between 31.6-64.1. Lastly, the transitional boninitic-calc-alkaline suite (n = 6)

comprises lavas that range in anhydrous SiO_2 and MgO abundances between 50.1-56.7 wt.% and 6.9-9.9 wt.%, respectively, at Mg# between 53.6-67.7 and subtle to no TiO_2 depletion relative to other major elements compared to other geochemical groups (Supplementary Figs. 4-6).

1.2. Fractional crystallization corrections

In contrast to boninite sample 180232, which has a high MgO concentration (10 wt.%) and Mg# (66) approaching that of primary melts, the tholeiitic and calc-alkaline basalts are not primary melts (i.e., they do not have MgO/Mg# in equilibrium with mantle olivine[18]). Thus, they require back-correction for fractional crystallization before modelling. To do this, we used the PRIMACALC2 model of ref. 19. We chose the tholeiitic and calc-alkaline samples with the most representative trace element patterns (174474 and 201668, respectively), using parameters listed in Supplementary Data 3 and 9. We assumed isobaric fractional crystallization for both models, using the olivine D^{Ni} model of ref. 20. For the tholeiitic basalts, we assumed a crystallization pressure of 6 kbar (in line with crustal thickness estimates from ref. 21), a water content of 0.75 wt.% (within the range of modern back-arc basalts[22]), and oxygen fugacity (fO_2) of fayalite-magnetite-quartz (FMQ) FMQ-1.5, which overlaps with estimates of the redox state of Archean B-1 type basalts by ref. 23. In contrast, for the calc-alkaline basalts, we assumed a crystallization pressure of 5 kbar (once again in line with crustal thickness estimates from ref. 21), water content of 4 wt.% (within the range of modern arc basalts[24]), and fO_2 of FMQ+0.1, which overlaps with estimates of <3.2 Ga B-2 type basalts from the Pilbara at 4 wt.% H_2O by ref. 23. These parameters provided the most reasonable outputs, including melting temperatures and pressures (Supplementary Data 7).

The back corrections resulted in magmas with 13.4 wt.% MgO for the tholeiitic basalt and 16.8 wt.% MgO for the calc-alkaline basalt. Because olivine was the dominant mineral crystallized, back-correction did not substantially affect the ratios of the trace elements investigated in this study.

1.3. Modified mantle wedge composition modelling

The large diversity in major-trace element compositions between magmatic groups of the primitive Whundo lavas cannot be reconciled with fractional crystallization or crustal contamination (e.g., ref. 1). Instead, the difference between magmatic groups is attributable to a large diversity in mantle source compositions. This diversity can only plausibly be achieved through prior melting events in conjunction with a re-enrichment in selective elements. The section below describes how this is achieved and provides a background methodology for calculating the modified mantle wedge compositions in Figure 4a-c. All parameters are listed with the modelling results in Supplementary Data 3. Modelling was performed using the spreadsheets in Supplementary Data 5.

Heavy rare earth elements (HREEs) can trace partial melting conditions (i.e., melting degree and pressure) due to their moderately incompatible behaviour and low mobility during subduction (e.g., refs. 25, 26). As such, the HREE abundances in Whundo primitive lavas can be used to evaluate melting conditions and degrees of depletion of the mantle sources that give rise to the observed geochemical patterns in our samples.

Partial melting models were constructed using the non-modal pooled fractional melting equation[27,28]:

$$\frac{C_l}{C_0} = \frac{1}{F}\left(1 - \left(1 - \frac{PF}{D}\right)^{\frac{1}{P}}\right) \qquad (1)$$

Where C_l represents the concentration of a given trace element in the melt fraction, C_0 is the concentration of the same trace element in the source; D is the bulk partition coefficient of the starting mineral assemblage for the element in question, P is the bulk reaction coefficient, and F is the degree of melting of the source.

For each element, D is calculated from the sum of individual partition coefficients, $K_{i/l}$ of each mineral i, weighted according to their mass fractions x_i:

$$D = \sum_{i=1}^{n} x_i K_{i/l} \qquad (2)$$

Similarly, P is calculated from the sum of individual partition coefficients weighted according to reaction coefficients p_i:

$$P = \sum_{i=1}^{n} p_i K_{i/l} \qquad (3)$$

Using a similar approach to refs. 29 and 30, we calculated the trace element compositions of the modified mantle wedge (C_{mw}). We first consider two fundamental melting processes involved in producing Whundo Group lava: anhydrous and hydrous non-modal critical fractional melting. These produce the tholeiitic and calc-alkaline magmas, respectively. Here, the mantle has a critical porosity so that there is always trapped melt present. This is achieved by treating trapped melt as a mineral with $K_{\mathrm{trappedmelt/melt}} = 1$ and $p_{\mathrm{trappedmelt}} = 0$ (ref. 31). We consider the mineralogy of the mantle sources of both magmas to be identical: a spinel lherzolite comprised of 59.7% olivine, 15% clinopyroxene, 22.8% orthopyroxene, 0.05% spinel, and 2% trapped melt. The 2% trapped melt comes from variations in Rayleigh wave velocities and S wave delays from seismic imaging of the East Pacific Rise, which suggest 1-2% melt distributed in the upper asthenosphere[32]. We utilize the following equation, rearranged from Eq 1:

$$C_{\mathrm{mw}} = \frac{C_1 F}{1-\left(1-\frac{PF}{D}\right)^{\frac{1}{P}}} \qquad (4)$$

Where C_1 represents the concentration of a given trace element in each sample, D is the bulk partition coefficient of the source, P is the bulk reaction coefficient of the melting modes, and F is the degree of melting of the source.

Because there is no evidence for fluid-fluxed melting in tholeiitic basalts (Supplementary Fig. 8), we propose that they are formed by melting processes resembling those observed in modern mid-ocean ridge basalts (e.g., ref. 33) or back-arc basin basalts. This simulates relatively dry, adiabatic mantle melting. Therefore, for the tholeiites, we assume anhydrous melting in the spinel stability field at ~1.5 GPa using anhydrous partition coefficients for D, with P calculated

following the melting modes -0.149 olivine, 0.949 clinopyroxene, 0.195 orthopyroxene, and 0.005 spinel (ref. 34); and *F* of 11.2%.

The evidence for fluid-fluxed melting in calc-alkaline samples (Fig. 2d, Supplementary Fig. 8) suggests that the primitive parental magmas were formed by hydrous fluid-fluxed melting, similar to what occurs today in the mantle wedge of subduction zones (e.g., ref. 35). As such, we assume hydrous melting in the spinel stability field at ~1.5 GPa using hydrous partition coefficients for *D*, with *P* calculated following the melting modes -0.162 olivine, 0.65 clinopyroxene, 0.508 orthopyroxene, 0.005 spinel[36]; and *F* of 19.2%. The sources for all partition coefficients used in this study are provided in Supplementary Data 4.

Modelling the modified mantle wedge sources of the I-boninites is considerably more difficult due to the unique mineralogies of their modified mantle wedge sources. Boninites are generally regarded to be derived from harzburgitic mantle sources (e.g., refs. 14, 37); thus, the full consumption of clinopyroxene in the source at $F < F_{\mathrm{total}}$, prevents the estimation of the mantle wedge in a single-stage back-calculation. As such, the modified mantle sources of these groups were calculated using a forward modelling approach. We generated a spreadsheet containing a list of possible concentrations in the mantle wedge for each trace element, increasing in intervals of 0.001 ppm. For each possible concentration, the composition of the resultant melt was calculated assuming hydrous, non-modal critical fractional melting using the following set of equations:

$C_{\mathrm{a}} = \frac{C_{\mathrm{mw}}}{F_{\mathrm{out}}}\left(1-\left(1-\frac{P_1 F_{\mathrm{out}}}{D_{\mathrm{hy1}}}\right)^{\frac{1}{P_1}}\right)$ melt composition for melting interval $0 < F \le F_{\mathrm{out}}$, where $F_{\mathrm{out}} = \frac{M_{(\mathrm{lim},F=0)}}{P_1}$ degree of melting required for limiting mineral exhaustion. (5)

$M_{(i,F_{\mathrm{out}})} = \frac{M_{(i,F=0)} - F_{\mathrm{out}}P_1}{1-F_{\mathrm{out}}}$ recalculated mass mode of mineral *i* in source at $F = F_{\mathrm{out}}$. (6)

$C_{F_{\mathrm{out}}} = \frac{C_{\mathrm{mw}}}{1-F_{\mathrm{out}}}\left(\left(1-\frac{P_1 F_{\mathrm{out}}}{D_{\mathrm{hy1}}}\right)^{\frac{1}{P_1}}\right)$ concentration of source at $F = F_{\mathrm{out}}$. (7)

$C_{\mathrm{b}} = \frac{C_{F_{\mathrm{out}}}}{(F_{\mathrm{total}}-F_{\mathrm{out}})}\left(1-\left(1-\frac{P_2(F_{\mathrm{total}}-F_{\mathrm{out}})}{D_{\mathrm{hy2}}}\right)^{\frac{1}{P_2}}\right)$ melt composition for melting interval $F_{\mathrm{out}} < F \leq F_{\mathrm{total}}$. (8)

$C_{\mathrm{l-calc}} = \frac{(F_{\mathrm{out}}C_{\mathrm{a}})+C_{\mathrm{b}}(F_{\mathrm{total}}-F_{\mathrm{out}})}{F_{\mathrm{total}}}$ total melt composition. (9)

Where C_{mw} is the original elemental concentration in the modified mantle wedge, F_{out} is the degree of melting required to exhaust the limiting mineral, D_{hy1} is the bulk partition coefficient for hydrous partial melting of the source before limiting mineral exhaustion, P_1 is the bulk reaction coefficient of the melting reaction before limiting mineral exhaustion, M_{lim} is the initial mass mode of the limiting mineral, $M_{(i,\ F=0)}$ is the initial mass mode of a given mineral in the source, F_{total} is the total degrees of melting, D_{hy2} is the bulk partition coefficient for hydrous partial melting of the source with modes calculated in equation 6 for the second interval of melting, and P_2 is the bulk reaction coefficient of the melting reaction after the limiting mineral exhaustion point. The spreadsheet then finds where $C_{\mathrm{lcalc}} = C_{\mathrm{l}}$ for a given trace element and returns its corresponding C_{mw} value, therefore yielding the elemental composition of the modified mantle source.

To model the depleted harzburgitic source inferred for I-boninitic primitive magmas, we used an initial source mineralogy of 63.406% olivine, 31.164% orthopyroxene, 2.009% clinopyroxene (M_{min}), 1.441% spinel and 2% melt (informed by thermodynamic modelling), where P_1 is the hydrous spinel facies melting modes -0.162 olivine, 0.65 clinopyroxene, 0.508 orthopyroxene, 0.005 spinel[36], P_2 modes are 0.426 olivine, 0.569 orthopyroxene, and 0.005 spinel to maintain the olivine:orthopyroxene ratios observed in ophiolite harzburgites[37], and F_{total} is assumed to be 18%. In this case, C_1 is the trace element

concentration of l-boninite sample 180232. All parameters and results are presented in Supplementary Data 3, partition coefficients are listed in Supplementary Data 4, and the spreadsheets used for calculations are provided in Supplementary Data 5. A summary flowchart is also provided in Supplementary Figure 10.

1.4. Thermodynamic modelling

All thermodynamic modelling was performed using version 1.3.4 of the MAGEMinApp software[38], carried out in the Na_2O-CaO-K_2O-FeO-MgO-Al_2O3-SiO_2-H_2O-TiO_2-Fe_2O_3-Cr_2O_3 (NCKFMASHTOCr) chemical system using the internally-consistent thermodynamic dataset (version ds636/G25) for igneous systems[39]. MAGEMin (Mineral Assemblage Gibbs Energy Minimizer) is the newest generation of Gibbs free energy minimization software and is able to effectively model magmatic systems under suprasolidus conditions. When coupled with the latest thermodynamic dataset and relevant activity-composition models, MAGEMin provides results that exhibit the highest agreement with experimental results of any phase equilibrium modelling software for the melting of the mantle[38-40]. All calculations employed the hybrid solver. For all systems considered, we used the following activity-composition models: silicate melt[39]; garnet, clinopyroxene, orthopyroxene, and ilmenite/hematite[41]; olivine[42]; feldspars[43]; and spinel group minerals[44]; pure phases included were quartz, rutile and sphene. For hydrous systems, we additionally included the following activity-composition models: fluid and biotite[39], and clinoamphibole[45]; H_2O was also considered as a pure phase. Further details are provided in the relevant sections below; all major element inputs are provided in Supplementary Data 6, and raw unformatted outputs are provided in Supplementary Data 13.

1.5. Pre-dripduction mantle wedge composition modelling

The pre-dripduction mantle wedge is the mantle source of the primitive lavas before any dripduction-related input, assuming a two-stage melting process. The HREE budget of the primitive lavas should not be affected by dripduction

processes, so we assumed that the pre-dripduction mantle wedge HREE budget was similar to that of the modified mantle wedge (e.g., refs. 29, 30). Unlike the inverse modelling approach required to calculate the modified mantle wedge compositions, the use of a forward modelling approach in this step enables the incorporation of thermodynamic models that can be used to simulate decompression melting of the mantle, providing phase proportions necessary for trace element modelling and major element compositions of the system, minerals, and melts that can be used to calculate partition coefficients that vary as melting progresses.

For thermodynamic modelling, we use the estimated major element composition of the primitive mantle from ref. 46 as our starting composition. We assumed a $Fe^{3+}/\Sigma Fe$ of 0.019, in line with estimates for the mantle at 3.2 Ga[47]. All calculations are performed assuming an anhydrous system. We first determined the pressure-temperature paths for several mantle potential temperature (T_P) adiabats using the PTX interface set to adiabatic equilibrium melting, with melt deselected, ensuring that the melt extraction threshold had not yet been crossed at the starting pressures. With the paths now determined, we then input the resultant *P*-*T* points back into MAGEMin and run the program in fractional melting mode with melt re-enabled as a phase and the melt extraction threshold determined based on the pressure at which the path crosses the solidus. This simulates fractional melting along the prescribed T_P adiabat.

Using the resultant outputs, we determine the incompatible trace-element compositions of the pre-dripduction mantle wedge (C_{res}) and the degree of its melt depletion, assuming a starting point of a primitive mantle composition (C_0; ref. 48). We use a stepwise approach that replicates critical fractional melting using the following set of equations:

$M_i^{\mathrm{res}} = M_{i-1}^{\mathrm{res}} - \Delta \mathrm{E} C_{\mathrm{l},i}$ the mass of a given element in the residue at step *i*. (10)

$C_{\mathrm{l},i} = \frac{M_{i-1}^{\mathrm{res}}}{S_i D_i + \phi_{\mathrm{bef},i}}$ the concentration of the element in the melt in equilibrium with the solids at step *i*. (11)

$C_{\mathrm{solid},i} = C_{\mathrm{l},i} D_i$ the concentration of the element in the solids at step *i*. (12)

$C_{\mathrm{res},i} = \frac{M_i^{\mathrm{res}}}{1 - E_i}$ the concentration of the element in the residue (trapped melt + solids) at step *i*. (13)

Where $M_0^{\mathrm{res}} = C_0$, E_i is the fraction of cumulative extracted melt at step *i*, $\Delta \mathrm{E}_i = E_i - E_{i-1}$ is the instantaneous amount of melt extracted between steps, $S_i = 1 - F_i$ is the solid mass fraction at step *i*, F_i is the total amount of melt at step *i*. $\phi_{\mathrm{bef},i} = \phi_i + \Delta \mathrm{E}_i$ is the melt present just before extraction at the end of step *i*, and ϕ_i is the fraction of retained melt after extraction at step *i*. Note that in this instance, D_i is the bulk partition coefficient of the solid assemblage at step *i*, and hence, unlike with the modified wedge calculations, melt is not included. The individual partition coefficients ($K_{i/l}$) of each mineral were calculated for each step using the equations/methods listed in Supplementary Data 4.

Here, the degree of melt depletion (*F*) in generating the pre-dripduction mantle wedges is obtained when the HREE concentrations modelled for the pre-dripduction mantle wedges approximately equal those of the modified mantle wedges. This is achieved by minimizing the root mean square of the log ratios of HREE abundances in the unmodified and modified wedge compositions.

Because MAGEMin only allows for a melt volume threshold to be applied during fractional melting, we approximated the 2 wt.% melt threshold used above for the modified mantle wedge calculations by applying a 1.7 vol.% melt extraction threshold to calculate the tholeiitic and calc-alkaline lavas. Here, the residues produced by fractional melting along a prescribed 1,400°C T_P adiabat yielded the best fits for the pre-dripduction mantle wedges, where the mantle adiabat crosses the solidus at around 2.42 GPa. The best fit for the unmodified tholeiitic mantle

wedge was achieved at an F of 7.2%, while the unmodified calc-alkaline mantle wedge required slightly less depletion, at an F of 6.5%.

The unmodified mantle source of the boninites requires a mixture of two components to reproduce its unique HREE pattern: a predominant ultra-refractory harzburgite (URH) component with a subordinate slightly-depleted lherzolite component.

The refractory harzburgite component is produced along a prescribed 1,605°C T_P adiabat, where melting begins at ~6.1 GPa with the primitive mantle source undergoing an F of 36.2%. This mantle potential temperature approaches the melting conditions of komatiite generation (e.g., refs. 49, 50), albeit slightly lower, but this is to accommodate limitations on calibrated pressures imposed by the thermodynamic dataset. We chose a critical porosity of 3.5 vol%, which is higher than that for the tholeiitic and calc-alkaline models (1.7 vol%), for two reasons. First, there remains substantial debate surrounding the melting style of komatiite generation, notably whether they were produced by fractional vs. batch melting[49-51]; as a compromise, we chose critical fractional melting with a higher melt extraction threshold. Second, because the timescale of melt extraction scales as a reciprocal of $\Delta\rho^2$, the density contrast between melt and residual crystals (e.g., ref. 52) along the 1,605°C T_p adiabat for a fertile garnet peridotite is higher than for a fertile spinel lherzolite along the 1,400°C T_p adiabat, as melting begins at a higher pressure. However, a lower critical porosity would increase the depletion rate for incompatible elements (e.g., ref. 28), requiring a greater dripduction contribution to these elements, potentially at a lower degree of prior melt extraction. A more depleted unmodified mantle wedge would also require a higher fluid flux to melt at a given temperature, thus reinforcing our results.

The resultant trace element composition of the refractory harzburgite was mixed with 20% ambient upper mantle (an average of the trace element compositions of the unmodified tholeiitic and calc-alkaline mantle wedges). This combination of T_P, depletion and mixing proportion parameters yielded the best fit to the

modified boninitic mantle wedge. Neither component on its own can suitably reproduce the HREE (+ Y) slope; URH is too steep (upward), whereas slightly-depleted lherzolite is too shallow (flat to slightly downward). Likewise, using a lower T_P for the harzburgite component produces a poorer fit, even after adjusting the component proportions to account for this.

Major element inputs for the thermodynamic depletion modelling are provided in Supplementary Data 6, major element results are provided in Supplementary Data 7, and trace element results are provided in Supplementary Data 8. A workflow is provided in Supplementary Figure 10, and details are provided in Supplementary Data 3.

1.6. Magma mixing and fractional crystallization

To produce the patterns observed in the transitional boninitic-calc-alkaline basalts, we mixed average l-boninite with 28% average calc-alkaline basalt using the following equation:

$$C_{\mathrm{mix}} = C_{\mathrm{l}}(1 - X) + C_{\mathrm{CA}}X \qquad (14)$$

Where C_{mix} is the concentration of a given trace element of the mixed magma, C_l is the concentration of a given trace element in the average l-boninite, C_{CA} is the concentration of a given trace element in the average calc-alkaline basalt, and X is the mass fraction of basalt added, in this case, 0.28.

We then accounted for 45% fractional crystallization of 34% plagioclase, 34% orthopyroxene, 26% clinopyroxene, 3% spinel and 3% titanomagnetite using the Rayleigh fractional crystallization equation[28,53]:

$$C_{\mathrm{melt}} = C_{\mathrm{mix}}F^{(D-1)} \qquad (15)$$

Where C_{melt} is the concentration of a given trace element in the resultant fractionated melt, F is the mass fraction of melt remaining, in this case, 0.55, and D is the bulk partition coefficient calculated using the partition coefficients listed in Supplementary Data 4. All results are listed in Supplementary Data 10.

1.7. *T-X* pseudosections

We calculated melt isopleths in 2 wt.% increments for temperature-composition (*T*-*X*) equilibrium phase diagrams modelling 0 to 3.5 wt.% water addition to the mantle between 1,050°C and 1,450°C. We considered two cases (Fig. 5), which are briefly described below and in Supplementary Figure 10. Elemental inputs for *T*-*X* models are provided in Supplementary Data 6.

The first case involves water addition to a spinel lherzolite at 1.5 GPa to simulate the generation of primary calc-alkaline melts (Fig. 5a). We used the major element composition of the modified calc-alkaline mantle wedge (i.e., primitive mantle composition that underwent 6.5% depletion along a 1,400°C T_P gradient) as the bulk input for this scenario.

The second case involves water addition to a clinopyroxene-poor spinel harzburgite at 1.70 GPa to simulate the generation of primary boninitic melts (Fig. 5b). The major element composition of the admixed 80% ultra-refractory harzburgite (URH) -20% ambient upper mantle (AUM) hybrid source (the unmodified boninite mantle wedge composition) was used as the bulk input. As described below, we used the fractionated P-T software[54] to calculate melting *P*-*T* conditions for the primary boninitic magmas over a range of $Fe^{3+}/\Sigma Fe$.

1.7.1. P-T conditions of primary boninitic melts

Because the Whundo boninitic lavas have MgO contents >8 wt.%, their compositions have avoided the most significant effects of fractional crystallization. Therefore, their compositions can be input into the Fractionated P-T software[54] to calculate melting *P*-*T* conditions for the primary boninitic magmas; this data is then used to constrain the water content of the boninite mantle source (Fig. 5b, Supplementary Figs. 9b, 11). The pressure and temperature estimates produced by this program are independent of each other. For each boninite sample, we calculated the *P*-*T* conditions of melting for a melt with 2, 3, and 5 wt.% H_2O (the range of H_2O in boninites from ref. 37), at molar $Fe^{3+}/\Sigma Fe$ of 0.11,

0.16, and 0.21 (0.21 being the $Fe^{3+}/\Sigma Fe$ of modern boninites; refs. 37, 55). We used the olivine Fo content $Fo_{92.9}$, determined in MAGEMin using the major element composition of the modelled boninitic mantle source. The results of these calculations are provided in Supplementary Data 12. For 180232, this yielded a temperature between 1,317°C and 1,439°C, at pressures between 1.55 and 1.94 GPa. For Figure 5b and Supplementary Figure 9b, we used a pressure of 1.70 GPa, which is in the middle of these estimates.

1.8. Lambda melting curves

Construction of the melting curves in λ_1-λ_2 space[16] for clinopyroxene-poor spinel harzburgite in Figure 2c consists of two components. The first component, the melting curve itself, was determined via forward critical fractional melting of the modified mantle source of the l-boninite sample 180232 using the parameters stated above in Section 1.3. For each REE, it follows that:

For the interval of melting from $0 < F \leq F_{out}$, where $F_{\mathrm{out}} = \frac{M_{(\mathrm{lim}, F=0)}}{P_1}$, the degree of melting required for clinopyroxene exhaustion, the ratio of the concentration of an element in the melt to its concentration in the original source (C_l/C_0) is given by:

$$\frac{C_{\mathrm{l}}}{C_0} = \frac{C_{\mathrm{a}}}{C_{\mathrm{mw}}} = \frac{1}{F}\left(1 - \left(1 - \frac{P_1 F}{D_{\mathrm{hy1}}}\right)^{\frac{1}{P_1}}\right) \qquad (5)$$

For melting past F_{out}, C_l/C_0 is given by:

$$\frac{C_{\mathrm{l}}}{C_0} = \left(\frac{F_{\mathrm{out}}}{F}\left(\frac{1}{F_{\mathrm{out}}}\left(1 - \left(1 - \frac{P_1 F_{\mathrm{out}}}{D_{\mathrm{hy1}}}\right)^{\frac{1}{P_1}}\right)\right)\right) + \left(1 - \frac{F_{\mathrm{out}}}{F}\left(\frac{\frac{C_{F_{\mathrm{out}}}}{C_{\mathrm{mw}}}}{(F_{\mathrm{total}} - F_{\mathrm{out}})}\left(1 - \left(1 - \frac{P_2 (F_{\mathrm{total}} - F_{\mathrm{out}})}{D_{\mathrm{hy2}}}\right)^{\frac{1}{P_2}}\right)\right)\right)$$

(11), where $\frac{C_{F_{\mathrm{out}}}}{C_{\mathrm{mw}}} = \frac{1}{1 - F_{\mathrm{out}}}\left(\left(1 - \frac{P_1 F_{\mathrm{out}}}{D_{\mathrm{hy1}}}\right)^{\frac{1}{P_1}}\right)$ is the residue/original source ratio at $F = F_{out}$. (modified Eq. 7)

Finally, the concentration of each REE is determined by multiplying C_l/C_0 by the concentration in the modified mantle source of 180232. This creates the melting curve for 180232, anchored at its mantle source (Supplementary Data 11).

The lambdas also function in this case as petrogenetic process vectors (e.g., refs. 16, 57) describing the change in average REE slope and curvature between the REE patterns of the source and melt; holding source mineralogy and melt reactions constant, varying the source's λ_1-λ_2 values will not change the shape of the melting curve, merely changing the anchor point of the curve to the new source's λ_1-λ_2 values[16]. Therefore, the same melting curve for the modified mantle source can be used to constrain the proportion and amount of depletion of the URH component in the first-stage mantle source that could account for the concave-up REE patterns in the boninites. Except for Dy, Er, Yb, and Lu, the re-enrichment step dominates the budget of all other REEs considered here and is therefore largely independent of these URH parameters. Thus, if we hold the amount of re-enrichment constant, the offsets in λ_1-λ_2 between sources that contained variable amounts of URH component produced by variable degrees of depletion should be the same for both the unmodified and modified mantle wedge sources, simply requiring translation from the λ_1-λ_2 value of the unmodified mantle source to the corresponding λ_1-λ_2 of the modified mantle source. This component is calculated using the same methods as for the pre-dripduction mantle wedge (Section 1.4.). For example, in Fig. 4b, we varied the proportion of the URH component between 70 and 82 wt.% and the degree of melting to produce URH between 33.5-36.5%.

The λ_1 and λ_2 values for the modified mantle source of each lithology can then be determined by calculating the offset between the unmodified mantle source of 180232 and the source in question and then applying this offset to the modified mantle source of 180232. To create enrichment trends, the enrichment factors for REE in 180232 are scaled to different percentages of enrichment on La, thereby maintaining REE patterns and element ratios. In Fig. 4b, we scaled the REEs to

between 86.25 and 92.50% re-enrichment contribution on La (180232 is 86.57%) and translated the points accordingly.

The curves for melting of modified hybrid mantle sources containing a variable proportion of the URH component, produced by variable degrees of depletion, were created using the same melting reactions as discussed above in Section 1.3. for the generation of boninitic magmas, with the phase proportions calculated in MAGEMin at 1.70 GPa and 1,075°C using the major element compositions of the variable URH-ambient upper mantle admixtures.

1.9. Alternative boninite model

In addition to the main model of boninite generation presented in Figures 4c and 5b, we present an alternative model in Supplementary Figure 8 to illustrate the viability of alternative petrogeneses for the Whundo boninites. Below, we describe the alternative model and its differences with the main model. This is also summarized in Supplementary Data 3.

1.9.1. Trace element modelling (Supplementary Figure 8a)

The modified mantle wedge modelling for the alternative boninite model was conducted using the same equations and melting reactions as the main boninite model. However, we used a slightly different mineralogy (61.152% olivine, 2.74% clinopyroxene, 32.438% orthopyroxene, 1.666% spinel and 2% retained melt; informed by thermodynamic modelling), on account of the different chemical composition of the mantle source informed by the mantle depletion modelling. We also use a slightly lower degree of melting to generate the boninites (17.5%), as this minimizes the root mean square of the log ratios of HREE abundances in the unmodified and modified wedge compositions.

The pre-dripduction mantle wedge composition modelling in the alternative model uses the same starting composition and melt connectivity threshold as the main model. Unlike the main boninite model, where we consider a melt-bearing harzburgitic residue produced along a 1,605°C T_P adiabat, for the alternative

model, we calculated the trace element composition for melt-free, ultra-refractory solid harzburgitic residue produced along a 1,610°C T_P adiabat. Here, melting begins at ~6.3 GPa with the primitive mantle source undergoing an *F* of 34.2%.

Unlike the main boninite model, which considers admixing of ambient upper mantle to a melt-bearing harzburgite residue to produce a hybrid mantle source, the alternative boninite model considers metasomatism of the melt-free, ultra-refractory harzburgitic solids by a small amount of primary tholeiitic melt. Here, we added 3.9 wt.% of the primary tholeiitic melt (the fractional crystallization-corrected composition of 174474) to the trace element composition of the refractory harzburgitic solid. This combination of T_P, depletion and melt addition parameters yielded the best fit to the modified boninitic mantle wedge.

1.9.2. T-X pseudosection (Supplementary Figure 8b)

The alternative boninite model *T-X* pseudosection uses all but one of the same parameters as those of the main boninite model; it also involves water addition to a clinopyroxene-poor spinel harzburgite at 1.7 GPa. Here, we used the major element composition of a mixture of 3.9 wt.% primary tholeiitic melt with 96.1 wt. ultra-refractory harzburgitic solids (URH)-20% ambient upper mantle (AUM) hybrid source (the unmodified boninite mantle wedge composition) as the bulk input composition. We used the same constraints imposed by the fractionated P-T software as for the main model.

1.10. Modelling uncertainties

We refer the reader to refs. 38, 39, and 42 for discussions related to the general uncertainties associated with the modelling software and its underlying thermodynamic datasets. Below, we discuss the uncertainties that are directly resultant from our modelling approach.

The largest source of error in our modelling approach is associated with the second-stage melting step (i.e., the melting event that produces the tholeiitic, calc-alkaline and boninitic magmas from their respective mantle sources) used to

calculate the modified mantle sources. While some amount of uncertainty may arise due to i) the limited number of studies on partition coefficients and melting reactions during hydrous melting of the mantle; ii) static partition coefficients and melting reactions; and iii) having to choose a degree of melting for each sample used for modelling, we have made our best efforts to choose appropriate degrees of melting, partition coefficients and melting reactions based on previous studies. An additional constraint on the degree of melting is that the HREE concentrations of the magmas' mantle sources must be lower than the primitive mantle, given the assumptions of HREE immobility and mantle depletion discussed above. Thermodynamic phase equilibrium modelling software cannot currently perform inverse melting modelling, such that there is no straightforward way to determine variable partition coefficients and melting reactions. However, the continued use of partial melting equations, more than 50 years after their initial development[27,28,56], is a testament to their robustness and suitability for this study.

Although thermodynamic modelling software are currently unable to perform inverse modelling of mantle sources, they can be used for forward modelling of mantle melting. Our approach for calculating the trace element compositions of the unmodified mantle sources, employing thermodynamic modelling of melting along a mantle potential temperature adiabat to inform the phase relationships and calculate variable mineral-melt partition coefficients used as inputs in the partial melting equations, provides a much more realistic method of simulating mantle depletion than can be done with static melting reactions and partition coefficients.

The software and underlying a-x models and datasets used here have been shown to have the greatest amount of agreement of any thermodynamic modelling method with the results of experimental petrology studies for melting of the upper mantle[40]. The igneous thermodynamic dataset ds636/G25 is applicable to pressures of up to 7 GPa and has been shown to largely retain its fidelity to the

experimental results of mantle melting at 6 GPa[58]. Therefore, it is suitable for use in our stage 1 mantle depletion step, which involves pressures of up to 6.3 GPa.

Finally, several considerations must be taken into account with respect to the hydrous mantle melting modelling employed in Figure 5 and Supplementary Figure 8b. Firstly, unlike other thermodynamic models (i.e., pHMELTS[59]; note alphaMELTS is not used because it cannot create *T*-*X* pseudosections), the ds636/G25 x-eos cannot store water in nominally anhydrous minerals. As discussed in the main text, H_2O storage in nominally anhydrous minerals can be on the order of several hundred ppm[60,61], so the mantle H_2O contents presented here should be considered minimum estimates. Second, modelling of hydrous melting in the ds636/G25 x-eos applies to pressures of up to 2 GPa; our use cases are well within this range. Third, there remains substantial uncertainty regarding the position of the H_2O-saturated solidus in *P*-*T*-*X* space as highlighted by experimental studies, even on the scale of a single bulk composition (i.e., refs. 60, 61 vs. 62, 63; 58); the position of the H_2O-saturated solidus for HZ1 in P-T space calculated using MAGEMin with the ds636/G25 x-eos falls within this uncertainty (Supplementary Fig. 11) and is reasonably in agreement with experimental studies at the pressures of interest.

2. Supplementary Figures

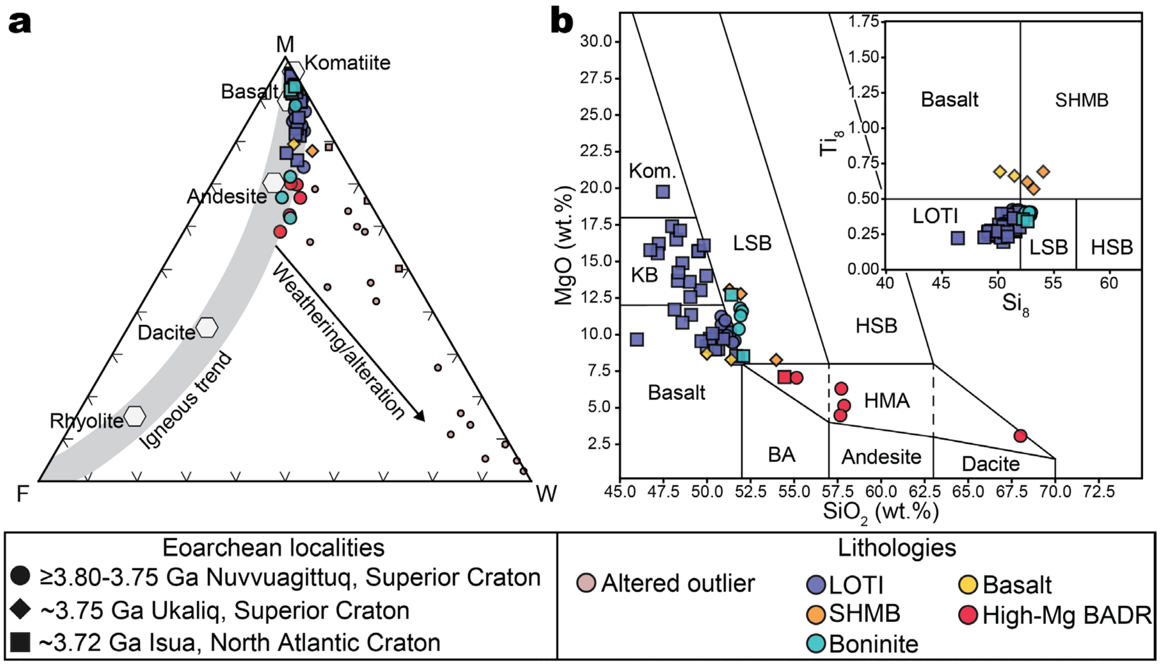

Supplementary Figure 1. **Minimal existence of Eoarchean boninites.** Most samples referred to in the literature as “boninite” from locales older than the Whundo Group (3.12 Ga) are either not true boninites or are too altered to determine their affinity conclusively; the remaining truly boninitic samples are restricted in number, plot only marginally within the low-Si boninite (LSB) field, and deviate from the typical evolutionary path of boninitic suites (see ref. 14). (a) MFW (mafic-felsic-weathering) ternary diagram (6) illustrating that many samples described as “boninitic” have experienced significant weathering and/or alteration and thus cannot be conclusively identified as boninites. (b) the remaining non-cumulate samples, plotted on the MgO-SiO_2 and Ti_8-Si_8 boninite classification scheme diagrams[14] are, for the most part, not true boninites. Although all of the samples described as “boninitic” from the Ukaliq Supracrustal Belt[64,65] are basalts and siliceous high-Mg basalts (SHMB), a few true low-Si boninites exist from the middle unit of the nearby, coeval Nuvvuagittuq Greenstone Belt (*n* = 5; ref. 66). However, these are restricted to a single, <200 m-thick package and are subordinate to low-Ti basalts (LOTI). Similarly, most samples described as

"boninitic" from the Garbenschiefer unit of the Isua Supracrustal Belt[67-71] are LOTI, except for two samples. Abbreviations: BADR – basalt-andesite-dacite-rhyolite, HMA – high-Mg andesite, HSB – high-Si boninite, KB – komatiitic basalt.

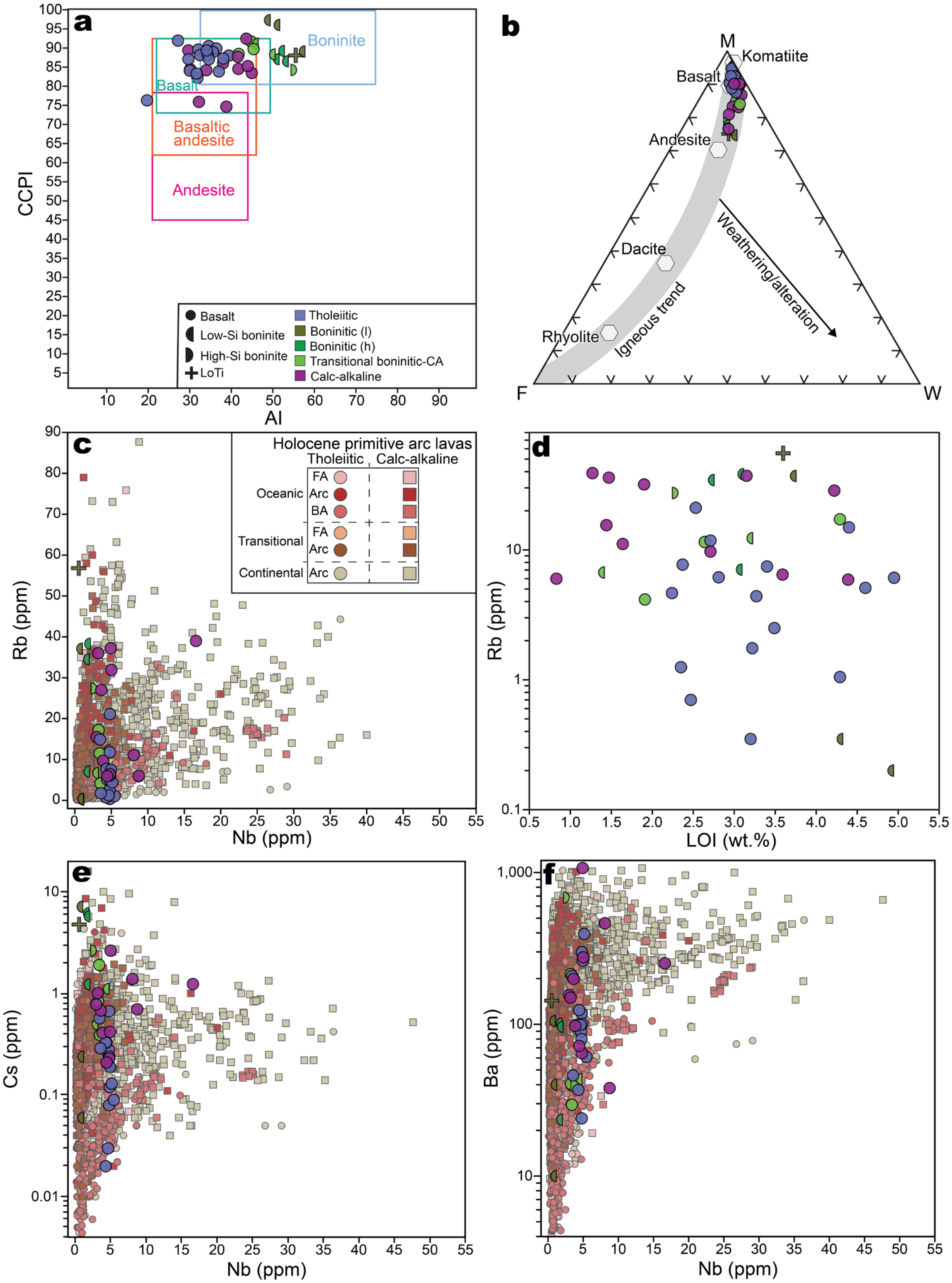

Supplementary Figure 2. **Whundo Group primitive lavas retain their primary magmatic compositions.** (a) chlorite-carbonate-pyrite index (CCPI;

100[FeO+MgO]/[FeO+MgO+Na_2O+K_2O]) vs. Ishikawa alteration index (AI; 100[MgO+K_2O]/[MgO+K_2O+CaO+Na_2O]) diagram (after ref. 3). Except for three tholeiitic samples, the Whundo primitive lavas plot within their respective fields for unaltered modern arc lavas ((determined using 2σ values for boninites [*n* = 191], basalts [*n* = 3,930], basaltic andesites [*n* = 5,322], and andesites [*n* = 4,081], compiled from the GeoRoc[4] and EarthChem Portal[5] databases). Therefore, metamorphic redistribution of elements is insignificant. (b) MFW (mafic-felsic-weathering) ternary diagram[6] illustrating that the primitive lavas plot within the unweathered igneous array and have not experienced weathering-related alteration. (c) The Rb-Nb systematics of the primitive lavas are well within range of those observed in unaltered modern arc lavas spanning the MgO content observed in Whundo primitive lavas (data compiled from GeoRoc[4] and EarthChem Portal[5] databases). (d) Rb shows no correlation with loss-on-ignition (LOI). The Cs (e) and Ba (f) -Nb systematics of the primitive lavas are also well within range of those observed in unaltered modern arc lavas. Taken together, (c-f) demonstrate that the primitive lavas of the Whundo Group retain their magmatic large-ion lithophile (LILE) concentrations and can therefore be used to evaluate petrogenesis.

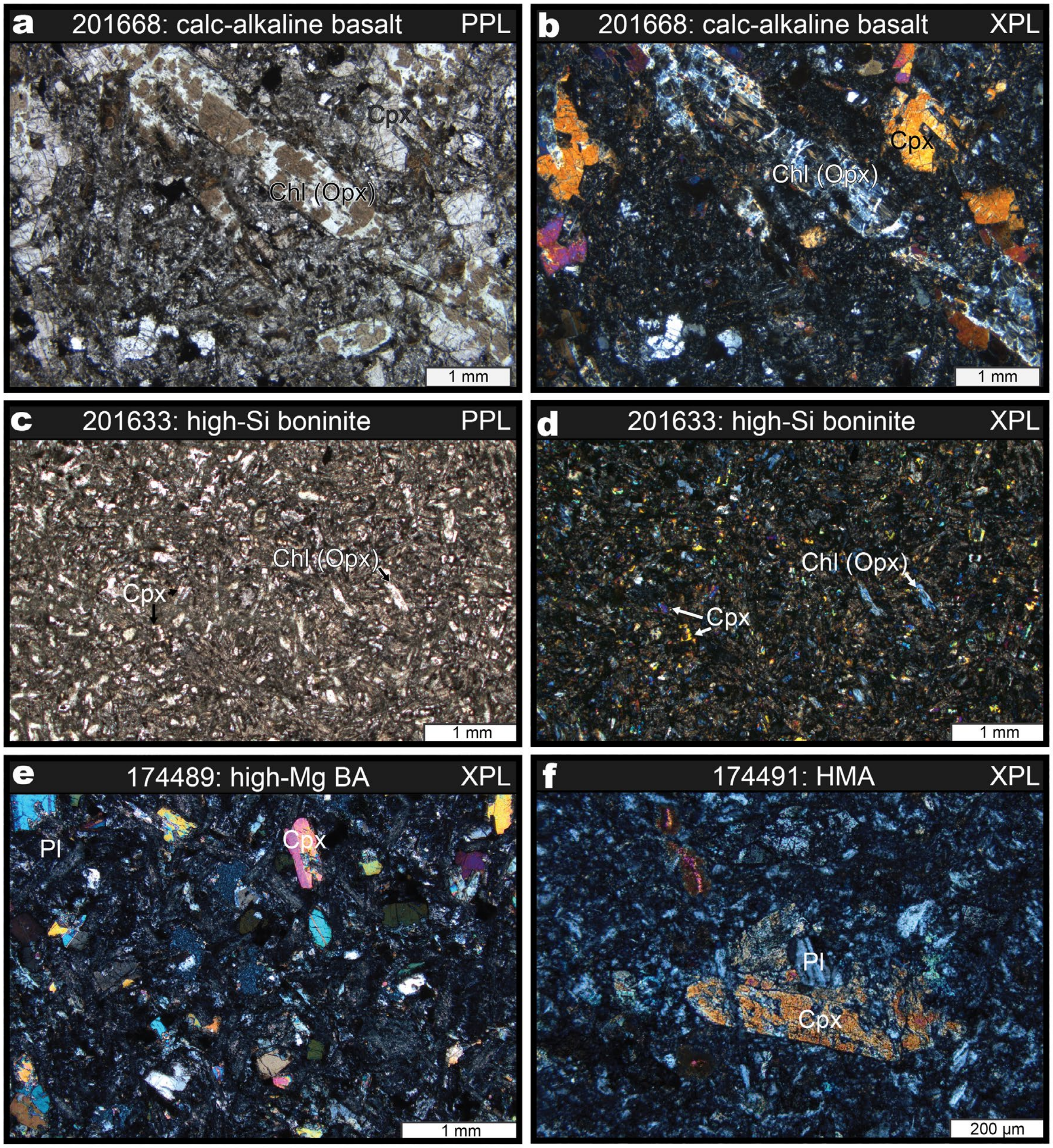

Supplementary Figure 3. **Petrographic evidence for preserved igneous textures in Whundo Group lavas.** Plane-polarized (PPL; a) and cross-polarized (XPL; b) transmitted light photomicrographs of calc-alkaline basalt sample 201668 displaying medium-grained acicular orthopyroxene (now altered to chlorite) and subhedral clinopyroxene phenocrysts. PPL (c) and XPL (d) photomicrographs of high-Si boninite sample 201633, consisting of fine-grained acicular orthopyroxene set in a groundmass of microcrystalline to very fine-grained clinopyroxene microlites and glass; this is very similar to modern high-Si boninites (e.g., ref. 72). XPL (e) photomicrograph of intergranular-textured high-

Mg basaltic andesite (HMBA) sample 174489, consisting of fine-grained subhedral to euhedral clinopyroxene and fine-grained sericite-dusted plagioclase laths. XPL (f) photomicrograph of high-Mg andesite (HMA) sample 174491, displaying very fine-grained plagioclase microphenocrysts set in a groundmass of plagioclase microlites. Sample 174489 and 174491 are from ref. 1.

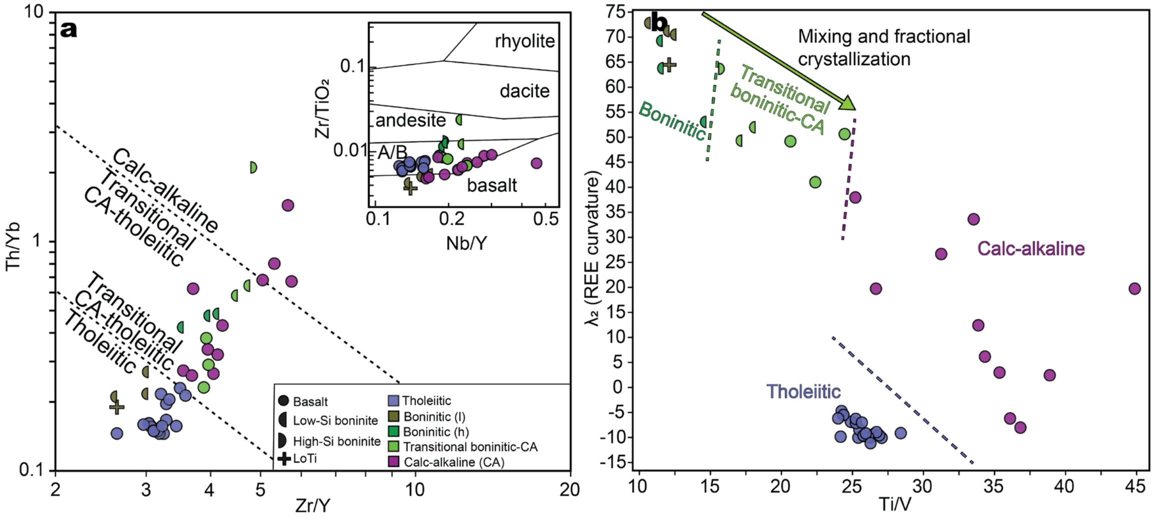

Supplementary Figure 4. **Classification scheme for Whundo Group lavas.** (a) Th/Yb-Zr/Y magmatic affinity diagram[17] used to distinguish calc-alkaline and tholeiitic samples. Inset: Zr/Ti-Nb/Y diagram[15] used to classify non-boninitic samples with greater than 2% loss on ignition (LOI). (b) λ_2-Ti/V diagram distinguishes hybrid basalts and boninites. Refer to the methods for further details. Abbreviations: LoTi – low-Ti basalt.

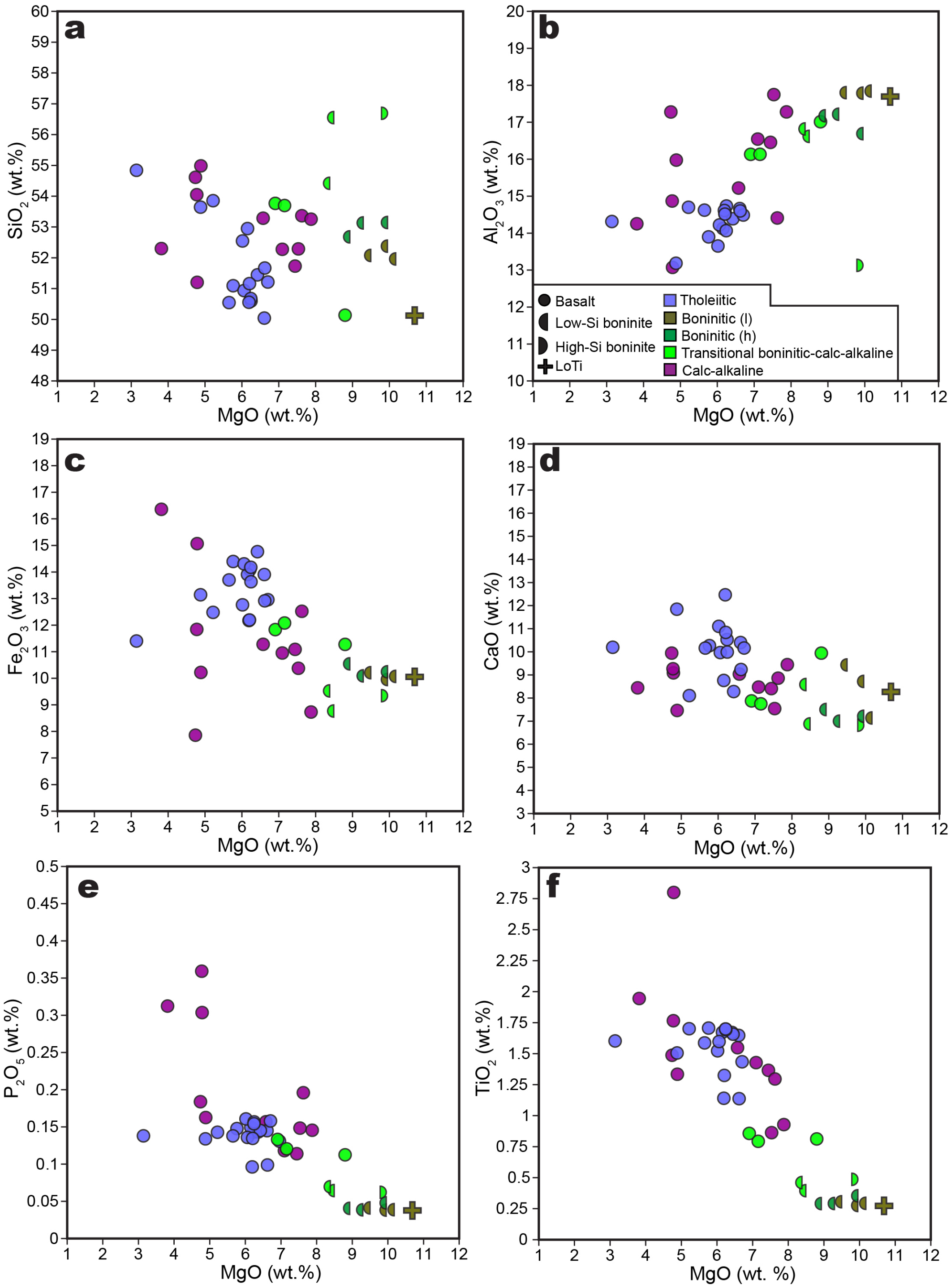

Supplementary Figure 5. **Major element compositions of Whundo Group lavas with MgO as abscissa.** (a) SiO_2, (b) Al_2O_3, (c) total Fe as Fe_2O_3, (d) CaO, (e) P_2O_5, (f) TiO_2.

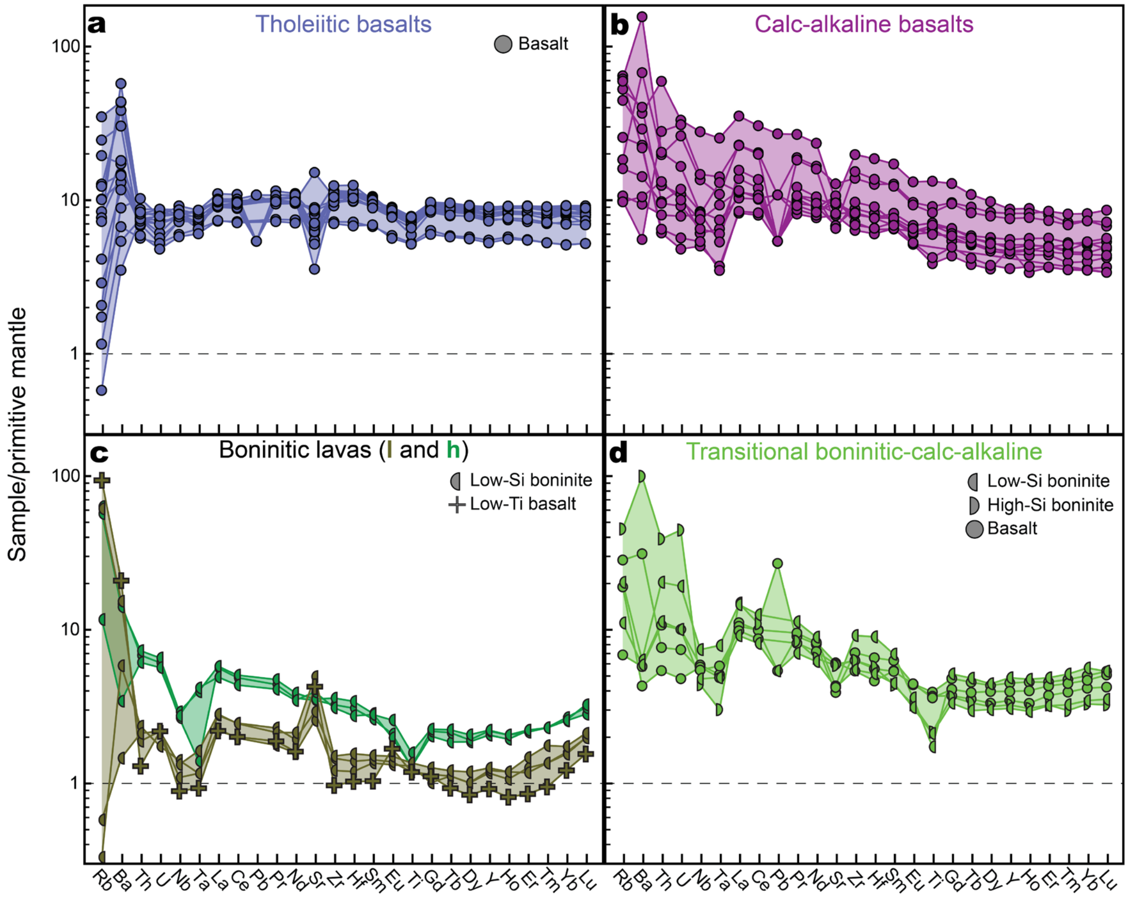

Supplementary Figure 6. **Trace element patterns of primitive Whundo Group lavas**. Primitive mantle-normalized[48] trace element patterns of individual samples comprising tholeiitic basalts (*n* = 16; a), calc-alkaline basalts (*n* = 11; b), boninites (*n* = 7; c), and transitional boninite-calc-alkaline lavas (*n* = 6; d). The shaded fields indicate the range of each magmatic series.

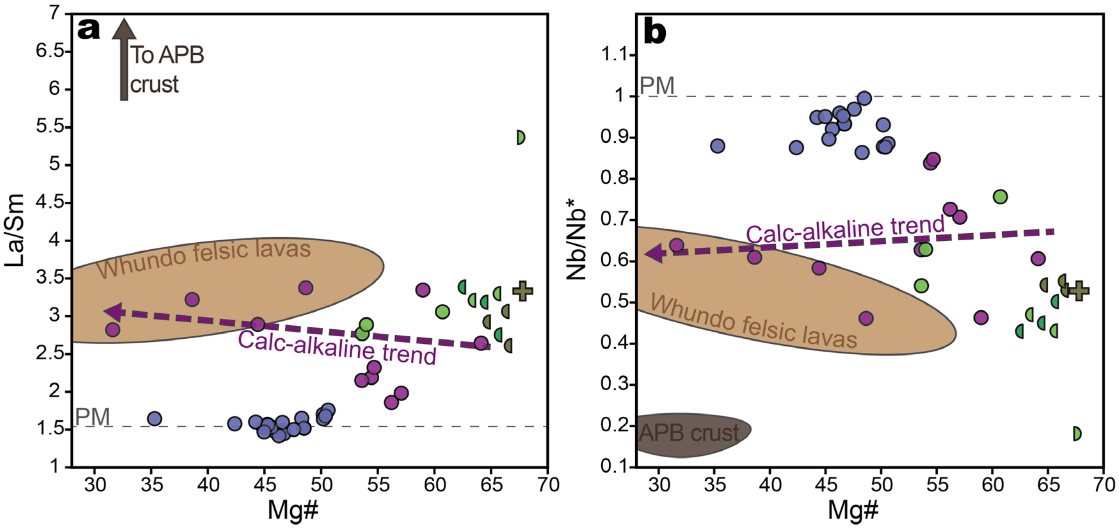

Supplementary Figure 7. **Contamination of primitive lavas by felsic crust is irreconcilable with differentiation trends.** Differentiation of magmatic series towards lower Mg# is not accompanied by a sharp, systematic increase in La/Sm (a) or decrease in Nb/Nb* ($Nb_{PM}/\sqrt{[Th_{PM} \times La_{PM}]}$; PM denoting normalization to primitive mantle; ref. 48) for the boninitic, calc-alkaline or hybrid lavas, but rather remains consistently variable. Given the difference in Nb/Nb* of ~0.3 between the tholeiitic basalts and felsic lavas, significant crustal contamination by the felsic lavas would be detectable if this explained the origin of the calc-alkaline basalts. However, the lack of arrays between the tholeiitic and calc-alkaline basalts in these contamination-sensitive proxies points to the contrary. In fact, the variability in Nb/Nb* and La/Sm with Mg# observed in the calc-alkaline basalts is more consistent with source enrichment heterogeneity. >3.18 Ga Ancestral Pilbara Block (APB) crust and Whundo felsic lava fields are calculated from refs. 73 and 1, respectively. Symbols as for Supplementary Figs. 4-6.

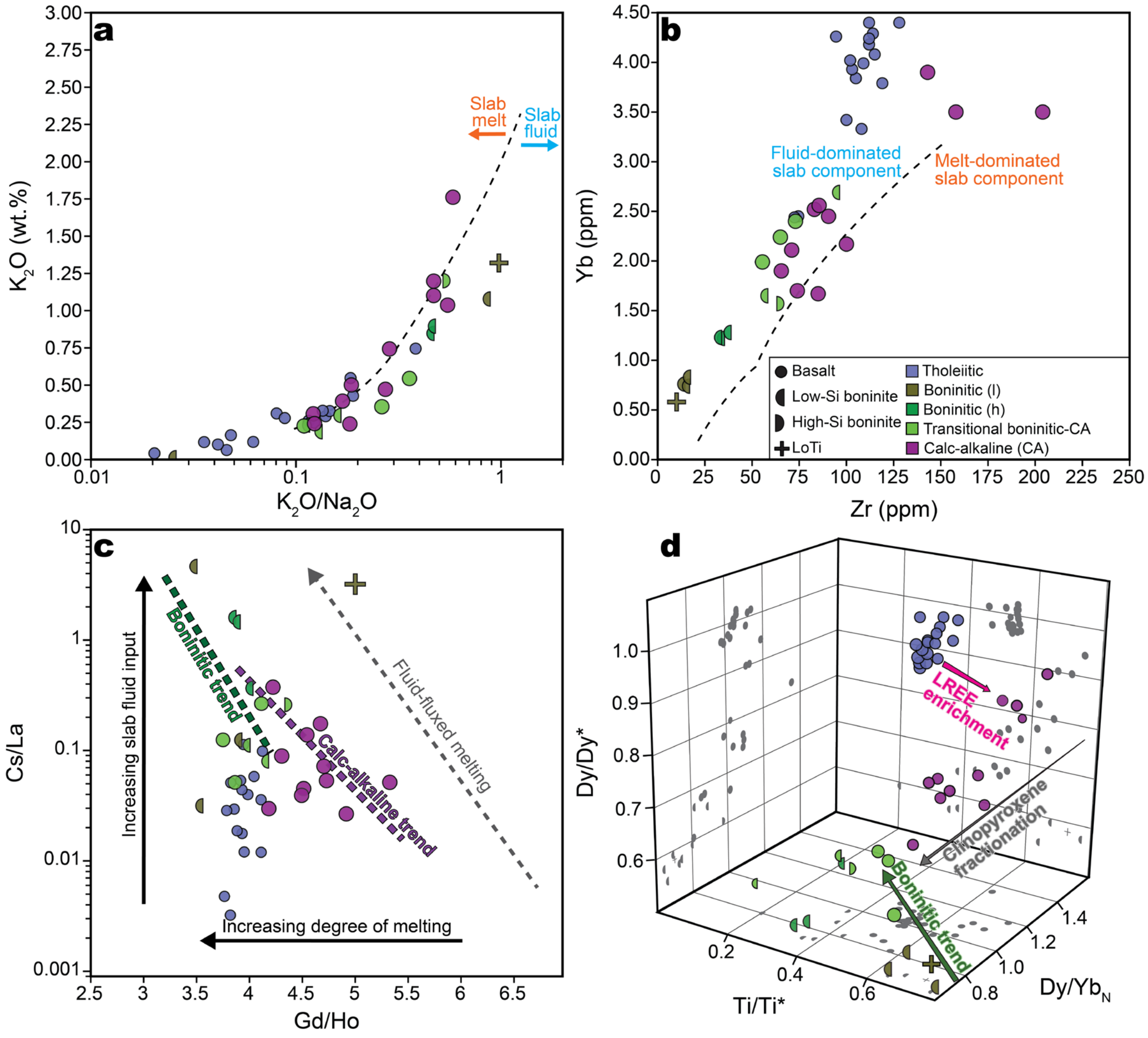

Supplementary Figure 8**. Petrogenetic processes evidenced by Whundo primitive lavas.** Plots of K_2O vs K_2O/Na_2O (a) and Yb vs Zr (b) after ref. 18, differentiating between melt- vs fluid-dominated slab inputs. The high K_2O/Na_2O and low Zr of the boninites and most calc-alkaline basalts indicate that slab fluids comprise the dominant slab component added to the source of these lavas. The negative covariations observed in Cs/La-Gd/Ho (c) space for the boninitic and calc-alkaline samples are consistent with fluid-fluxed melting. On the other hand, the tholeiitic samples do not exhibit these covariations, suggesting that flux melting did not play a significant role in their petrogenesis. Dy/Dy* (dysprosium anomaly: $Dy_N/[La_N^{4/13} + Yb_N^{9/13}]$; ref. 74)-Dy/$Yb_N$-Ti/Ti* (titanium anomaly: $Ti_N/[0.5*\{Sm_N+Gd_N\}+\{Tb_N/2\}]$) (d) systematics of primitive lavas with fractionation

and enrichment vectors. The green arrow illustrates the trend for boninitic-calc-alkaline hybrid samples originating from I-boninites. Symbols as for Supplementary Figs. 4-7.

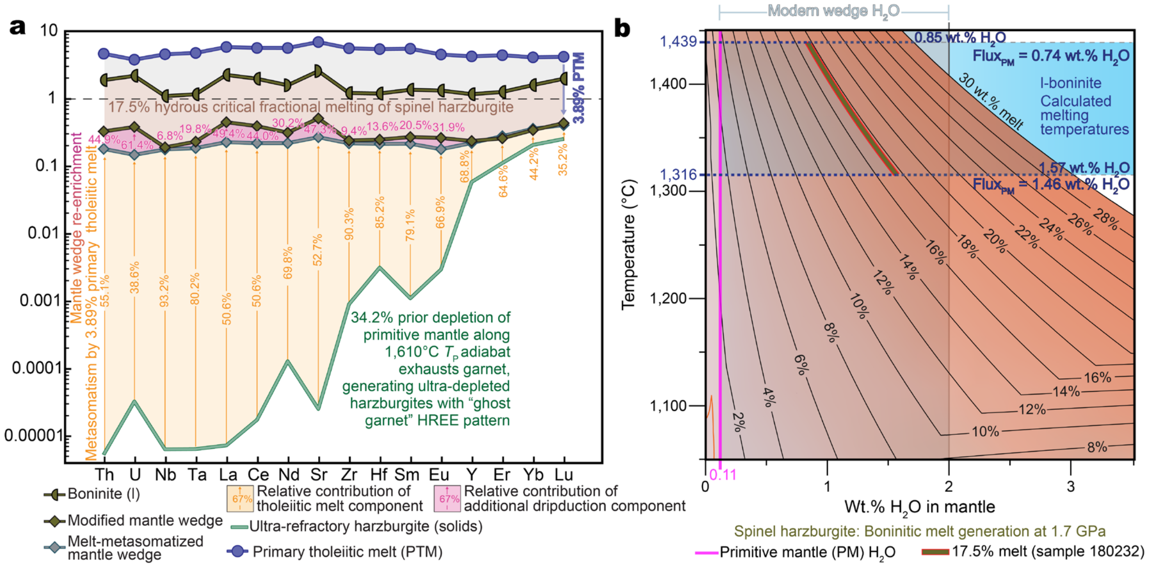

Supplementary Figure 9. **An alternative model for the formation of Whundo boninitic lavas**. (a) In addition to the model presented in the Main Text, two-stage melt modelling can also reproduce the trace element patterns of I-boninite sample 180232 by first generating a melt-free refractory harzburgitic solid through high-degree melting (past garnet exhaustion) of primitive mantle along a 1,610°C T_P adiabat, and then subjecting the harzburgitic solid to metasomatism by 3.89 wt.% primitive tholeiitic melt. This melt-metasomatized source then undergoes modification and melting, producing I-boninites. The need for additional re-enrichment by a component other than a primary tholeiitic melt contributions to the modified mantle wedge source of the boninites also rules out a purely melt-enrichment scenario. (b) Similar to the main model, the alternative model requires the addition of at least between 0.74 and 1.46 wt.% H_2O to produce a 18% flux melt of clinopyroxene-poor spinel harzburgite at 1.70 GPa. Modelling details are provided in the Methods, Supplementary Methods, Supplementary Figs. 10-11, and Supplementary Data 3 and 6.

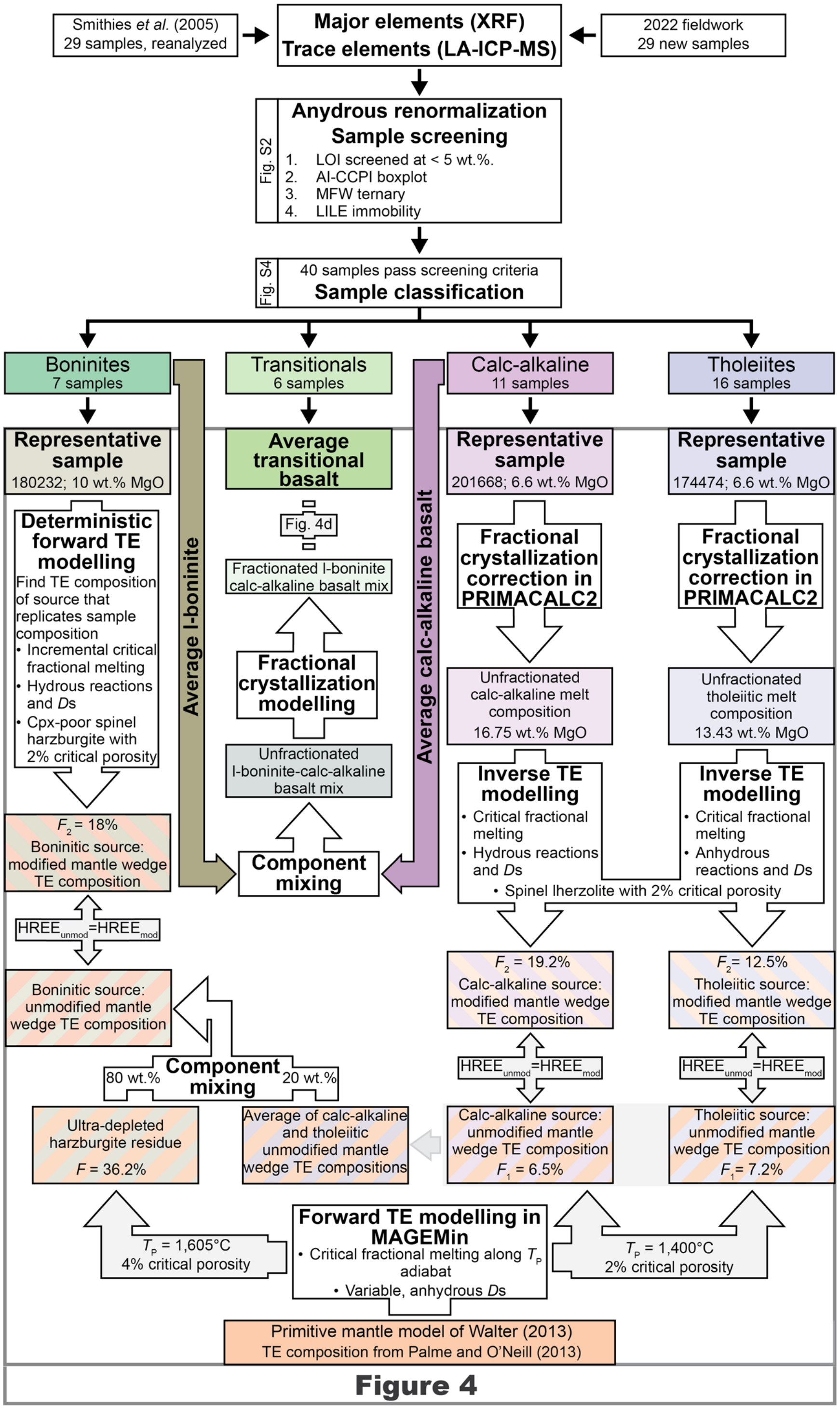

Supplementary Figure 10. **Analytical and trace element modelling workflow.** See Methods and Supplementary Methods for discussion. T_P denotes mantle potential temperature.

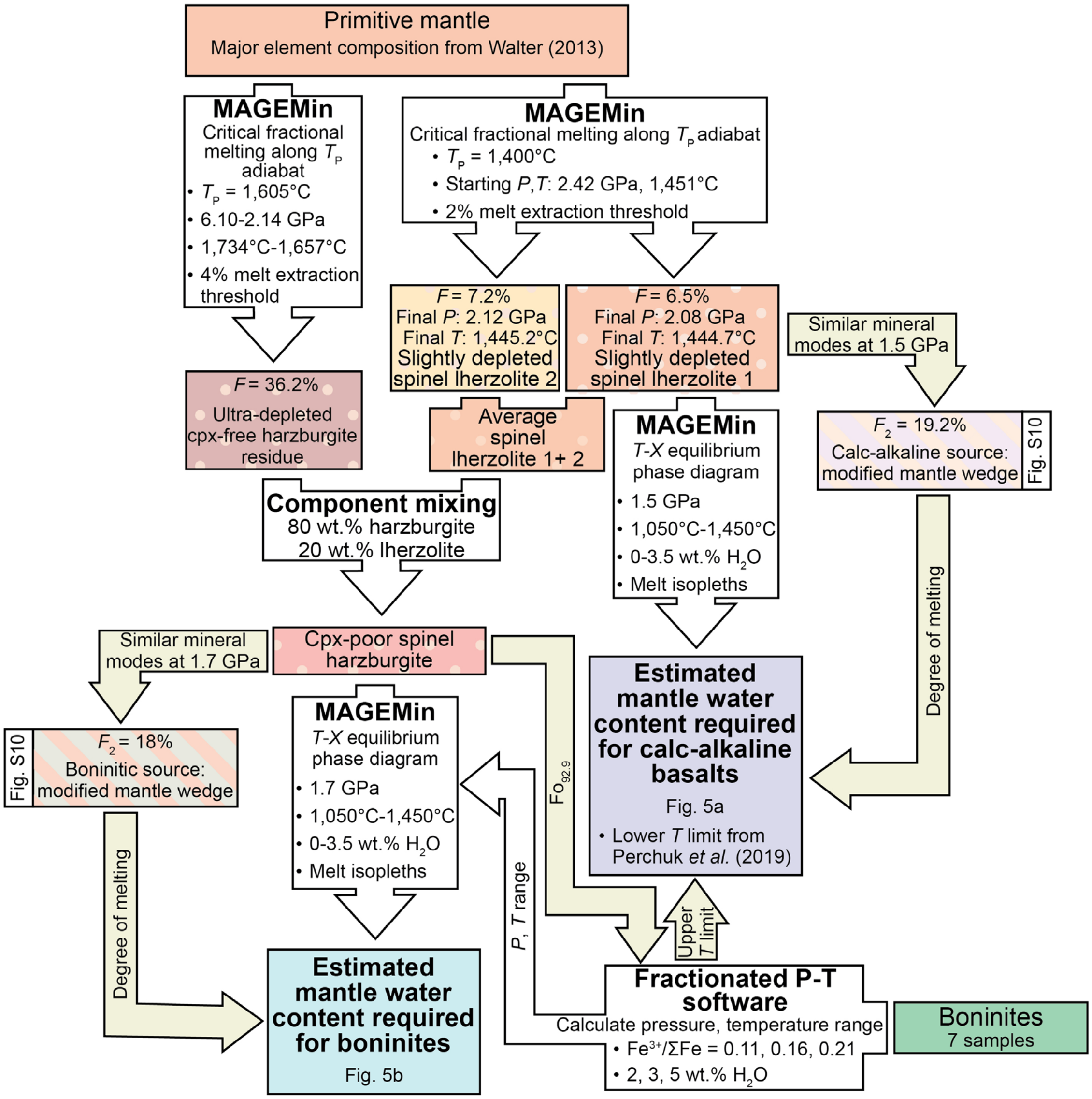

Supplementary Figure 11. **Thermodynamic modelling workflow.** See Methods and Supplementary Methods for discussion. T_P denotes mantle potential temperature.

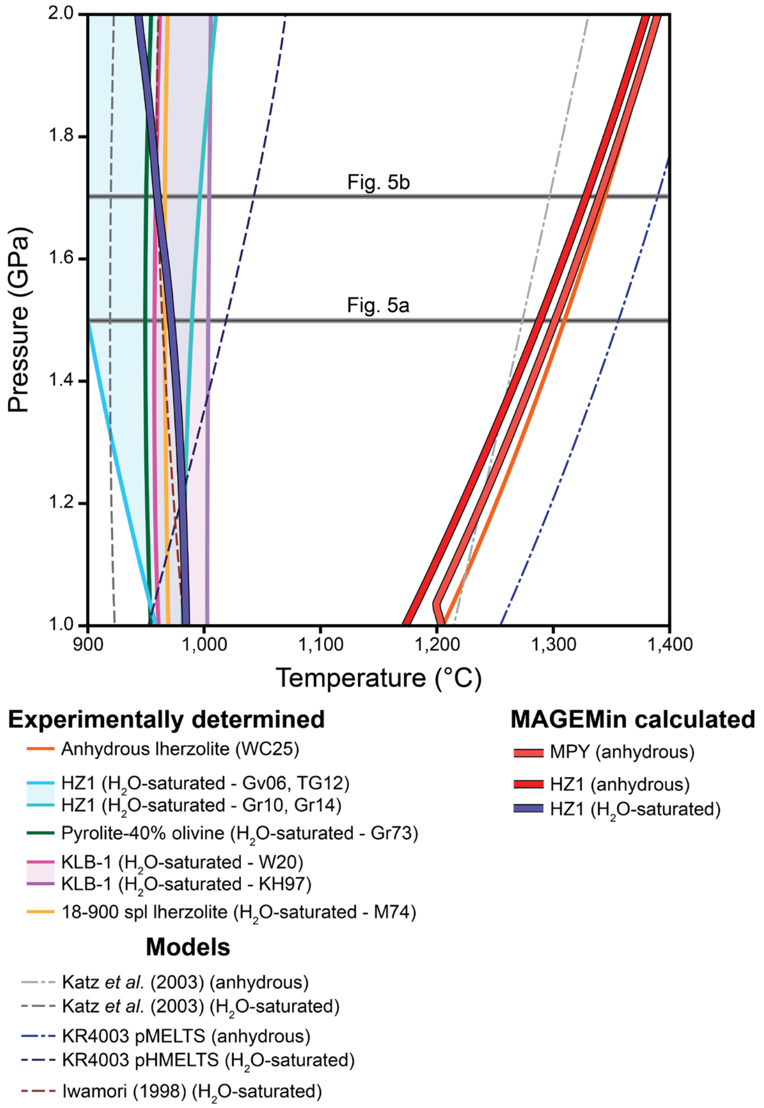

Supplementary Figure 12. **Comparison of the H_2O-saturated solidus as calculated in MAGEMin with the results of experimental studies and other thermodynamic and empirical models.** There remains substantial debate regarding the position of the H_2O-saturated solidus in P-T-X space, even on the scale of a single bulk composition, as evidenced by the differences between the results of the experimental studies of HZ1 from Grove *et al.* (GV06[45]), Till *et al.* (TG12[45]) vs Green *et al.* (GR10[60], GR14[61]) and KLB1 from Wang *et al.* (W20[75]) vs Kawamoto and Holloway (KH97[76]). These discrepancies are also evident in thermodynamic and empirical models (e.g., Katz *et al.*[77] vs pHMELTS[59] vs

Iwamori[78]), which appear to be magnified towards higher pressures. However, the MAGEMin-calculated H_2O-saturated solidus for HZ1 closely resembles the majority of experimentally-determined H_2O-saturated solidi, with similar temperatures at the pressures of interest for this study. Also shown are the anhydrous solidi for HZ1 and MPY[79] calculated in MAGEMin, KR4003 calculated in pMELTS and anhydrous lherzolite from Walter and Cottrell (WC25[58]). pMELTS and pHMELTS models for KR4003 are from ref. 58 using the alphaMELTS software[59]. H_2O-saturated solidi GR73 and M74 are from refs. 80 and 81, respectively.

3. SI Text

3.1. The Pilbara Craton, Sholl Terrane and Whundo Group

The 3.59-2.83 Ga Pilbara Craton of Western Australia contains some of the oldest well-preserved successions of mafic-ultramafic-felsic volcanic and subordinate sedimentary rocks on Earth[82]. Only one-sixth of the cratonic basement, predominantly in the north, is exposed; the remainder is buried underneath Neoarchean volcanics and Paleoproterozoic basin infill. The exposed portion of the craton consists of seven distinct lithotectonic blocks: the East Pilbara Terrane (EPT), the Karratha, Regal, and Sholl terranes, collectively referred to as the West Pilbara Superterrane (WPS), the Central Pilbara Tectonic Zone (CPTZ), and the Kurrana Terrane (Fig. 1a). The East Pilbara, Kurrana and Karratha terranes form the remnants of an Eoarchean[83] nucleic mafic plateau known as the Ancestral Pilbara Block (APB; ref. 73) typified by a characteristic dome-and-keel geometry of ovoid, largely tonalite-trondhjemite-granodiorite (TTG) series affinity plutons are encircled by narrow, steeply dipping 3.53-3.23 Ga greenstone belts[82]. These components were generated in concert during several volcano-plutonic magmatic pulses of tholeiitic affinity triggered by multiple instances of chondritic mantle upwellings beneath a hybrid mafic-felsic substrate[84,85].

The breakup of the APB during the 3.22-3.15 Ga East Pilbara Terrane Rifting Event split the plateau into its respective crustal block components, separating the Karratha Terrane from the East Pilbara Terrane by a zone of thin, juvenile mafic-ultramafic crust comprising the Central Pilbara Tectonic Zone and Regal Terrane[86] referred to as the “Regal Basin” by ref. 87. The lack of geochemical evidence for Paleoarchean basement underlying these lithotectonic blocks is inconsistent with their formation in a mafic plateau-like setting as part of the APB[1,73,87], suggesting that they may be examples of Archean crust that is rarely preserved in Paleo-Mesoarchean cratons. This juvenile crust forms the basement of the 3.13-3.10 Ga Sholl Terrane, comprising the 10 km-thick volcanic

succession of the Whundo Group and the coeval (ultra)mafic-felsic intrusives of the Railway Supersuite and Bullock Hide Intrusion[1,87] (Fig. 1a). The terrane is separated to the north from the Karratha Terrane by the 1 km-wide Sholl Shear Zone and is unconformably overlain to the south by the Neoarchean Fortescue Group Large Igneous Province (LIP) (Fig. 1a). Although the exposed portion of the Sholl Terrane is relatively small, geophysical evidence[87] suggests that a much larger portion (>50 km width) is buried beneath the Fortescue LIP.

The Whundo Group is formally divided (from top to bottom) into the Nallana, Tozer, Bradley Basalt and Woodbrook formations, but can also be geochemically divided into three volcanic cycles over 18 units (Fig. 1b). The Maitland Shear Zone forms the basal contact of the group, which was initially a low-angle thrust fault over the Railway Supersuite. The first cycle consists of alternating boninitic and calc-alkaline units locally interspersed by rhyolitic lavas and cryptodomes. The second cycle consists of a lower tholeiitic unit overlain by a boninite unit, in turn, overlain by a unit of intercalated tholeiitic and calc-alkaline lavas and capped by a felsic pyroclastic-dominant unit. The upper cycle consists of alternating units of boninite and tholeiite at its base, overlain by alternating calc-alkaline and adakitic units, and capped by an Nb-enriched basalt unit. The succession is metamorphosed chiefly to lower greenschist facies, and there is remarkable widespread preservation of large- and small-scale depositional features, such as hyaloclastites, pillows, varioles, vesicles, and scoria (Fig. 1c-e). Deformation is relatively minor, and there is no structural repetition of units[1]. Based on Nd isotopic signatures (indicating a maximum depleted mantle age of 3.25 Ga for the oldest crust of the terrane), the presence of interlayered calc-alkaline, boninitic and tholeiitic rocks, low Ce/Yb and Th/La, a lack of evidence for a felsic basement, and its juxtaposition with terranes of exotic affinities, ref. 1 proposed an oceanic arc setting for the Whundo Group.

4. SI References

1. Smithies, R. H., Champion, D. C., Van Kranendonk, M. J., Howard, H. M. & Hickman, A. H. Modern-style subduction processes in the Mesoarchaean: Geochemical evidence from the 3.12 Ga Whundo intra-oceanic arc. *Earth Planet. Sci. Lett.* **231**, 221–237 (2005).

2. Kelley, K. A. & Fischer, T. P. Melt inclusion and gas perspectives on volatiles in subduction zones. in *Treatise on Geochemistry* (eds. Anbar, A. D. & Weis, D.) 745–771 (Elsevier, 2025). doi:10.1016/B978-0-323-99762-1.00081-4.

3. Large, R. R., Gemmell, J. B., Paulick, H. & Huston, D. L. The Alteration Box Plot: A Simple Approach to Understanding the Relationship between Alteration Mineralogy and Lithogeochemistry Associated with Volcanic-Hosted Massive Sulfide Deposits. *Econ. Geol.* **96**, 957–971 (2001).

4. DIGIS Team, Data from "GEOROC Compilation: Convergent Margins." Göttingen Research Online. https://doi.org/10.25625/PVFZCE (2023).

5. EarthChem Portal. https://portal.earthchem.org/.

6. Ohta, T. & Arai, H. Statistical empirical index of chemical weathering in igneous rocks: A new tool for evaluating the degree of weathering. *Chem. Geol.* **240**, 280–297 (2007).

7. Zheng, Y. F. Subduction zone geochemistry. *Geosci. Front.* **10**, 1223–1254 (2019).

8. Ague, J. J. Element mobility during regional metamorphism in crustal and subduction zone environments with a focus on the rare earth elements (REE). *Am. Mineral.* **102**, 1796–1821 (2017).

9. Lacasse, C. M. *et al.* Restoring original composition of hydrothermally altered Archean metavolcanic rocks of the Carajás Mineral Province (Brazil): Geodynamic implications for the transition from lid to mobile tectonics. *Lithos* **372**–**373**, 105647 (2020).

10. Turner, S. J. & Langmuir, C. H. A quantitative framework for global variations in arc geochemistry. *Earth Planet. Sci. Lett.* **584**, 117411 (2022).

11. Mullen, E. K. & McCallum, I. S. Origin of Basalts in a Hot Subduction Setting: Petrological and Geochemical Insights from Mt. Baker, Northern Cascade Arc. *J. Petrol.* **55**, 241–281 (2014).

12. Wanke, M., Clynne, M. A., von Quadt, A., Vennemann, T. W. & Bachmann, O. Geochemical and petrological diversity of mafic magmas from Mount St. Helens. *Contrib. to Mineral. Petrol.* **174**, 0 (2019).

13. Wang, X., Nebel, O., Churchus, A. & Cawood, P. A. Tin isotope systematics in subduction zones. *Geochim. Cosmochim. Acta* **399**, 18–34 (2025).

14. Pearce, J. A. & Reagan, M. K. Identification, classification, and interpretation of boninites from Anthropocene to Eoarchean using Si-Mg-Ti systematics. *Geosphere* **15**, 1008–1037 (2019).

15. Winchester, J. A. & Floyd, P. A. Geochemical discrimination of different magma series and their differentiation products using immobile elements. *Chem. Geol.* **20**, 325–343 (1977).

16. O'Neill, H. S. C. The Smoothness and Shapes of Chondrite-normalized Rare Earth Element Patterns in Basalts. *J. Petrol.* **57**, 1463–1508 (2016).

17. Ross, P.-S. & Bédard, J. H. Magmatic affinity of modern and ancient subalkaline volcanic rocks determined from trace-element discriminant diagrams. *Can. J. Earth Sci.* **46**, 823–839 (2009).

18. Schmidt, M. W. & Jagoutz, O. The global systematics of primitive arc melts. *Geochemistry, Geophys. Geosystems* **18**, 2817–2854 (2017).

19. Kimura, J.-I. & Ariskin, A. A. Calculation of water-bearing primary basalt and estimation of source mantle conditions beneath arcs: PRIMACALC2 model for WINDOWS. *Geochemistry, Geophys. Geosystems* **15**, 1494–1514 (2014).

20. Matzen, A. K., Baker, M. B., Beckett, J. R. & Stolper, E. M. The temperature and pressure dependence of nickel partitioning between olivine and silicate melt. *J. Petrol.* **54**, 2521–2545 (2013).

21. Vandenburg, E. D. *et al.,* "A widespread, short-lived, off-craton subduction source for hidden crustal growth in Earth's infancy". *EGU General Assembly 2024*, Vienna, Austria, 14–19 Apr 2024, EGU24-2118 (2024).

22. Kelley, K. A. *et al.*, Mantle melting as a function of water content beneath back-arc basins. *J. Geophys. Res. Solid Earth* **111** (2006).

23. Gao, L. *et al.*, Oxidation of Archean upper mantle caused by crustal recycling. *Nat. Commun.* **13**, 3283 (2022).

24. Plank, T., Kelley, K. A., Zimmer, M. M., Hauri, E. H. & Wallace, P. J. Why do mafic arc magmas contain ~4 wt% water on average? *Earth Planet. Sci. Lett.* **364**, 168–179 (2013).

25. Pearce, J. A. & Parkinson, I. J. Trace element models for mantle melting: Application to volcanic arc petrogenesis. *Geol. Soc. Spec. Publ.* **76**, 373–403 (1993).

26. Stolper, E. & Newman, S. The role of water in the petrogenesis of Mariana trough magmas. *Earth Planet. Sci. Lett.* **121**, 293–325 (1994).

27. Shaw, D. M. Trace element fractionation during anatexis. *Geochim. Cosmochim. Acta* **34**, 237–243 (1970).

28. Rollinson, H. & Pease, V. Using Trace Element Data. in *Using Geochemical Data* 96–156 (2021). doi:10.1017/9781108777834.007.

29. König, S. *et al.*, Boninites as windows into trace element mobility in subduction zones. *Geochim. Cosmochim. Acta* **74**, 684–704 (2010).

30. McDade, P., Blundy, J. D. & Wood, B. J. Trace element partitioning between mantle wedge peridotite and hydrous MgO-rich melt. *Am. Mineral.* **88**, 1825–1831 (2003).

31. Albarède, F. Trace elements in magmatic processes. in *Introduction to Geochemical Modeling* 477–525 (Cambridge University Press, 1995). doi:10.1017/CBO9780511622960.011.

32. Forsyth, D. W. *et al.*, Imaging the Deep Seismic Structure Beneath a Mid-Ocean Ridge: The MELT Experiment. *Science (80-.).* **280**, 1215–1218 (1998).

33. Niu, Y. Mantle melting and melt extraction processes beneath ocean ridges: Evidence from abyssal peridotites. *J. Petrol.* **38**, 1047–1074 (1997).

34. Kinzler, R. J. Melting of mantle peridotite at pressures approaching the spinel to garnet transition: Application to mid-ocean ridge basalt petrogenesis. *J. Geophys. Res. Solid Earth* **102**, 853–874 (1997).

35. Grove, T. L., Till, C. B. & Krawczynski, M. J. The Role of H_2O in Subduction Zone Magmatism. *Annu. Rev. Earth Planet. Sci.* **40**, 413–439 (2012).

36. Gaetani, G. A. Grove, T. L. The influence of water on melting of mantle peridotite. *Contrib. to Mineral. Petrol.* **131**, 323–346 (1998).

37. Shervais, J. W. *et al.*, Magmatic Response to Subduction Initiation, Part II: Boninites and Related Rocks of the Izu-Bonin Arc From IODP Expedition 352. *Geochemistry, Geophys. Geosystems* **22**, 1–34 (2021).

38. Riel, N., Kaus, B. J. P., Green, E. C. R. & Berlie, N. MAGEMin, an Efficient Gibbs Energy Minimizer: Application to Igneous Systems. *Geochemistry, Geophys. Geosystems* **23**, 1–27 (2022).

39. Green, E. C. R., Holland, T. J. B., Powell, R., Weller, O. M. & Riel, N. Corrigendum to: Melting of Peridotites through to Granites: a Simple Thermodynamic Model in the System KNCFMASHTOCr, and, a Thermodynamic Model for the Subsolidus Evolution and Melting of Peridotite. *J. Petrol.* **66**, 1–3 (2025).

40. Otto, T., Stevens, G., Mayne, M. J. & Moyen, J. F. Phase equilibrium modelling of partial melting in the upper mantle: A comparison between different modelling methodologies and experimental results. *Lithos* **444–445**, 107111 (2023).

41. Weller, O. M. *et al.* New Thermodynamic Models for Anhydrous Alkaline-Silicate Magmatic Systems. *J. Petrol.* **65**, (2024).

42. Holland, T. J. B., Green, E. C. R. & Powell, R. Melting of Peridotites through to Granites: A Simple Thermodynamic Model in the System KNCFMASHTOCr. *J. Petrol.* **59**, 881–900 (2018).

43. Holland, T. J. B., Green, E. C. R. & Powell, R. A thermodynamic model for feldspars in $KAlSi_3O_8$-$NaAlSi_3O_8$-$CaAl_2Si_2O_8$ for mineral equilibrium calculations. *J. Metamorph. Geol*. **40**, 1-14 (2021).

44. Tomlinson, E. L. & Holland, T. J. B. A Thermodynamic Model for the Subsolidus Evolution and Melting of Peridotite. *J. Petrol.* **62**, 1–23 (2021).

45. Green, E. C. R. *et al.*, Activity–composition relations for the calculation of partial melting equilibria in metabasic rocks. *J. Metamorph. Geol.* **34**, 845–869 (2016).

46. Walter, M. J. Melt Extraction and Compositional Variability in Mantle Lithosphere. in *Treatise on Geochemistry: Second Edition* vol. 3 393–419 (Elsevier Ltd., 2013).

47. Zhu X., Duan W., Gerya T., Zhou X. and Tian J. (2026) The Mantle $Fe^{3+}/\Sigma Fe$ Ratio Has Doubled Since the Early Archean. *Nat. Commun.* **17**, 429.

48. Palme, H. & O'Neill, H. S. C. Cosmochemical Estimates of Mantle Composition. in *Treatise on Geochemistry: Second Edition* vol. 3 1–39 (Elsevier Ltd., 2013).

49. Sossi, P. A. *et al.* Petrogenesis and Geochemistry of Archean Komatiites. *J. Petrol.* **57**, 147–184 (2016).

50. Puchtel, I. S. & Arndt, N. T. Archean to recent komatiites and basalts. in *Treatise on Geochemistry* (eds. Anbar, A. D. & Weis, D.) 119–158 (Elsevier, 2025). doi:10.1016/B978-0-323-99762-1.00065-6.

51. Waterton, P. & Arndt, N. Komatiites: their geochemistry and origins. *The Archean Earth* 235–256 (2025) doi:10.1016/B978-0-323-95547-8.00016-1.

52. Wilson, M. Partial melting processes in the Earth's upper mantle. in *Igneous Petrogenesis* 37–72 (Springer Netherlands, 2007). doi:10.1007/978-94-010-9388-0_3.

53. Lord Rayleigh, LIX. On the distillation of binary mixtures. *London, Edinburgh, Dublin Philos. Mag. J. Sci.* **4**, 521–537 (1902).

54. Lee, C. T. A., Luffi, P., Plank, T., Dalton, H. & Leeman, W. P. Constraints on the depths and temperatures of basaltic magma generation on Earth and other terrestrial planets using new thermobarometers for mafic magmas. *Earth Planet. Sci. Lett.* **279**, 20–33 (2009).

55. Brounce, M., Kelley, K. A., Cottrell, E. & Reagan, M. K. Temporal evolution of mantle wedge oxygen fugacity during subduction initiation. *Geology* **43**, 775–778 (2015).

56. Waters, L. E. Geochemical modeling. in *Treatise on Geochemistry* (eds. Anbar, A. D. & Weis, D.) 137–182 (Elsevier, 2025). doi:10.1016/B978-0-323-99762-1.00017-6.

57. Miller, L. A., O'Neill, H. S. C., Berry, A. J. & Le Losq, C. Fractional crystallisation of eclogite during the birth of a Hawaiian Volcano. *Nat. Commun.* **13**, 2946 (2022).

58. Walter, M. & Cottrell, E. Equilibrium constraints on partial melting in the upper mantle. in *Treatise on Geochemistry* (eds. Anbar, A. D. & Weis, D.) 231–273 (Elsevier, 2025). doi:10.1016/B978-0-323-99762-1.00126-1.

59. Smith, P. M. & Asimow, P. D. Adiabat_1ph: A new public front-end to the MELTS, pMELTS, and pHMELTS models. *Geochemistry, Geophys. Geosystems* **6**, 1–8 (2005).

60. Green, D. H., Hibberson, W. O., Kovács, I. & Rosenthal, A. Water and its influence on the lithosphere–asthenosphere boundary. *Nature* **467**, 448–451 (2010).

61. Green, D. H. *et al.* Experimental study of the influence of water on melting and phase assemblages in the upper mantle. *J. Petrol.* **55**, 2067–2096 (2014).

62. Grove, T. L., Chatterjee, N., Parman, S. W. & Médard, E. The influence of H2O on mantle wedge melting. *Earth Planet. Sci. Lett.* **249**, 74–89 (2006).

63. Till, C. B., Grove, T. L. & Withers, A. C. The beginnings of hydrous mantle wedge melting. *Contrib. to Mineral. Petrol.* **163**, 669–688 (2012).

64. Caro, G., Morino, P., Mojzsis, S. J., Cates, N. L. & Bleeker, W. Sluggish Hadean geodynamics: Evidence from coupled ^{146,147}Sm–^{142,143}Nd systematics in Eoarchean supracrustal rocks of the Inukjuak domain (Québec). *Earth Planet. Sci. Lett.* **457**, 23–37 (2017).

65. Grocolas, T., Bouilhol, P., Caro, G. & Mojzsis, S. J. Eoarchean subduction-like magmatism recorded in 3750 Ma mafic–ultramafic rocks of the Ukaliq supracrustal belt (Québec). *Contrib. to Mineral. Petrol.* **177**, 1–27 (2022).

66. O'Neil, J., Francis, D. & Carlson, R. W. Implications of the Nuvvuagittuq Greenstone Belt for the Formation of Earth's Early Crust. *J. Petrol.* **52**, 985–1009 (2011).

67. Polat, A., Hofmann, A. . & Rosing, M. . Boninite-like volcanic rocks in the 3.7–3.8 Ga Isua greenstone belt, West Greenland: geochemical evidence for intra-oceanic subduction zone processes in the early Earth. *Chem. Geol.* **184**, 231–254 (2002)..

68. Hoffmann, J. E. *et al.*, Highly depleted Hadean mantle reservoirs in the sources of early Archean arc-like rocks, Isua supracrustal belt, southern West Greenland. *Geochim. Cosmochim. Acta* **74**, 7236–7260 (2010).

69. Frei, R., Polat, A. & Meibom, A. The Hadean upper mantle conundrum: Evidence for source depletion and enrichment from Sm-Nd, Re-Os, and Pb isotopic compositions in 3.71 Gy boninite-like metabasalts from the Isua

Supracrustal Belt, Greenland. *Geochim. Cosmochim. Acta* **68**, 1645–1660 (2004).

70. Furnes, H., Rosing, M., Dilek, Y. & de Wit, M. Isua supracrustal belt (Greenland)-A vestige of a 3.8 Ga suprasubduction zone ophiolite, and the implications for Archean geology. *Lithos* **113**, 115–132 (2009).

71. Szilas, K., Kelemen, P. B. & Rosing, M. T. The petrogenesis of ultramafic rocks in the >3.7Ga Isua supracrustal belt, southern West Greenland: Geochemical evidence for two distinct magmatic cumulate trends. *Gondwana Res.* **28**, 565–580 (2015).

72. Pearce, J. A. & Arculus, R. J. Boninites. in *Encyclopedia of Geology* 113–129 (Elsevier, 2021). doi:10.1016/B978-0-08-102908-4.00152-1.

73. Vandenburg, E. D. *et al.*, Spatial and temporal control of Archean tectonomagmatic regimes. *Earth-Science Rev.* **241**, 104417 (2023).

74. Davidson, J., Turner, S. & Plank, T. Dy/Dy*: Variations Arising from Mantle Sources and Petrogenetic Processes. *J. Petrol.* **54**, 525–537 (2013).

75. Wang, J. *et al.* The Water-Saturated Solidus and Second Critical Endpoint of Peridotite: Implications for Magma Genesis Within the Mantle Wedge. *J. Geophys. Res. Solid Earth* **125**, 1–16 (2020).

76. Kawamoto, T. & Holloway, J. R. Melting temperature and partial melt chemistry to H_2O-saturated mantle peridotite to 11 gigapascals. *Science (80).* **276**, 240–243 (1997).

77. Katz, R. F., Spiegelman, M. & Langmuir, C. H. A new parameterization of hydrous mantle melting. *Geochemistry, Geophys. Geosystems* **4**, 1–19 (2003).

78. Iwamori, H. Transportation of H_2O and melting in subduction zones. *Earth Planet. Sci. Lett.* **160**, 65–80 (1998).

79. Robinson, J. A. C., Wood, B. J. & Blundy, J. D. The beginning of melting of fertile and depleted peridotite at 1.5 GPa. *Earth Planet. Sci. Lett.* **155**, 97–111 (1998).

80. Green, D. H. Experimental melting studies on a model upper mantle composition at high pressure under water-saturated and water-undersaturated conditions. *Earth Planet. Sci. Lett.* **19**, 37–53 (1973).

81. Millhollen, G. L., Irving, A. J. & Wyllie, P. J. Melting Interval of Peridotite with 5.7 per Cent Water to 30 Kilobars. *J. Geol.* **82**, 575–587 (1974).

82. Smithies, R. H., Champion, D. C., Van Kranendonk, M. J. & Hickman, A. H. . *Geochemistry of volcanic units of the northern Pilbara Craton, Western Australia*. *Geological Survey of Western Australia* vol. 104 (2007).

83. Petersson, A., Kemp, A. I. S. & Whitehouse, M. J. A Yilgarn seed to the Pilbara Craton (Australia)? Evidence from inherited zircons. *Geology* **47**, 1098–1102 (2019).

84. Hasenstab, E. *et al.* Evolution of the early to late Archean mantle from Hf-Nd-Ce isotope systematics in basalts and komatiites from the Pilbara Craton. *Earth Planet. Sci. Lett.* **553**, 116627 (2021).

85. Kemp, A. I. S., Vervoort, J. D., Petersson, A., Smithies, R. H. & Lu, Y. A linked evolution for granite-greenstone terranes of the Pilbara Craton from Nd and Hf isotopes, with implications for Archean continental growth. *Earth Planet. Sci. Lett.* **601**, 117895 (2023).

86. Van Kranendonk, M. J., Hugh Smithies, R., Hickman, A. H., Wingate, M. T. D. & Bodorkos, S. Evidence for Mesoarchean (~3.2Ga) rifting of the Pilbara Craton: The missing link in an early Precambrian Wilson cycle. *Precambrian Res.* **177**, 145–161 (2010).

87. Hickman, A. H. *Archean Evolution of the Pilbara Craton and Fortescue Basin*. vol. 24 (Springer International Publishing, 2023).
