# Supplementary material for: Modern arc-like water content in the source of 3.1-billion-year-old volcanic rocks
Source: Nat Commun. 2026 Jul 7;17:5630. doi: 10.1038/s41467-026-74653-1 (PMC13342610; doi:10.1038/s41467-026-74653-1)
Supplement: Supplementary file 2 — Description of Additional Supplementary Files [file 41467_2026_74653_MOESM2_ESM.pdf]

Description of Additional Supplementary Files

Supplementary Data 1. Details and chemical compositions of the samples in this study.

Supplementary Data 2. Trace element abundances of reference materials and sample duplicates analyzed in this study.

Supplementary Data 3. Trace element composition and parameters of modelled modified-, pre-dripduction mantle wedge and slab mass balance contributions.

Supplementary Data 4. Melt-mineral partition coefficients used for modelling in this study.

Supplementary Data 5. Spreadsheets used to calculate modified mantle wedge data.

Supplementary Data 6. Elemental inputs for MAGEMin melting models.

Supplementary Data 7. MAGEMin model outputs used for stage 1 depletion.

Supplementary Data 8. Trace element modelling inputs and outputs for stage 1 depletion of Whundo mantle sources.

Supplementary Data 9. PRIMACALC2 inputs and outputs for the fractional crystallization correction of calc-alkaline and tholeiitic basalts.

Supplementary Data 10. Inputs and results of mixing-fractional crystallization modelling for transitional boninitic-calc alkaline basalts.

Supplementary Data 11. Results of λ modelling of melting of Whundo boninite second-stage sources, source offsets, and source enrichment trends.

Supplementary Data 12. Melting *P-T* conditions for the primary boninitic magmas, as determined by Fractionated P-T software.

Supplementary Data 13. Raw MAGEMin output data.
