# Supplementary material for: Modern arc-like water content in the source of 3.1-billion-year-old volcanic rocks
Source: Nat Commun. 2026 Jul 7;17:5630. doi: 10.1038/s41467-026-74653-1 (PMC13342610; doi:10.1038/s41467-026-74653-1)
Supplement: Supplementary file 16 — Transparent Peer Review file [file 41467_2026_74653_MOESM16_ESM.pdf]

nature portfolio

Peer Review File

Modern arc-like water content in the source of 3.1-billion-year-old volcanic rocks

Corresponding Author: Dr Eric Vandenburg

This manuscript has been previously reviewed at another journal. This document only contains information relating to versions considered at Nature Communications. Mentions of the other journal have been redacted.

This file contains all reviewer reports in order by version, followed by all author rebuttals in order by version.

Attachments originally included by the reviewers as part of their assessment can be found at the end of this file.

Version 0:

Reviewer comments:

Reviewer #1

(Remarks to the Author)
Review of Eric D. Vandenburg et al.
Modern arc-like water content in the source of 3.1-billion-year-old volcanic rocks
Submitted to Nature Comms

This is now the third time I have seen this ms and it has evolved considerably in the revision process. In their response, the authors have addressed my previous comments and made appropriate changes to their ms, which in my view are adequate.

In essence the problem is how to create an enriched mantle in the early Earth before, perhaps, the advent of subduction. In the modern Earth this would normally be done through the introduction of a fluid-mobile element enriched fluid into the mantle to create an enriched source and trigger melting. The dilemma is how did this happen in the Archaean.

The authors have now abandoned a subduction model and are advocating a drip-duction model, which could work – its fashionable. The geodynamic model is supported by a complex array of geochemical calculations modelling the observed rock compositions.

I have two comments.
1. The proposed geochemical model is probably not unique – in other words there may be other ways of modelling the observed rock compositions. I would therefore urge some caution in the text indicating that other solutions may be forthcoming and so whilst this model satisfies the geochemical constraints it may not be the only solution.
2. As indicated by one of the other reviewers I am not persuaded that the model makes much sense in terms of the rather complex and varied stratigraphy. Unlike the example of the Oman ophiolite cited, where the different lava sequences have a sense of uniformity and stratigraphic progression, this is much less clear in this study.

I feel inclined to say let this discussion now go into the public domain and see how other workers respond.

Hugh Rollinson
University of Derby
27/11/25

Reviewer #2

(Remarks to the Author)
Review of Vandenburg et al.
Modern arc-like water content in the source of 3.1-billion-year old volcanic rocks

Submitted to [REDACTED]

This manuscript is a resubmission that several others have reviewed, and the revisions from the previous review are substantial. The manuscript and supplementary files present a detailed study of 3.1 Ga volcanic rocks of the Whundo Group in the Pilbara Craton, Australia. The authors use these rocks to assess the possibility of subduction before 2.8 Ga. The focus point of this manuscript is that hydrous melting initiates early subduction, or dripduction, at 3.1 Ga. They conclude that, despite operating under different conditions, the system is similar to modern volcanic arc systems. Overall, this manuscript is well written and detailed, and it presents an argument supported by the data. I hope my suggestions help the authors and look forward to seeing this manuscript in publication.

Comments:
1. Chemical Alteration and Weathering of Samples. I was specifically asked to assess the issue of alteration in these rocks. I used the author's data to determine alteration indices from several methods designed to examine chemical weathering and hydrothermal alteration of igneous rocks, including the Chemical Alteration Index (Nesbitt and Young, 1982), the chlorite-carbonate-pyrite index (CCPI), and the Ishikawa Alteration Index (AI; Ishikawa et al., 1976). The full data set includes several samples that exhibit evidence of chemical weathering and hydrothermal alteration, but these are the same samples that were filtered out based on LOI >5 wt%. The filtered dataset used by the authors for the models shows indices that suggest minimal alteration and chemical weathering. The authors made a similar statement in response to reviewer #1 and reached a similar conclusion about the level of alteration these indices suggest.

Compared to modern arc volcanic rocks, the CCPI Index suggests some Mg-Fe chlorite alteration, with an average index of 87% and a minimum of 75%. Modern unaltered arc basalts range between a CCPI index of 70-85%, and the authors suggest that the tholeiites do not resemble other early Earth subduction-related rocks and therefore could be formed in a divergent setting, which could result in higher initial FeO and MgO contents. Comparing this index to LILE element contents within the filtered sample shows a slight correlation with the Ishikawa AI. Still, the overall variability of elements such as Rb, at only 40 ppm, is well within that observed in modern arc volcanic systems. The same is true of other LILE elements modeled, as illustrated by minimal variation in Ba/Sr ratio and Rb/Sr ratio.

Overall, I believe the authors appropriately filtered the data and restricted the dataset to unaltered samples for their interpretations. While I do believe there is some amount of alteration/metamorphism that must have occurred in rocks >3 Ga, it appears it is minimal in the selected samples and do not affect the overall interpretation.

2. Thermodynamic Model and Equations. I thank the authors for the detailed workflow for deriving Equation #4 and for the thermodynamic model. These allowed me to easily recreate the models and mantle sources used for the interpretations and conclusions.

The evidence for fluid flux melting is difficult to follow, and the text takes a long time to develop the concept. The figures highlighting this are presented in the supplementary material, with the need to go between Figure 2 and Supplementary Figure 7. There is a figure in the reviewer responses, Figure R2, that makes this argument clear and directly links it to the argument for fluid-flux melting versus other melting processes.

The authors also use PM-normalized REE shape parameters (lines 302-317) to interpret mantle-source melting. This is the only are in the modelling that needs more description. For example, the authors state (line 304-307): “Our mantle melting models in REE shape ($\lambda1$-$\lambda2$) space indicate the Whundo boninites were derived from 6-17.5% melting of a clinopyroxene-poor harzburgitic mantle source, itself derived from a prior depletion event that melted a source to a point past garnet exhaustion.” They do not provide ranges to be of the $\lambda1$-$\lambda2$ space that are expected for garnet-stable, versus garnet-residual sources, or the differences between harzburgite source shape versus lherzolite. This is, overall, a minor point, but when the authors are trying to argue for “ghost garnet” signatures (or lack thereof) by pre-depletion of garnet before mantle melting, having some knowledge of these ranges could make the argument easier to understand and could reduce the inference that the data is over-interpreted.

References Cited in Review:
R. R. Large, J. B. Gemmell, H. Paulick, D. L. Huston, The Alteration Box Plot: A Simple Approach to Understanding the Relationship between Alteration Mineralogy and Lithogeochemistry Associated with Volcanic-Hosted Massive Sulfide Deposits. Econ. Geol. 96, 957–971 (2001).

Y. Ishikawa, T. Sawaguchi, S. Iwaya, and M. Horiuchi, Delineation of prospecting targets for Kuroko deposits based on modes of volcanism of underlying dacite and alteration halos. Mining Geology, 26, 105–117 (1976) (in Japanese with English abs.)

H. Nesbitt, and G. Young, Early Proterozoic climates and plate motions inferred from major element chemistry of lutites. Nature 299, 715–717 (1982). https://doi.org/10.1038/299715a0

Reviewer #3

(Remarks to the Author)
General comments on Vandenburg et al.

“Modern arc-like water content in the source of 3.1-billion-year-old volcanic rocks” or “Earth's first primitive subduction occurred in the missing 2 majority of Archean crust”

This is the first time I’m reading this manuscript, but I understand it has already been through some rounds of revision. I suspect some of my comments might have been previously raised, but that happens when joining an on-going revision. The manuscript is polished and well-written presenting a series of samples with boninite signature, which is rightfully uncommon during the Archean and only known on a handful of locations. The identification and description of those rocks alone would be worth of publication – even considering the boninite like signature of the Whundo group was already mentioned by Smithies et al. (2005); However, the authors focused on an elaborate petrological model instead. My major concern is the very basic assumption that the rocks are pristine and represent lavas, with REE, HFSE and LILE elements preserved. At the current state of the manuscript, I’m not convinced that is the case.
The REE and HFSE could likely represent the original signature, but expanding that to the LILE (K, Rb, Ba, Pb, Ce etc.) is much more difficult. I’ll use as an example the work by Shervais et al. (2021), reference 74. of the manuscript. Their work drilled 800 meters into the boninite type locality, and show that K, Rb, Ce are significantly altered with minor mobility on Pb, Ba, Sr. The lack of correlation with MgO is used as argument for mobility, and further conclusion about those elements are very limited. The lack of correlation with Rb presented on figure sup 2 c and b of the manuscript is similar to the lack of correlation found on boninites, but that doesn’t make the sample boninites nor show that their LILE are preserved. I find it incredibly difficult to understand that, for an Archean boninite that has been tectonized to at least some extent, that those elements are preserved. Even at low temperature sea-floor alteration, low-temperature metamorphism, or subaerial exposure those elements are known to be mobilized. Therefore, a major aspect of the conclusions should be reconsidered, and I believe the data is overinterpreted in those terms. Additionally, there are signs of contamination that needs to be addressed.
The authors put a lot of effort to explain the models, which I appreciate. I think the calculations itself are reasonable, and only some minor changes would be needed. My main question is why the mixing and fractional crystallization (section 1.6) wasn’t done using alphamelts, which technically is very similar to an AFC model. That would provide an entirely consistent thermodynamic model. I’ve also added a collaborator as a co-reviewer who’s very qualified in thermodynamic modelling who’s providing a co-revision on the calculations.
My main suggestion for the manuscript is focusing on presenting a well-characterized description of the samples with petrography and outcrop photos – essentially circling back to some images that seem to be progressively vanishing with each revision. Archean boninites are rare and on it is own represent a significant finding and should be very carefully presented before elaborating on geochemical models. I’d suggest a major revision where all the considerations about the LILE are readdressed. Implications regarding the dripduction and REE and maybe HFSE are fine.

Specific comments are on the attached .doc file.

[Editorial Note: See attachment at end of file]

Reviewer #4

(Remarks to the Author)
The manuscript addresses a highly interesting topic, combining geochemical data with thermodynamic modeling, with particular emphasis on the role of water in the formation of boninite and calc-alkaline rocks during the Archean, prior to the onset of plate tectonics. I was invited as a co-reviewer specifically to evaluate the methodological aspects of the study; therefore, my comments will focus on part of the methodology (modeling) and will not address interpretations or discussions presented beyond that section.

1) Main Text:
Most of my comments in the main text relate to the figures. Overall, the figures would benefit from improved readability and visual optimization. In several cases, font sizes are too small or lack sufficient contrast to be easily read at a standard 100% page scale. Adjusting font sizes, improving color contrast, and adding clearer backgrounds where necessary would significantly enhance figure clarity and accessibility.
Figure 1: The legends in the map (a) and stratigraphic column (b) are too small and difficult to read without zooming in beyond 100% of the page. These should be adjusted to an appropriate font size to facilitate readability. In addition, the text within the field photographs is hard to read at the current figure size. If the size is maintained, I suggest adding a solid background to the text rather than relying solely on shadowing.
Figure 3: All graphs use a range from 0.1 to 100, resulting in approximately two-thirds of the plotted area being blank. While I understand the intention to maintain a consistent scale across panels, I suggest reducing the lower limit of the range (e.g., starting at ~0.8), which would eliminate a large unused portion of the plots. This adjustment would make similarities and differences between the traces more apparent, as the current wide range tends to visually flatten the data.
Figure 4: The internal numbering within the figure panels is small and difficult to read and should be increased for better clarity.
Figure 6: Some font sizes and color choices also hinder readability and would benefit from adjustment to improve contrast and legibility.

2) Method and Supplementary Text 1
The manuscript presents a technically demanding and time-consuming modeling study. While such models are valuable tools for exploring rock-forming processes, they must be firmly grounded in geological evidence. At several points in the manuscript, the distinction between measured parameters and inferred or assumed parameters is difficult to follow, which

complicates assessment of the model robustness.

A critical issue concerns the characterization of the studied rocks. Much of the study is based on the premise that the rocks are pristine. Given that the samples are described as Archean boninites, it is essential to demonstrate convincingly that they preserve primary igneous features. Archean rocks are rarely pristine and are commonly affected by metamorphism and/or weathering. Therefore, the central premise of the manuscript (that the models represent primary igneous rock-forming processes) requires explicit and well-documented supporting evidence.
Although the authors state that the samples are neither metamorphosed nor weathered, this claim is not supported by petrographic evidence in the current manuscript. The study relies heavily on thermodynamic and geochemical modeling, yet provides no thin-section documentation demonstrating the preservation of primary igneous mineral assemblages and textures. This represents a significant shortcoming.
Petrography is a fundamental starting point in geological studies, particularly when dealing with Archean rocks, which are rarely pristine and are commonly affected by metamorphism, deformation, and/or alteration. Petrographic observations are necessary to assess the presence or absence of metamorphic recrystallization, deformation fabrics, and secondary alteration phases that could substantially influence whole-rock compositions. Without such information, it is difficult to evaluate whether the compositions used in the models genuinely represent primary magmatic conditions.
The authors rely primarily on geochemical diagrams to argue for the pristine nature of the samples. While informative, geochemical evidence alone is insufficient without complementary petrographic documentation. The inclusion of representative thin-section images (in plane- and cross-polarized light) showing preserved igneous textures would substantially strengthen the manuscript and increase confidence in the modeling results.
In the absence of clear petrographic evidence for preserved igneous textures, the central premise of the study remains insufficiently supported. Addressing this issue is essential and, in my view, necessary.

Alteration boxplot method.
One of the ways the authors attempt to “prove” that the rocks were not affected by metamorphism is by using the alteration box plot method published by Large et al. (2001). According to the original paper describing this method, it has several limitations: (a) it was developed for the study of alteration associated with VHMS systems, which is not the case here; (b) “the box plot is most suited to the study of hydrothermal alteration in felsic volcanics rather than mafic volcanics”; and (c) the authors clearly state that “the alteration box plot should not be used in isolation but is most powerful in the understanding of alteration when combined with petrographic and/or mineralogical studies.”
Furthermore, this reinforces my comment above: the simplest way to demonstrate and convincingly show that the rocks are neither altered nor metamorphosed is to present photomicrographs of thin sections documenting pristine volcanic textures and primary mineralogy, as well as the absence of metamorphic phases. The field photographs provided are not sufficient to demonstrate this, as the rocks appear weathered in the images.
Although the authors explain that weathered material was removed prior to geochemical analysis (a common and necessary step in geochemical studies, and one that is also reflected in the weathering plot) additional evidence is required to rule out metamorphic or hydrothermal overprinting.
Regarding the statement: “Moreover, Rb shows no correlation with loss-on-ignition (LOI), which is often invoked as a proxy for the degree of alteration, particularly in mafic–ultramafic rocks.” The absence of correlation alone in this graph is not sufficient to demonstrate a lack of alteration. Also, If LOI vs Rb standalone is indeed considered a “common” proxy for alteration, appropriate references supporting this assumption should be provided to support.

Thermodynamic Modelling
Theriak-Domino and pMELTS are both thermodynamic modeling tools, but they are not interchangeable and have important differences. Theriak-Domino is primarily optimized for subsolidus phase equilibria. Although recent studies have explored its application to melting processes, this use remains under development and should therefore be applied with caution (see comment below regarding errors in the Theriak-Domino database).
An additional limitation is that Theriak-Domino does not calculate trace-element behavior. The modeling presented in this study focuses exclusively on major elements, yet trace elements are critical for evaluating melting, fractional crystallization, and magma mixing processes, particularly when comparing modeled results with natural rock compositions that have undergone these multiple evolutionary steps. While trace-element calculations cannot be performed directly within Theriak-Domino, they can be modeled using phase proportions derived from pMELTS. The manuscript would be significantly strengthened by including a comparison between observed and modeled trace-element compositions and by explicitly addressing expected trace-element behavior, especially where modeled and measured compositions diverge.
Section 1.7.2 introduces an additional modeling software, but the rationale for its use and its relationship to the other modeling approaches are not clearly explained. This lack of clarity makes it difficult to assess the internal consistency of the methodology.
More generally, each modeling approach carries its own assumptions and limitations. The use of multiple software packages and calculation strategies within a single workflow can substantially increase uncertainty, particularly when outputs from one model are used as inputs or constraints for another. Without a clear justification for each modeling choice and an explicit discussion of uncertainty propagation across the workflow, it is difficult to any reader to evaluate and trust the robustness of the final interpretations.

Given the large number of assumptions underlying the proposed model, methodological consistency is especially important. It is unclear for me why magma mixing and fractional crystallization were initially modeled using empirical approaches (e.g., the fractional melting equation of Shaw, 1970) rather than thermodynamic calculations using pMELTS (Ghiorso et al., 2002), which would provide a more modern and rigorous framework for evaluating phase relations and component behavior. Sections 1.2–1.6 could potentially be addressed using thermodynamic modeling, as was done in later sections, allowing element distributions to be evaluated within a phase-controlled framework using appropriate partition coefficients. This

would result in a more internally consistent and robust interpretation.

Specific comments about each software:
a) Theriak Domino:
The manuscript states that the thermodynamic dataset dS733 was used. This database is not known, and if it was indeed employed, its source must be clearly specified. However, the reference cited in the text indicates the use of the well-known ds633 database. If this discrepancy is simply a typo, it should be corrected consistently throughout the manuscript.
If ds633 is the database actually used, the following issue must be addressed. An algebraic error in the formulation of the thermodynamic model for silicate melt in Holland et al. (2018) was identified, and Green et al. (2025) published a corrigendum for this dataset (DOI: 10.1093/petrology/egy048). Because this melt model forms a central component of the thermodynamic calculations employed in this study, the modeling should be re-run using the corrected database. The presence of a known error in the original formulation directly affects the reliability of the results presented here.

Moreover, the corrections and examples discussed in Green et al. (2025) are primarily applied to anhydrous peridotite systems. The applicability of this database to hydrous peridotite melting, which is fundamental to the present study, therefore remains uncertain and should be explicitly addressed. As noted by Green et al. (2025), “melting relations in peridotite systems are the subject of ongoing model development.” Consequently, the use of this database for hydrous peridotite modeling is itself non-trivial and requires careful justification and discussion of associated uncertainties.

b) pMELTS
I note that several phases appear to have been suppressed in the modeling files. If this is the case, the rationale for phase suppression and its implications for the modeled melts must be explicitly discussed.
In addition, potassium (K) does not appear to have been treated as a major element in the melt modeling, where it is instead considered only as a trace element, whereas K is treated as a major element in the Theriak-Domino calculations. Input data and element classifications should be consistent across all modeling approaches, and any deviations should be clearly justified since it adds more uncertainty.
Furthermore, the manuscript does not explain why a single mantle composition from Palme and O’Neill was used in combination with major-element compositions from Walter (2013). As I understand it, the latter represents a modeled composition resulting from melt extraction, rather than a primitive mantle composition. Consequently, the modeling workflow appears to be based on a model-derived starting composition rather than a commonly accepted mantle reference composition. This choice requires clear justification, as it directly affects the validity and interpretation of the results.
In summary, I recommend the following revisions to improve the robustness of the manuscript:
1) Petrography
It is essential to include petrographic documentation, with representative thin-section photomicrographs, to demonstrate that the studied rocks are indeed pristine and preserve primary igneous mineral assemblages and textures.
2) Methodological consistency:
I strongly recommend adopting a more methodologically consistent modeling approach. At least four different software packages, each based on different calculation methods and assumptions, are currently used. Where possible, calculations should be carried out within a single thermodynamic framework rather than being distributed among multiple empirical and thermodynamic approaches. This would reduce uncertainty propagation and significantly improve internal consistency, reproducibility, and clarity.
For example, the calculations presented in Sections 1.2–1.6 could be revisited and performed using MELTS-based software, as done in later sections. This would also allow direct comparison of thermodynamically modeled trace-element compositions with measured rock compositions.
3) Theriak-Domino modeling and database transparency:
I understand that Theriak-Domino was used for calculations in pressure ranges where the parameters in alphaMELTS are considered less reliable (i.e., at elevated pressures). One way to address this issue would be to run both approaches and compare the results. If the results are similar, pMELTS outputs could then be used to assess trace-element behavior.
If the authors decide to retain Theriak-Domino melting calculations, it is necessary to re-run the models using the corrected thermodynamic database, given the documented errors in the previously used version, and to explicitly discuss the limitations of applying this model to hydrous peridotitic systems. In addition, I suggest making the thermodynamic database available and clearly indicating which phases were activated or suppressed.

Version 1:

Reviewer comments:

Reviewer #3

(Remarks to the Author)
Modern arc-like water content in the source of 3.1-billion-year-old volcanic rocks

Eric D. Vandenburg et al.

This was the second time I’m reading this manuscript, and I believe the revision has been going on for longer. I appreciate the authors reading and replying to all my comments and suggestions. The manuscript has now all calculations on the same software making it more consistent. I’m not familiar with the software that was used but I was able to follow the steps using the supplementary files. A good number of my suggestions did not make into the manuscript, but I understand that’s the

authors choice.
However, my main concern is still about the LILE and sample preservation. The authors and I have opposing views here since a good number of geochemical plots can have varying interpretations. For example, figure R2 displays the lack of covariation between Rb, Cs, Ba and MgO of the Whundo calc-alkaline basalts and modern calc-alkaline basalt. There is no statistical trend on this figure given the low R2 values, and assuming a similarity is simply a false premise. Instead of replying and debating the interpretation of each plot, I'd rather raise the point that geochemistry is extremely powerful tool, but there are limitations on what it can do alone. Hence the need to include the petrography. Exactly the qualitative aspect showing that the samples contain chlorite and sericite, needs to be on the main text. Figure R5 is ready and could be added to the supplements. With this information, the reader can approach the manuscript and be clear what is the alteration. Whether it is an isochemical system will be up to reader to make up their mind.
In my opinion, the rocks are relevant enough to be worth publishing. The main conclusions are highly debatable but at this point this discussion should be made public after minor revisions just to include the petrography text and supplementary figure.

Open Access This Peer Review File is licensed under a Creative Commons Attribution 4.0 International License, which permits use, sharing, adaptation, distribution and reproduction in any medium or format, as long as you give appropriate credit to the original author(s) and the source, provide a link to the Creative Commons license, and indicate if changes were made.
In cases where reviewers are anonymous, credit should be given to 'Anonymous Referee' and the source.
The images or other third party material in this Peer Review File are included in the article's Creative Commons license, unless indicated otherwise in a credit line to the material. If material is not included in the article's Creative Commons license and your intended use is not permitted by statutory regulation or exceeds the permitted use, you will need to obtain permission directly from the copyright holder.
To view a copy of this license, visit https://creativecommons.org/licenses/by/4.0/

Responses to reviewer feedback on revised manuscript of
Modern arc-like water content in the source of 3.1-billion-year-old volcanic rocks

Eric D. Vandenburg *et al.*

We thank the four reviewers for taking the time to review our paper and providing us with excellent feedback on the manuscript. We have revised the manuscript to reflect these suggestions. Below, we respond to the comments and edits suggested by the reviewers and outline how we have strengthened the manuscript in response to their feedback. Reviewer feedback is highlighted in cyan.

As recommended by Reviewer 1, we have developed an additional alternative model for Whundo boninite generation, provided in Supplementary Figure 8. This model yields similar results to the main model.

As recommended by Reviewer 4, we have contained all thermodynamic modelling within a single software package, in this case MAGEMin. To increase robustness of our methodology, we have also incorporated thermodynamic modelling into the trace element modelling of stage 1 mantle depletion, by using the outputs simulating decompression melting along mantle potential temperature adiabats to determine phase relations and mineral-melt partition coefficients during partial melting. This is much more realistic than the method in the previous manuscript version, which assumed fixed melting reactions and partition coefficients, and further reinforces the findings of our study. We have also added a section to the *Supplementary Methods* that discusses the limitations of our modelling.

We have clarified and elucidated on points in the main text that reviewers highlighted as potential sources of confusion to the reader. In addition, we have updated the *Methods* and *Supplementary Methods* sections to provide further information about the thermodynamic modelling used in this study and its rationale. Furthermore, we have updated geochemical and thermodynamic modelling workflow figures to the Supplementary Figures to further assist the reader in understanding our methodology.

We have included all results, input code and output data, and data used to construct figures in the Supplementary Tables, Supplementary Data, and Source Data to ensure full transparency and reproducibility of our findings. Even though these fixtures are somewhat technical, they are a necessary addition to fully justify our claim of very high water content in the mantle source of the melts – a feature otherwise only observed in modern convergent margins.

The key message here: Water-rich fluxed melting, similar to arcs with all its implications, was operating in the Archean, yet without any geodynamic resemblance to modern subduction zones.

Responses to feedback from Reviewer #1 (Hugh Rollinson):

Review of Eric D. Vandenburg et al.
Modern arc-like water content in the source of 3.1-billion-year-old volcanic rocks
Submitted to Nature Comms

This is now the third time I have seen this ms and it has evolved considerably in the revision process. In their response, the authors have addressed my previous comments and made appropriate changes to their ms, which in my view are adequate.

In essence the problem is how to create an enriched mantle in the early Earth before, perhaps, the advent of subduction. In the modern Earth this would normally be done through the introduction of a fluid-mobile element enriched fluid into the mantle to create an enriched source and trigger melting. The dilemma is how did this happen in the Archaean.

The authors have now abandoned a subduction model and are advocating a drip-duction model, which could work – its fashionable. The geodynamic model is supported by a complex array of geochemical calculations modelling the observed rock compositions.

I have two comments.

Comment: 1. The proposed geochemical model is probably not unique – in other words there may be other ways of modelling the observed rock compositions. I would therefore urge some caution in the text indicating that other solutions may be forthcoming and so whilst this model satisfies the geochemical constraints it may not be the only solution.

Response: We agree with the reviewer to some degree, and we have updated the manuscript by adding an alternative model for the boninites as per their suggestion. As evidenced by the specifics of our revised model and comparison to the old model in the previous manuscript version, there is certainly some wiggle room with respect to the exact parameters, proportions and amounts. However, we stress that several key conditions need to be met, irrespective of the model, to produce Whundo boninites. These include:

- A clinopyroxene-poor to -exhausted harzburgitic mantle source.
- A highly refractory component in the source (comprising either the majority of the source or its entirety) that underwent a prior high-degree depletion event deep in the garnet stability field; this is necessary to generate the concave-up

REE patterns with an inflection at Dy. This concave-up REE pattern cannot be explained by the involvement of amphibole in the petrogenesis of the boninites, based on mineral-melt partitioning in amphibole.

- Re-enrichment of the depleted source by a Th-U-LILE-LREE-rich, Nb-Ta-depleted metasomatic agent, based on trace element systematics and modelling results. This precludes a typically "intraplate" origin solely involving melt re-enrichment of an ultra-depleted source (c.f., Mahiniki [Golowin *et al.*, 2017] or the Rollinson [2022] petrogenetic model for Isua boninites). Given these observations, the metasomatic agent is likely to be hydrous.

In addition to adding an alternative model, we have added a paragraph addressing non-uniqueness and the key takeaways regardless of the specific model.

Changes made in response to comment:

Main Text:

Lines 299-314: added: "These melting curves also illustrate that the specifics of this model are, to some degree, non-unique, as decreasing the amount of the URH component can be compensated by decreasing the degree of melting to explain individual samples (Fig. 2c). Indeed, it is important to stress that other models are possible. For example, two-stage melt modelling can also reproduce the patterns observed in the I-boninites if a melt-free refractory harzburgitic solid, produced by high degree melting (past garnet exhaustion) of primitive mantle along a 1,610°C T_P adiabat, is metasomatized by a small amount (< 4 wt.%) of primitive tholeiitic melt (Supplementary Fig. 8a). However, it is evident that several key conditions need to be met, irrespective of the model, to produce the Whundo boninites. These include: (i) a clinopyroxene-poor to exhausted harzburgitic mantle source; (ii) a highly refractory component in the mantle source (comprising either the majority of the source or its entirety) that underwent a prior high-degree depletion event past garnet exhaustion deep in the garnet stability field; and (iii) re-enrichment of the depleted mantle source by a Th-U-LILE-LREE-rich, Nb-Ta- depleted metasomatic agent (Fig. 4c, Supplementary Fig. 8a).

Lines 338-340: added: "Using the composition of the harzburgite from the alternative boninite model produces a similar result (0.85 to 1.57 wt.% H_2O; Supplementary Fig. 8b)."

Main Text Figures:

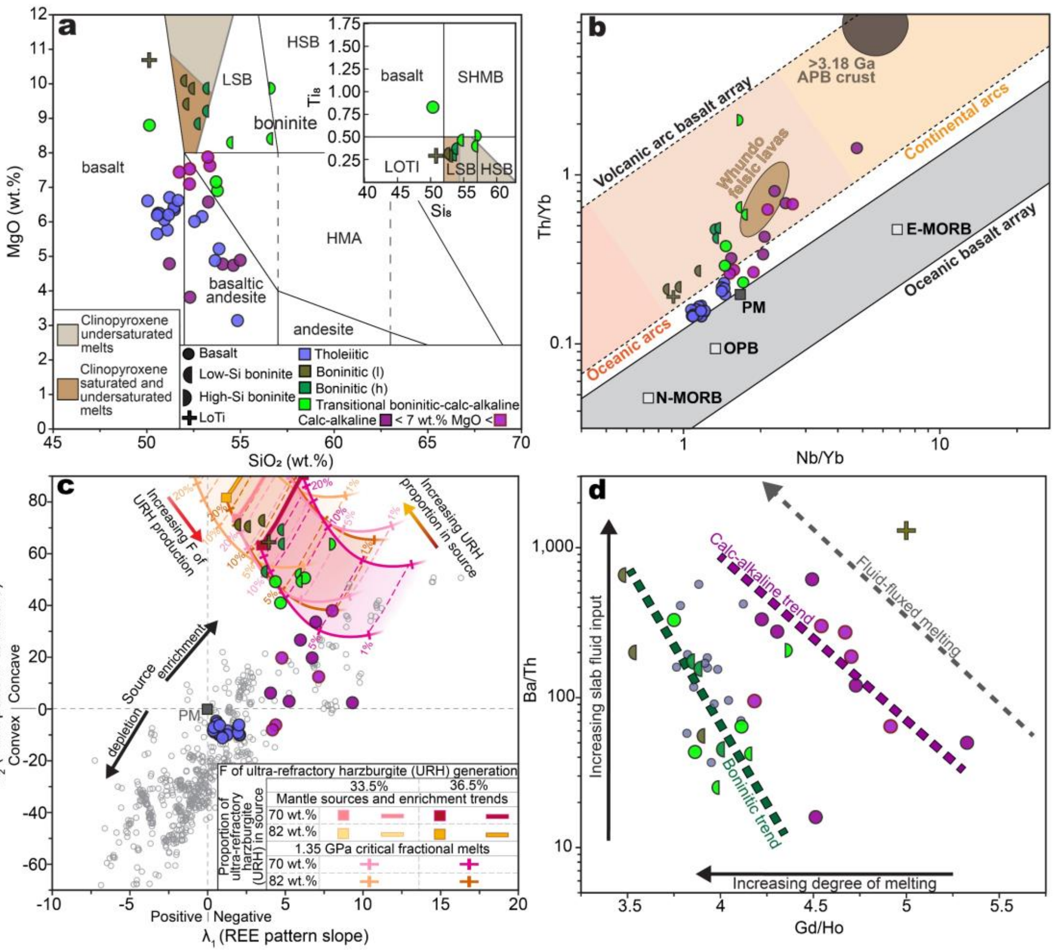

UPDATED: Figure 2: constructed four different λ melting curves along enrichment trends to demonstrate how varying the proportion of ultra-refractory harzburgite (URH) component in the hybrid mantle source and the degree of melting at which this URH component was produced, can explain the trends observed boninitic lavas. This also demonstrates the opposing impacts of increasing the degree of melting of the URH production vs increasing the proportion of the URH component, which results in some degree of non-uniqueness.

Supplementary Figures:

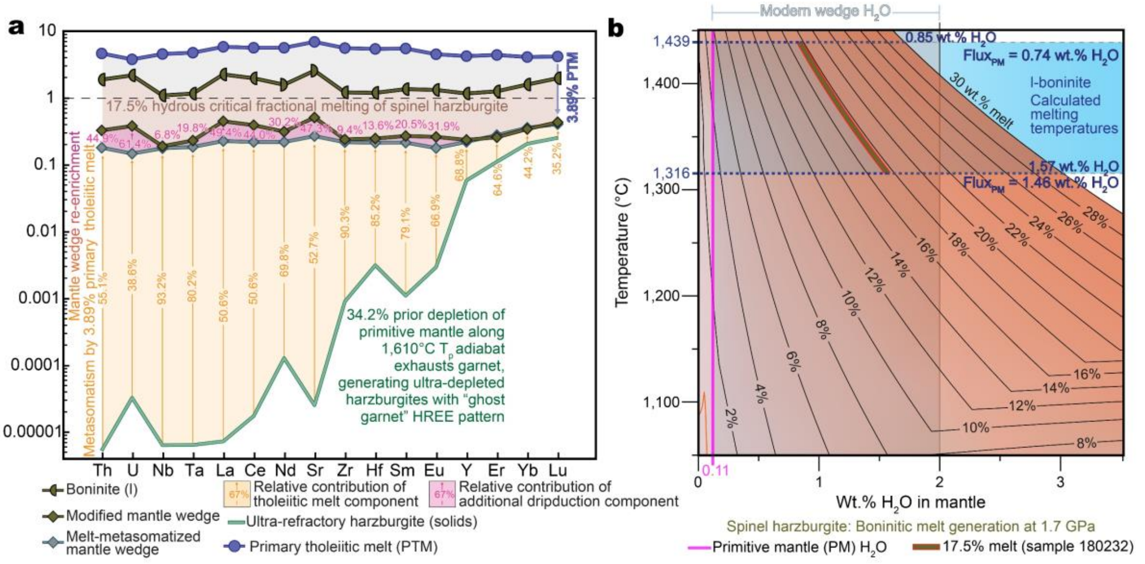

NEW: **Supplementary Figure 8:**

Comment: 2. As indicated by one of the other reviewers I am not persuaded that the model makes much sense in terms of the rather complex and varied stratigraphy. Unlike the example of the Oman ophiolite cited, where the different lava sequences have a sense of uniformity and stratigraphic progression, this is much less clear in this study.

Response: We thank the reviewer for raising this point. Part of the reason why we chose to compare Whundo to localities such as the Oman and Troodos ophiolites and Izu-Bonin-Mariana forearc is that (a) these are well-known localities and (b) we wanted to demonstrate the lack of resemblance to Phanerozoic subduction initiation locales. There are likely to be two factors at play regarding Whundo that can explain this purported lack of uniformity and stratigraphic progression.

The first factor relates to the relative position of the Whundo group within the dripduction/subduction-like setting. As the reviewer pointed out, we make comparisons between lavas from Whundo and those from Phanerozoic settings. Two of the locales we use for comparison, Troodos and Oman, are preserved within ophiolites that record subduction initiation in a forearc setting (e.g., Dilek and Furnes, 2014; Guilmette *et al.*, 2018; Lehmann *et al.*, 2025; Ribeiro *et al.*, 2023). From studies of these ophiolites as well as of other subduction initiation locales, such as the IBM forearc, we know that these subduction initiation settings tend to have a characteristic stratigraphy (albeit with some variability), with a basal layer of forearc basalts, overlain by boninites, which are in turn overlain by high-Mg andesites, and finally capped by arc tholeiites and calc-alkaline basalts (e.g., Belgrano *et al.*, 2019; Ishizuka *et al.*, 2011; Pearce and Reagan, 2019). This lack of stratigraphic resemblance to Whundo indeed suggests that Whundo does not represent an Archean analogue of forearc subduction initiation. However, there are other parts of subduction systems in space and time where boninites can occur that have much more random stratigraphy. For example, the Yavuna Group of Fiji contains interbedded boninitic, tholeiitic and calc-alkaline lavas erupted contemporaneously during the transition from the protoarc to first arc stages of subduction initiation away from the plate boundary (e.g., Pearce and Reagan, 2019; Todd *et al.*, 2012). Similarly, Pearce and Reagan (2019) suggested that the Whundo boninites best resemble those from modern arc-basin settings, which do not exhibit a diagnostic

stratigraphic progression. Thus, all that can really be said concerning this point is that the Whundo Group did not erupt in a forearc-analogous setting.

Another possibility has to do with the mechanics of dripduction itself. The down-going slab would be considerably more plastic compared to modern subduction zones, owing to the rheological differences imparted by a hotter Earth (e.g., van Hunen and van den Berg, 2008), such that it would be prone to frequent breakoff in the form of dripping. This would temporarily pause the arc-like magmatism, as the dripduction zone re-nucleates and re-initiates (e.g., Moyen and van Hunen, 2012; Sizova *et al*., 2015). Understanding the stratigraphic manifestations of this is largely beyond the scope of this study and is currently under investigation, but we note that there is indeed some cyclicity in the magmas, as evidenced by the stratigraphic column in Figure 1b and geochronology, Hf isotopes and trace elements in zircons that will be presented in Vandenburg et al. (in prep.).

That said, we agree that we should be a bit more explicit that the Whundo Group isn't necessarily analogous to a subduction initiation forearc setting and emphasize that the final two panels in Fig. 6 are not necessarily sequential and can occur interchangeably. We have made appropriate changes to the manuscript.

Changes made in response to comment:

Main Text:

Lines 139-141: added information about the boninites' closest Phanerozoic analogues, based on previous work by Pearce and Reagan (2019): "Previous work based on their major element and Nb/Yb-Th/Yb systematics have shown that low-Si arc-basin boninites may be the Whundo boninites' closest Phanerozoic analogues[25]."

Lines 411-424: addressed the stratigraphic variability and lack of resemblance to forearcs and ophiolites that record subduction initiation, and how this can be reconciled with other parts of a convergent margin system: "As evidenced by the varied, albeit weakly cyclic stratigraphy of the Whundo Group (Fig. 1b), it is important to recognize that these primitive lavas, particularly the calc-alkaline basalts, boninites and their hybrids, were erupted diachronously. This contrasts heavily with the typical uniformity and stratigraphic progression from tholeiite to boninite and finally to calc-alkaline BADR lavas that is characteristic of subduction initiation recorded in Phanerozoic forearcs and ophiolites[24,25]. Thus, given the available evidence, including the similarities between the Whundo boninites and their modern arc-basin counterparts[25], Whundo is unlikely to be an Archean analogue of subduction initiation in a forearc setting. Understanding this stratigraphic variability and cyclicity is beyond the scope of this study, but these observations remain reconcilable with Whundo as an analogue of another portion of a convergent margin setting, such as an arc-

front or arc-basin setting, where different lavas erupt diachronously[25,28], even during subduction initiation[63]."

Main Text Figures:

UPDATED CAPTION: **Figure 6:** added **"Note that ci and cii are temporally interchangeable, perhaps even contemporaneous**."

I feel inclined to say let this discussion now go into the public domain and see how other workers respond.

Hugh Rollinson
University of Derby
27/11/25

Responses to feedback from Reviewer #2:

Review of Vandenburg et al.
Modern arc-like water content in the source of 3.1-billion-year old volcanic rocks
Submitted to [*REDACTED*]

Comment: This manuscript is a resubmission that several others have reviewed, and the revisions from the previous review are substantial. The manuscript and supplementary files present a detailed study of 3.1 Ga volcanic rocks of the Whundo Group in the Pilbara Craton, Australia. The authors use these rocks to assess the possibility of subduction before 2.8 Ga. The focus point of this manuscript is that hydrous melting initiates early subduction, or dripduction, at 3.1 Ga. They conclude that, despite operating under different conditions, the system is similar to modern volcanic arc systems. Overall, this manuscript is well written and detailed, and it presents an argument supported by the data. I hope my suggestions help the authors and look forward to seeing this manuscript in publication.

Response: Thank you for the kind words.

Comments:
Comment: 1. Chemical Alteration and Weathering of Samples. I was specifically asked to assess the issue of alteration in these rocks. I used the author's data to determine alteration indices from several methods designed to examine chemical weathering and hydrothermal alteration of igneous rocks, including the Chemical Alteration Index (Nesbitt and Young, 1982), the chlorite-carbonate-pyrite index (CCPI), and the Ishikawa Alteration Index (AI; Ishikawa et al., 1976). The full data set includes several samples that exhibit evidence of chemical weathering and hydrothermal alteration, but these are the same samples that were filtered out based on LOI >5 wt%. The filtered dataset used by the authors for the models shows indices that suggest minimal alteration and chemical weathering. The authors made a similar statement in response to reviewer #1 and reached a similar conclusion about the level of alteration these indices suggest.

Compared to modern arc volcanic rocks, the CCPI Index suggests some Mg-Fe chlorite alteration, with an average index of 87% and a minimum of 75%. Modern unaltered arc basalts range between a CCPI index of 70-85%, and the authors suggest that the tholeiites do not resemble other early Earth subduction-related rocks and therefore could be formed in a divergent setting, which could result in higher initial FeO and MgO contents. Comparing this index to LILE element contents within the filtered sample shows a slight correlation with the Ishikawa AI. Still, the overall variability of elements such as Rb, at only 40 ppm, is well within that observed in modern arc volcanic systems. The same is true of other LILE elements modeled, as illustrated by minimal variation in Ba/Sr ratio and Rb/Sr ratio.

Overall, I believe the authors appropriately filtered the data and restricted the dataset to unaltered samples for their interpretations. While I do believe there is some

amount of alteration/metamorphism that must have occurred in rocks >3 Ga, it appears it is minimal in the selected samples and do not affect the overall interpretation.

Response: We thank the reviewer for verifying the robustness of our alteration screening methodology and LILE systematics; we agree that alteration was minimal in the selected samples. To further bolster our claims, we have updated Supplementary Figure 2 to include similar assessments for Cs and Ba.

Changes made in response to comment:

Supplementary Figures:

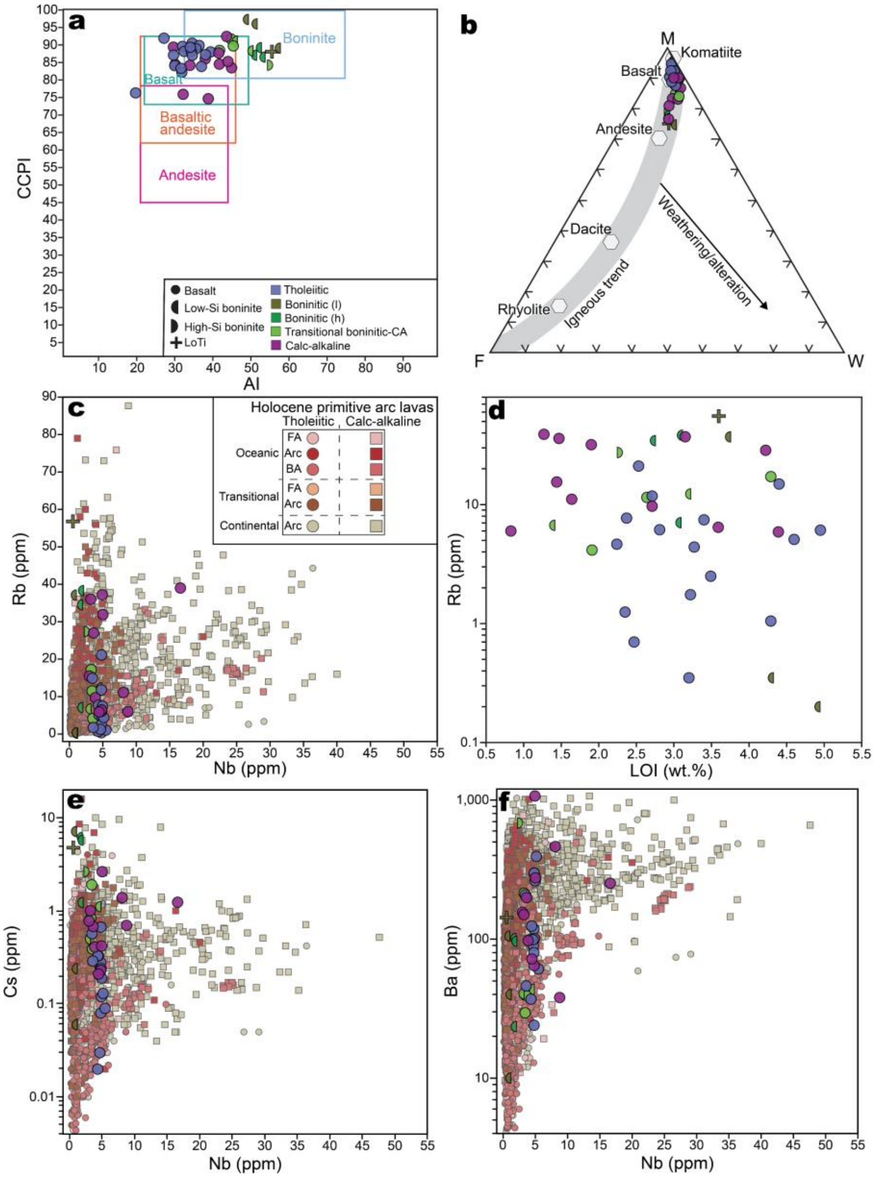

UPDATED: Supplementary Figure 2: added Cs vs Nb and Ba vs Nb plots, changed displayed Holocene arc lavas to unaltered basalts and boninites with MgO contents within the range of the samples presented in this study, rather than just primitive lavas.

Comment: 2. Thermodynamic Model and Equations. I thank the authors for the detailed workflow for deriving Equation #4 and for the thermodynamic model. These allowed me to easily recreate the models and mantle sources used for the interpretations and conclusions.

Response: We're glad that the workflows provided have been useful in reproducing our results.

No changes made in response to comment.

Comment: The evidence for fluid flux melting is difficult to follow, and the text takes a long time to develop the concept. The figures highlighting this are presented in the supplementary material, with the need to go between Figure 2 and Supplementary Figure 7. There is a figure in the reviewer responses, Figure R2, that makes this argument clear and directly links it to the argument for fluid-flux melting versus other melting processes.

Response: We thank the reviewer for the feedback and hope that the evidence is easier to follow in the revised manuscript.

We're unsure which reviewer response figure the reviewer is referring to, as Figure R2 in the response to the previous round of reviews was a diagram demonstrating the lack of Mg mobility in the Whundo lavas. Perhaps they are referring to Figure R1? In any case, we have added the panels from the latter to a new Supplementary Figure 7. While we cannot move them into Figure 2 because of the poor use of space that a five-panel figure would entail in the main text, we hope that their addition as a supplementary figure is sufficient.

Changes made in response to comment:

Main Text:

Lines 205-207: The assertion of fluxed melting is further supported by the trends observed on plots of K_2O vs K_2O/Na_2O and Yb vs Zr, which suggest a fluid-, rather than melt-dominated "slab" component in the source of most Whundo lavas (Supplementary Fig. 7a-b).

Supplementary Figures:

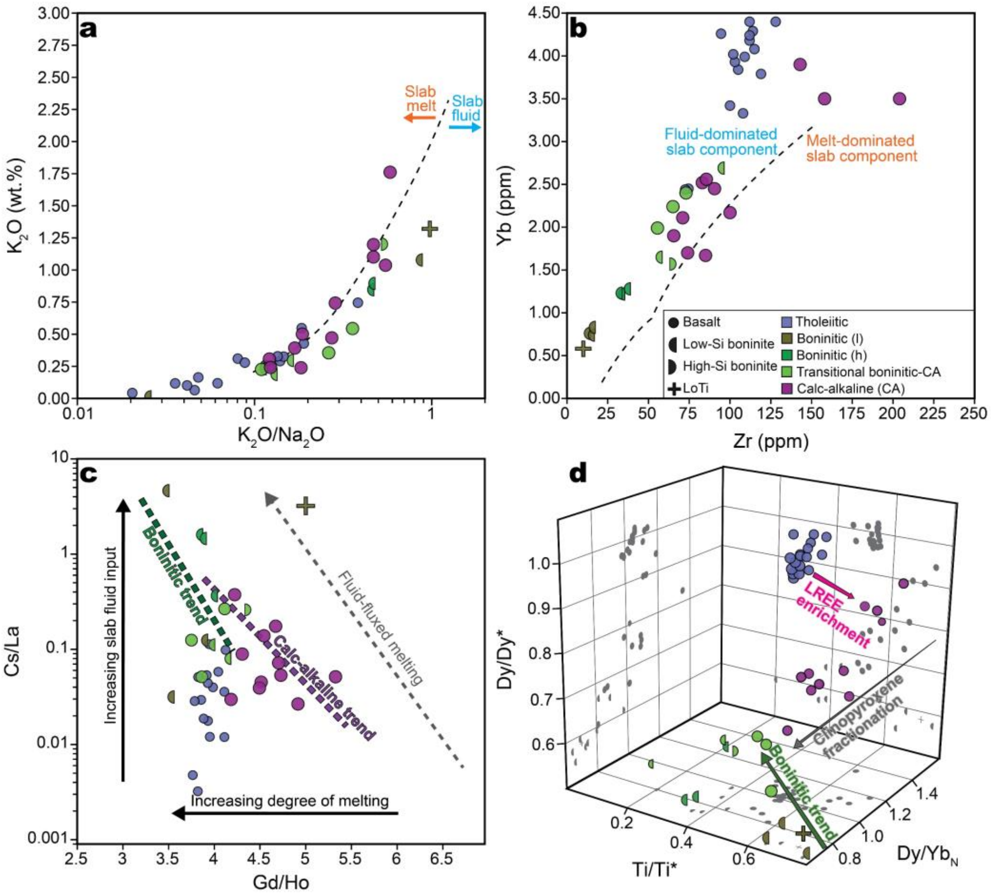

<u>NEW:</u> Supplementary Figure 7 (merged Supplementary Figures 7 and 8 from original manuscript with Figure R1 from previous round of reviews): added plots of K_2O vs K_2O/Na_2O (a) and Yb vs Zr (b) after Schmidt and Jagoutz (2017), differentiating between melt- vs fluid-dominated slab inputs.

<u>Comment:</u> The authors also use PM-normalized REE shape parameters (lines 302-317) to interpret mantle-source melting. This is the only are in the modelling that needs more description. For example, the authors state (line 304-307): “Our mantle melting models in REE shape (λ1-λ2) space indicate the Whundo boninites were derived from 6-17.5% melting of a clinopyroxene-poor harzburgitic mantle source, itself derived from a prior depletion event that melted a source to a point past garnet exhaustion.” They do not provide ranges to be of the λ1-λ2 space that are expected for garnet-stable, versus garnet-residual sources, or the differences between harzburgite source shape versus lherzolite. This is, overall, a minor point, but when the authors are trying to argue for “ghost garnet” signatures (or lack thereof) by pre-depletion of garnet before mantle melting, having some knowledge of these ranges could make the argument easier to understand and could reduce the inference that the data is over-interpreted.

<u>Response:</u> To clarify for the reviewer, the melting curves in the λ diagram of Figure 2 are informed by the geochemical and thermodynamic modelling that we have

conducted as part of this study, and the detailed methodology is provided in the *Supplementary Methods.* The interesting thing about melting curves in λ_1-λ_2 space is that they are petrogenetic process vectors that describe the change in average REE slope and curvature between the REE patterns of the source and melt. Therefore, changing the source's λ_1-λ_2 values will not change the shape of the melting curve, merely changing the anchor point of the curve to the new source's λ_1-λ_2 values. What does change the shape of the curve, however, is the mineral modes, melting reactions and REE partition coefficients in the source. We have attached Figure R1, which shows the difference in melting curves between a clinopyroxene-poor spinel harzburgite and a slightly-depleted spinel lherzolite, as differences in source mineralogy (other than garnet vs spinel facies) are not explored in O'Neill (2016).

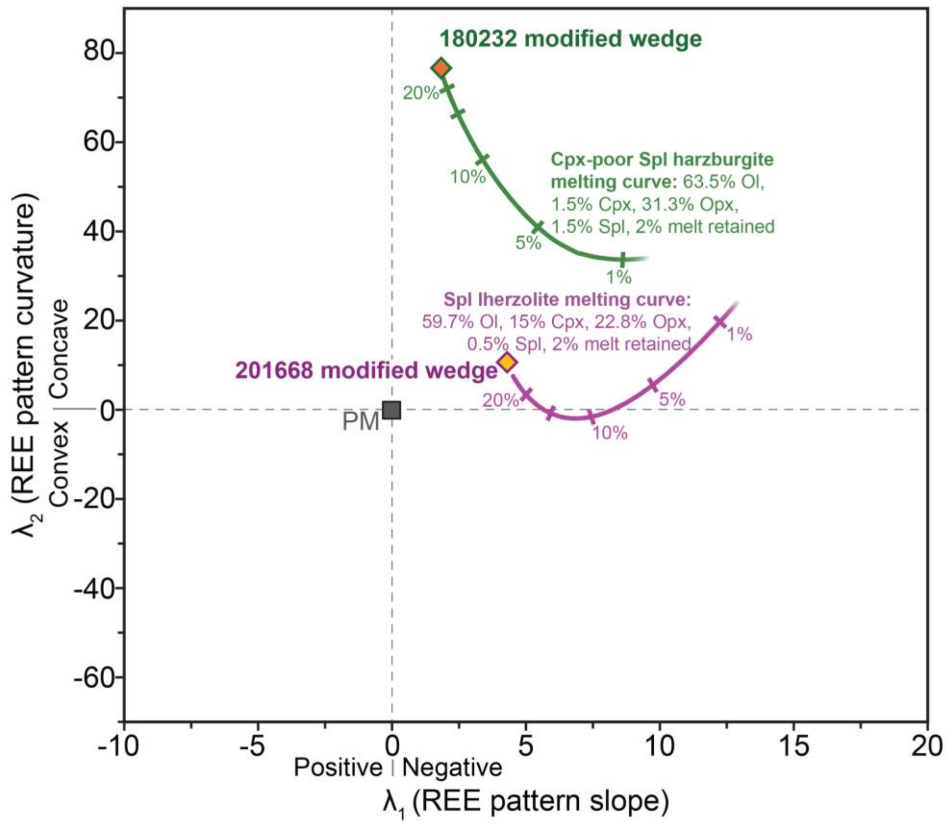

Figure R1. Effects of source mineralogy on λ_1-λ_2 melting curves in the spinel stability field. Melting curves for clinopyroxene-poor spinel harzburgite and spinel lherzolite, assuming modified mantle sources of boninite 180232 and calc-alkaline basalt 201668, respectively. Note how the curve becomes less arcuate and the inflection point changes between relatively clinopyroxene-rich lithologies, such as spinel lherzolite and clinopyroxene-poor lithologies, such as harzburgite.

The λ_1-λ_2 ranges indicative of garnet-stable (residual?) vs garnet-exhausted sources are not as straightforward as they seem, as the regions for that will differ depending

on what the source is (i.e., DMM vs primitive mantle). Furthermore, in contrast to our old model, which relied on an entirely residual source, our new model for boninite formation relies on a modified source that is comprised of small proportions of slightly depleted spinel lherzolite, admixed to ultra-refractory harzburgite (URH) that was depleted in the garnet stability field, so there is the added aspect of mixing.

We have modified the λ_1-λ_2 in Figure 2 to illustrate how variations in source parameters, such as the proportion of the URH component in the mixed mantle source, the degree of melting at which this URH component was produced (assuming the same T_p adiabat), and indirectly, the degree of dripduction-related source enrichment, affect the mantle source points and melting curves (with mineralogical variations resulting from these differing parameters informed by our thermodynamic modelling). The panel is already quite crowded, so we hope the reviewer will forgive us for not including the garnet-stable vs garnet-exhausted fields! We hope that the four melting curves we have incorporated into the figure illustrate the origins of some of the trends observed in the boninitic lavas.

Changes made in response to comment:

Main Text Figures:

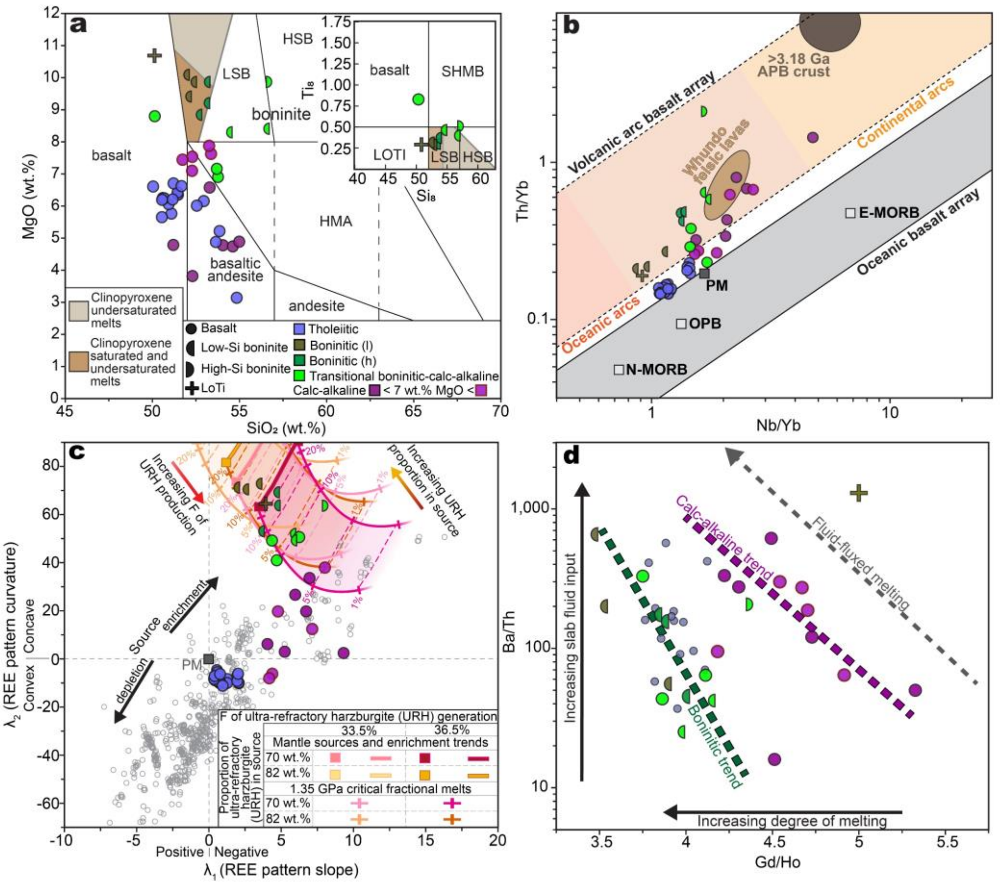

UPDATED: **Figure 2:** constructed four different λ melting curves along enrichment trends to demonstrate how varying the proportion of ultra-refractory harzburgite (URH) component in the hybrid mantle source and the degree of melting at which this URH component was produced, can explain the trends observed boninitic lavas. This also demonstrates the opposing impacts of increasing the degree of melting of the URH production vs increasing the proportion of the URH component, which results in some degree of non-uniqueness.

Responses to feedback from Reviewer #3:

General comments on Vandenburg et al.

“Modern arc-like water content in the source of 3.1-billion-year-old volcanic rocks” or “Earth's first primitive subduction occurred in the missing 2 majority of Archean crust”

This is the first time I’m reading this manuscript, but I understand it has already been through some rounds of revision. I suspect some of my comments might have been previously raised, but that happens when joining an on-going revision. The manuscript is polished and well-written presenting a series of samples with boninite signature, which is rightfully uncommon during the Archean and only known on a handful of locations. The identification and description of those rocks alone would be worth of publication – even considering the boninite like signature of the Whundo group was already mentioned by Smithies et al. (2005); However, the authors focused on an elaborate petrological model instead.

My major concern is the very basic assumption that the rocks are pristine and represent lavas, with REE, HFSE and LILE elements preserved. At the current state of the manuscript, I’m not convinced that is the case.

Comment: The REE and HFSE could likely represent the original signature, but expanding that to the LILE (K, Rb, Ba, Pb, Ce etc.) is much more difficult. I’ll use as an example the work by Shervais et al. (2021), reference 74. of the manuscript. Their work drilled 800 meters into the boninite type locality, and show that K, Rb, Ce are significantly altered with minor mobility on Pb, Ba, Sr. The lack of correlation with MgO is used as argument for mobility, and further conclusion about those elements are very limited. The lack of correlation with Rb presented on figure sup 2 c and b of the manuscript is similar to the lack of correlation found on boninites, but that doesn’t make the sample boninites nor show that their LILE are preserved. I find it incredibly difficult to understand that, for an Archean boninite that has been tectonized to at least some extent, that those elements are preserved. Even at low temperature sea-floor alteration, low-temperature metamorphism, or subaerial exposure those elements are known to be mobilized. Therefore, a major aspect of the conclusions should be reconsidered, and I believe the data is overinterpreted in those terms. Additionally, there are signs of contamination that needs to be addressed.

Response: We understand the reviewer’s concern on this point, but we disagree and think that there are some points worth clarifying. First, as we note in the supplementary information, Rb (and other LILE) variability is commonly observed within modern arcs (e.g., Mullen and McCallum, 2014; Wang *et al.*, 2025; Wanke *et al.*, 2019), even at the scale of a single stratovolcano, and is expected due to heterogeneous and time-variable slab inputs, distinct pulses of magma, and

plumbing layouts. Given that the Whundo Group spans approximately 20-30 Myr, sampled over an area of approximately 114 km^2, variations in Rb concentrations are to be expected. This is a key difference between the samples described here and those presented in Shervais *et al.* (2021), as samples from the latter only span a period of <2 Myr from three drill holes (which limits the potential number of eruptive centers that those lavas could be sourced from).

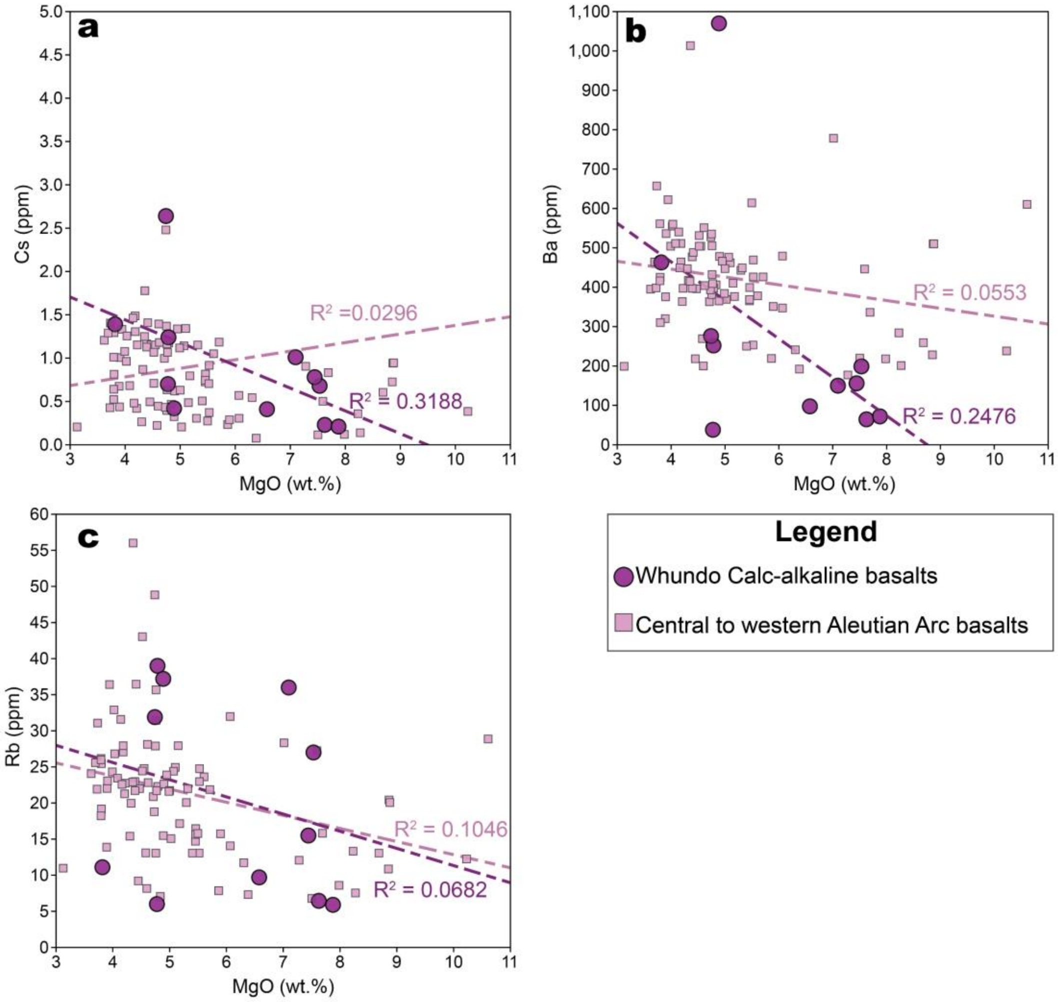

Figure R2. Covariations of Cs, Ba and Rb with MgO in the Whundo calc-alkaline basalts compared to modern calc-alkaline basalts from the central to western portions of the Aleutian Arc. Similar trends, of broadly increasing LILE concentrations with decreasing MgO, are observed in the Whundo calc-alkaline basalts compared to the Aleutian calc-alkaline basalts. Best-fit lines for each series are calculated using ordinary least-squares regression. Aleutians data is compiled from the GeoRoc and EarthChem databases.

Notably, each group does not share a cogenetic relationship with the others, so natural variations between groups might obscure some interpretations when plotted together. Although the boninite groups do not exhibit a sufficient range of MgO to be

able to evaluate mobility, the calc-alkaline basalts, which are just as central to the findings, do. In fact, Cs, Ba and Rb show correlations and variability with MgO comparable to those in modern volcanic arc basalts, as shown here in Figure R2 plotted with calc-alkaline basalts from the central to western portions of the Aleutian Arc. Similar patterns are observed with comparisons to other arcs, but we leave that as an exercise to the reader (which can be done using the data provided in Source Data Supplementary Figure 2).

Part of the beauty of science is that we don't have to all agree on everything. Within many fields, there have been opposing interpretations on key topics, even in modern times (one good example is the debate surrounding the causes of the differing oxidation states between island and continental arc lavas). We believe we have provided enough data to support our interpretation.

We note also that despite Rb being fully preserved in our opinion, any variability in this trace element beyond the reproducibility of the method (± 5%) does not alter our interpretation. We acknowledge, however, that more subtle variations may be possible, which have often led to disturbance in Rb-Sr isotope studies. Such variability, however, is too small to affect the modelling performed here, which we note also does not incorporate any of the most mobile LILE.

No changes made in response to comment.

Comment: The authors put a lot of effort to explain the models, which I appreciate. I think the calculations itself are reasonable, and only some minor changes would be needed. My main question is why the mixing and fractional crystallization (section 1.6) wasn't done using alphamelts, which technically is very similar to an AFC model. That would provide an entirely consistent thermodynamic model.

Response: The comment has largely been addressed and resolved due to the detailed changes in the modelling. In addition, the transitional series basalts and boninites are much less central to the findings of our study (as we focus on the endmembers). Now that AlphaMELTS is no longer used, the most consistent way of modelling this is through the equations.

No changes made in response to comment.

Comment: I've also added a collaborator as a co-reviewer who's very qualified in thermodynamic modelling who's providing a co-revision on the calculations.

Response: We thank the reviewer for doing this. The manuscript has certainly benefited from an additional set of eyes.

No changes made in response to comment.

Comment: My main suggestion for the manuscript is focusing on presenting a well-characterized description of the samples with petrography and outcrop photos – essentially circling back to some images that seem to be progressively vanishing with each revision. Archean boninites are rare and on it is own represent a significant finding and should be very carefully presented before elaborating on geochemical models. I'd suggest a major revision where all the considerations about the LILE are readdressed. Implications regarding the dripduction and REE and maybe HFSE are fine.

Response: We appreciate the reviewer's feedback here, but largely disagree and believe that this would be unnecessary. These aspects have already been investigated in Smithies *et al.* (2004, 2005) and touched upon to a lesser extent in Smithies *et al.* (2007, 2018). It is certainly possible for magmatic LILE to be preserved in Archean rocks; one such example would be the 3.33 Ga Commondale komatiites of the Kaapvaal Craton (Hoffmann and Wilson, 2017). Age forms no real criteria for gauging alteration. MORB that formed yesterday (geologically speaking) could be more altered than an Archean basalt due to hydrothermal activity. Alteration is not a guarantee of element mobility, as it can be isochemical, and hence, petrography is certainly not a silver bullet in addressing this. The gold standard in assessing chemical mobility in rocks, whether they be Archean arc-like basalts or modern MORB, is chemical parameters and proxies. We will address these concerns further in the detailed comments below, but it is also important to note that the majority of the key findings are unrelated to LILEs.

No changes made in response to comment.

Specific points:

Comment: Line 43 – "These samples provide a rare Archean analogue of modern arc volcanism, with fluid-mobile elements consistent with primary magmatic values." - I'd remove this sentence. It is a very bold statement for altered Archean rocks.

Maybe rephrase it like : "…provide rare Archean analogue for crustal recycling and mantle refertilization."

Response: We appreciate the reviewer's feedback here, although we do not fully agree. While bold, we do not make this statement without evidence, as provided in the subsequent sections of the manuscript and SI, in our references to Smithies *et al.* (2005), and in this review document. While the reviewer may not fully agree with us in this aspect, and they are, of course, free to do so, we believe we have provided enough evidence to support our claims. Other reviewers agree with us, and as we said, one of the beautiful things about science is that there are often opposing viewpoints. Therefore, we have not made any changes in response to this comment.

No changes made in response to comment.

Comment: Line 58 - "If widespread, such regional proto-subductions likely promoted volatile **exchange between mantle and ocean-atmosphere reservoirs during the Archean."** – If it was widespread there'd be more boninites across the Archean localities. AS made clear on the manuscript, it is not the case. I'd simply remove the sentence.

Response: As we state in the main manuscript, the reason why we don't see more boninites across Archean locales is due to preservation bias surrounding the settings in which these boninites would be expected to have been erupted, i.e., areas outside of the thick mafic-ultramafic plateaus that comprise most cratons. This is why we provide the caveat of "if widespread" in the abstract. We refer the reviewer to the following portions of the manuscript:

Main Text, lines 62-74: "*Only seven pre-2.8 Ga sites with putative proto-subduction origins have been identified in the volcanic record*[21]*, making up an insignificant proportion of the Archean crust. Crucially, all but one of these sites are of off-proto-cratonic plateau affinity, characterized by juvenile crust that developed without a pre-existing felsic basement. This is important as these sites do not contain abundant earlier TTG basements, which are often used to study subduction, require lower crustal delamination, and occur in slightly thicker crustal sections (>30 km)*[9,10]*. While the proto-cratonic plateau nuclei, which include TTG and form most of the preserved Archean crust, do not appear to record substantial dripduction processes, it is therefore possible that these processes occurred outside these plateaux, in the*

comparatively undifferentiated, thin crust now largely missing from the geological record. In rare cases, off-plateau juvenile crust is incorporated during craton assembly[22,23]*, helping to preserve isolated fragments within cratons.*"

Main Text, lines 395-398: "*Notably, the physicochemical nature of the resultant thin, evolved crust likely made it susceptible to later recycling by post-Archean Benioff-style subduction*[66]*, thus explaining its extreme scarcity in the geological record.*"

We believe we have made this point clear and, as such, have not made any changes in response to this comment.

No changes made in response to comment.

Comment: **LOI values on table 667710_0_data_set_11698630_t4trxx -** those are high, if those are pristine lavas which hydrated phase would hold this much water? Amphiboles would take up to 2 wt.% and micas about 4-5 wt.% being generous. I'm suspicious the 5% LOI is okay for some modern lavas where the formation environment is clear, but I don't think it's the case for your samples.

Response: We appreciate the reviewer's concern here. We applied the 5% LOI cutoff because of the upper threshold of H_2O contents in mafic arc lavas. The upper limit for magmas with > 5 wt.% MgO is 4.83 wt.% H_2O based on Kelley and Fischer (2025), but because we have a few samples with < 5 wt.% MgO, we have increased the threshold slightly. Because water is largely incompatible during fractional crystallization, even accounting for amphibole and micas, it will be retained in the melt phase. Petrographically, many of our samples are comprised of substantial amounts of devitrified glass, partially altered to minerals like chlorite (perhaps isochemically on account of the magmas' hydrous nature; see below). ~75% of our samples have LOI < 3.75 wt.%, so most of the screened samples are not even hitting this hard limit.

No changes made in response to comment.

Comment: **Fig. 2b -** The vertical trend on the plot could indicate a few things. An AFC trend would have a similar shape with end-member on each end of the curve. With your mixing model, I'd expect to see the calc-alkaline and the boninites on the same trend and one of the end-points with the calc-alkaline composition. I'm don't

think the mixing is clear from this plot, so maybe the vertical spread could have other causes such as the ones argued in Pearce (2008).

Response: We are unsure what the reviewer means here. The calc-alkaline basalts and boninites have different petrogeneses from one another, so there is no reason why these groups would fall along the same trend. It is evident from our study and Smithies *et al.* (2005, 2018) that the "dripduction" enrichment is quite variable, even within groups, thus explaining why we do not observe a linear array. We refer the reviewer to Figure 5 in Pearce (2008), which shows that the vertical trend is perfectly reconcilable with the effects of variable subduction inputs. As we have said before, variability is to be expected, given that the Whundo Group spans 20-30 Myr.

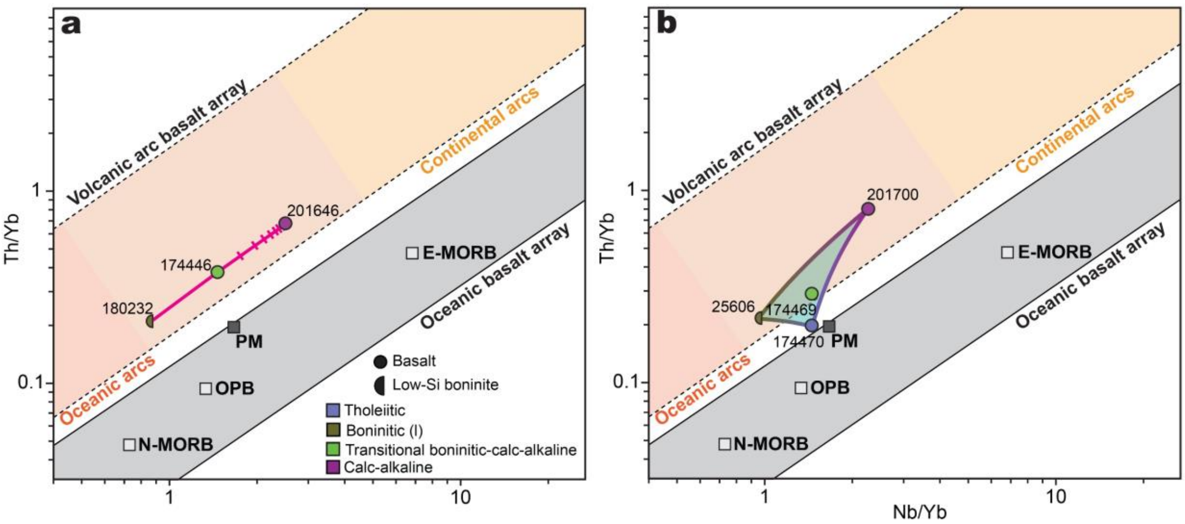

Figure R3. Possible mixing trends that explain the transitional boninitic-calc-alkaline basalts. Given the temporal variability with respect to enrichment, it is most useful to compare stratigraphically proximal samples to one another. (a) Simple binary magma mixing between stratigraphically proximal I-boninite sample 180232 and calc-alkaline basalt sample 201646 can explain the petrogenesis of transitional basalt sample 174446. Tick marks represent 10% intervals of calc-alkaline magmas. (b) Three-component magma mixing between stratigraphically proximal I-boninite sample 245606, calc-alkaline basalt sample 201700, and tholeiitic basalt sample 174440 can explain the petrogenesis of transitional basalt sample 174469. Fields and boundaries modified from Brown *et al.* (2024).

With respect to the transitional basalts, it is most germane to compare samples proximal to one another within the stratigraphy to account for temporal variability. We have done this in Figure R3. Simple binary mixing is very reasonable for transitional basalt sample 174446, as it falls along a line between the proximal I-boninite (180232) and calc-alkaline basalt (201646) samples (Fig. R3a). On the other hand, the systematics of transitional basalt sample 174469 suggest that it may have been

produced by three-component magma mixing between l-boninite (245606), calc-alkaline (201700) and tholeiitic (174470) magmas (Fig. R3b). This sample appears to be an anomaly because, on average, simple binary mixing can explain the trace element systematics as shown in Fig. 4d in the main text. This is similar to the petrogenesis of the "transitional calc-alkaline" basalts proposed by Smithies *et al.* (2005). Furthermore, transitional basalts at stratigraphically lower positions than the first appearance of tholeiitic basalts are unlikely to have a tholeiitic component. It is also plausible that some of the magmas involved in mixing did not erupt, or, given that this study comprises only two traverses, have not been sampled.

As we are primarily focused on the petrogeneses of the endmembers, rather than the transitional magmas, unravelling the sample-by-sample origins is better suited for further investigation in a future study.

No changes made in response to comment.

Comment: Line 211 **– "Unlike many Archean basalts, evidence for crustal contamination by evolved proto cratonic basement in the Whundo Group lavas is scarce."** – See my comment below on line 393, but I think your mixing model work just as well with assimilation giving there is no difference between the solid and liquid composition of the calc-alkaline basalts. The relative timing of your model with calc-alkaline forming first also opens this possibility.

Response: We remind the reviewer that the contaminant that we are discussing on line 211 is an evolved proto-cratonic basement (i.e., much older silicic, TTG-rich basement), for which there is no evidence of assimilation by Whundo Group lavas (nor should there be, as pre-Whundo silicic basement is likely absent from the Sholl Terrane). This can help us rule out an upwelling/plume-crust origin similar to the lavas of the Pilbara Supergroup in the East Pilbara Terrane (e.g., Brown *et al.*, 2024), which do not resemble the Whundo lavas.

As we address in the reviewers' later comment, calc-alkaline lavas did not necessarily form first, and we have removed any such indication to the contrary from the text. In the stratigraphy, the boninitic-calc alkaline hybrids appear first (those samples are andesitic, so they are not included in this study), so there is no evidence for an existing calc-alkaline edifice that would be requisite for assimilation (although

admittedly, the base of Whundo is an intrusive contact and therefore the true base is not known).

No changes made in response to comment.

Comment: Line 216 – **"Furthermore, the similarity between the Th/Yb-Nb/Yb systematics of the primitive and felsic26 lavas (Fig. 2b) suggests that they were not contaminated by ancient crust and therefore, erupted off-plateau."** – See the previous comment about figure 2b. There is a vertical array on your plot, and you should expand on that.

Response: While we understand and appreciate the reviewer's concern about crustal contamination, we note that the calc-alkaline basalts are the only series that exhibits a vertical trend. As we noted above, this is more reconcilable with variable degrees of enrichment from dripduction than with contamination of ancient crust. Some of the calc-alkaline basalts with the highest Th/Nb also have the highest MgO contents (Fig. 2b). This is the opposite of what would be expected if the vertical trend was produced by crustal contamination, given the thermodynamic limits on the amount of upper to middle crust that subalkaline basalts can assimilate before no longer being a basalt (e.g., Heinonen *et al.*, 2022). We have now elaborated upon this in the main text.

Changes made in response to comment:

Main Text, lines 179-189 (changes made in bold): "**Furthermore, the boninites and tholeiitic basalts exhibit array-parallel trends on a Th/Yb-Nb/Yb diagram, inconsistent with crustal contamination (Fig. 2b)**. **While the calc-alkaline basalts exhibit an oblique Th/Nb trend**, similarities between the Th/Yb-Nb/Yb systematics of the primitive and felsic**Error! Reference source not found.** lavas (Fig. 2b) suggest they were not contaminated by ancient crust and therefore, erupted off-plateau. While some degree of contamination by coeval felsic units cannot be ruled out, contamination-sensitive proxies lack covariations with increasing differentiation, indicating that contamination was not a major contributor to the incompatible element budgets of the primitive lavas (Supplementary Fig. 6). **Furthermore, some calc-alkaline basalts with the highest MgO also have the highest Th/Nb, thus reconcilable with variable mantle wedge enrichment (Fig. 2b).**"

Comment: Line 218 – **"While some degree of contamination by coeval felsic units cannot be ruled out, the lack of covariation of contamination-sensitive proxies with increasing differentiation indicates that contamination was not a major contributor to the incompatible element budgets of the primitive lavas**

(Supplementary Fig. 6).” – Your plots on Fig. sup. 6 show a shift towards the Whundo felsic lavas following your “calc-alkaline trend” purple arrow, which is exactly how a contamination would look like. Inclusive, some of the calc-alkaline are within the felsic field. Maybe there’s another trend on your plot? The differentiation trends using ratios on your axis should have a curved shaped, not linear.

Response: Based on thermodynamic considerations, it is improbable that crustal contamination is the reason why some of the calc-alkaline samples plot within the Whundo felsic lava field, considering that basalts can assimilate at most ~30% felsic crust before no longer being considered a basalt, which would need to be exceeded Instead, the fact that some of these lavas are well within the felsic field is likely a testament to the petrogenesis of the felsic lavas. Smithies *et al.* (2005) ascribed the origin of the felsic lavas to fractionation or partial melting of Whundo basalts and andesites, likely in a crustal hot zone. Admittedly, more work needs to be done on the felsic lavas, which will be presented with zircon data in Vandenburg *et al.* (in prep.). Furthermore, the calc-alkaline basalts do not exhibit curved trends on plots of La/Sm and Nb/Nb* vs Mg#, so they are indeed unlikely to result from differentiation. This is why we did not refer to the linear trends in Supplementary Fig. 6 as differentiation trends.

Another important consideration is the comparative positions of the samples within the Whundo felsic lava fields in Supplementary Fig. 6 to their positions on the Th/Yb-Nb/Yb diagram in Fig. 2b. For example, sample 201615 plots at the highest Nb/Yb of any calc-alkaline sample. This is not reconcilable with AFC processes due to interaction with Whundo felsic crust; this high Nb/Yb necessitates mantle wedge enrichment. As we say, “some degree of contamination” cannot be ruled out, but it is particularly insignificant in the higher Mg# lavas. Using a similar framework to that employed by Turner and Langmuir (2022), we can rule out assimilation as a contributor to variations within magmatic groups for samples with Mg# > 50 based on plots of incompatible elements vs Mg# (Fig. R4). Assimilation and crystallization will lower Mg# and raise incompatible element concentrations within magmatic groups. Th and La, which are very sensitive to assimilation, decrease slightly with decreasing Mg# within the calc-alkaline samples (Fig. R4b-c).

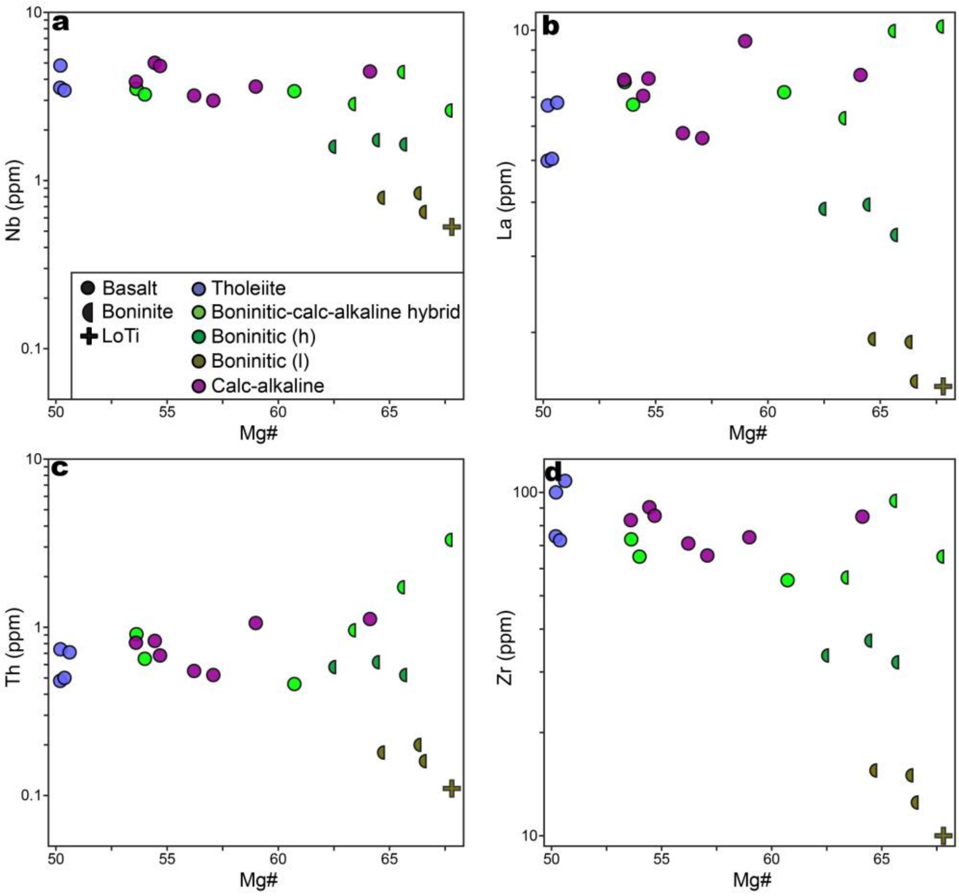

Figure R4. **Crustal contamination is irreconcilable with the incompatible element systematics of differentiation observed in Whundo primitive lavas.** Plots of Nb (a), La (b), Th (c) and Zr (d) versus Mg# as a proxy for differentiation for samples with Mg# >50.

No changes made in response to comment.

Comment: Line 229 – “The role of fluids/melts in the melting process can be studied by comparing **petrogenetic trace element ratios that track fluid enrichment (e.g., Pb/Ce, Cs/La, Ba/Th) to those that monitor the degree of melting (e.g., Sm/Lu, Gd/Ho).”** – Some of those elements are unlikely to be preserved, but you don’t need them to suggest flux melting. Those ratios might as well reflect some post emplacement alteration that you simply can’t tell. Even for modern lavas it is difficult dealing with those elements. However, just by having a boninite u-shaped REE, the mantle source needs to be previously depleted and followed by refertilization. How the refertilization happens is up for debate, where you suggested dripduction and that’s reasonable.

Response: We thank the reviewer for highlighting this point and believe that this is a testament to the robustness of the interpretations in this study. With respect to the ratios, our study expands upon the work of Smithies *et al.* (2005), which demonstrated that at least some of the LILE values remain faithful to magmatic

values. Such preservation is not entirely out of the ordinary; primary LILE values have been reported in the 3.3 Ga Commondale komatiites in the Kaapvaal Craton by Hoffmann and Wilson (2017), which is notably 200 Myr older than Whundo.

As a compromise to Reviewer 3's position on this matter, we have removed the Pb/Ce vs Sm/Lu panel in Supplementary Fig. 7 and added alternative evidence for flux melting, as suggested by Reviewer 2.

Changes made in response to comment:

Main Text:

Lines 205-207: The assertion of fluxed melting is further supported by the trends observed on plots of K_2O vs K_2O/Na_2O and Yb vs Zr, which suggest a fluid-, rather than melt-dominated "slab" component in the source of most Whundo lavas (Supplementary Fig. 7a-b).

Supplementary Figures:

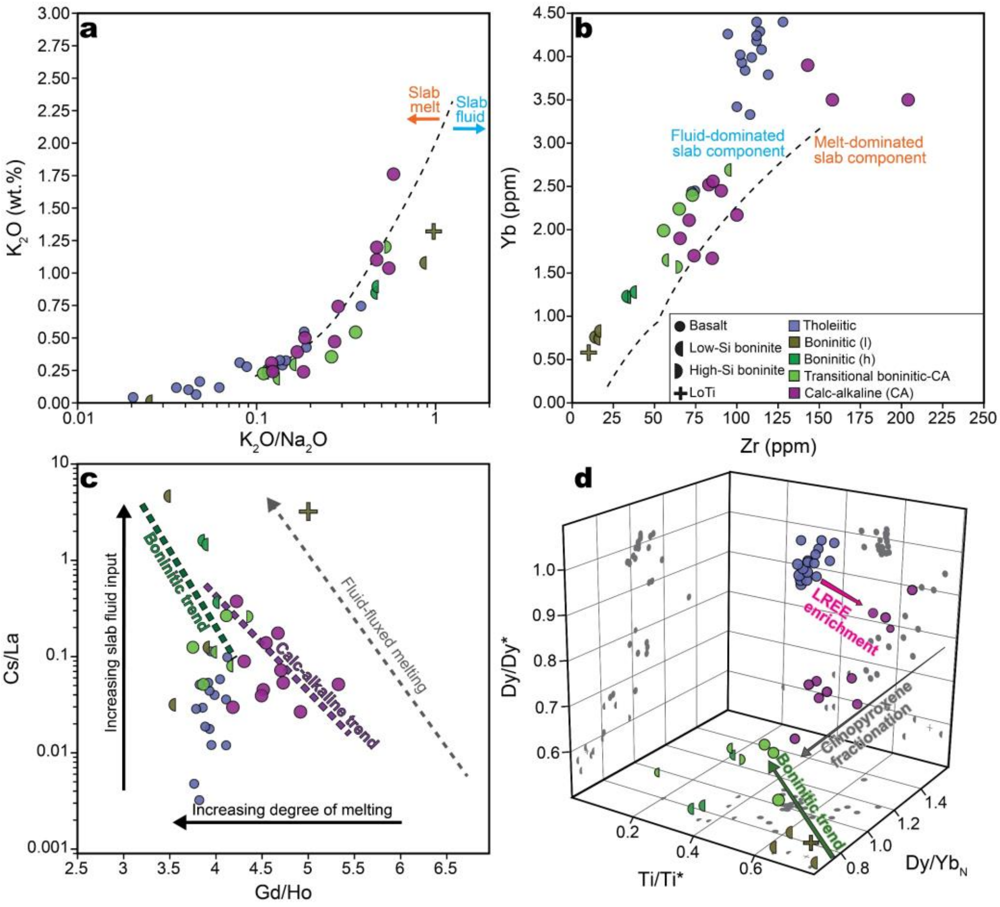

NEW: Supplementary Figure 7 (merged Supplementary Figures 7 and 8 from original manuscript with Figure R1 from previous round of reviews): added plots of K_2O vs K_2O/Na_2O (a) and Yb vs Zr (b) after Schmidt and Jagoutz (2017), differentiating between melt- vs fluid-dominated slab inputs.

<u>Comment:</u> Fig 5 and Line 234 – "Negative covariations of Pb/Ce vs. Sm/Lu and Ba/Th and Cs/La vs. Gd/Ho indicate that the calc-alkaline magmas were produced by fluxed melting" – same lines as the previous comment. The U-shaped REE is a very strong petrological indicator, together with some other HFSE. The manuscript would be much better if the arguments were built around those elements instead of LILE. There's no need to use Pb/Ce or Ba/Th ratios to claim flux melting, given that calc-alkaline rocks, by definition, have a late Al enrichment caused by the depolymerization of a fluxed magma.

<u>Response:</u> This is a very good point. We thank the reviewer for the suggestion. Our use of the Cs/La and Ba/Th ratios builds upon the evidence from Smithies *et al.* (2005). There may be no need to do so, but it is certainly one avenue of evidence, which we stress, is a relatively minor aspect in the grand scheme of our findings. As a compromise to the reviewer's position on the matter, we have moderated discussion of these proxies in the revised version of the manuscript, removed the Pb/Ce vs Sm/Lu panel in Supplementary Fig. 7 and as suggested by Reviewer 2, added alternative evidence for flux melting.

<u>Changes made in response to comment:</u>

Main Text:

Lines 205-207: The assertion of fluxed melting is further supported by the trends observed on plots of K_2O vs K_2O/Na_2O and Yb vs Zr, which suggest a fluid-, rather than melt-dominated "slab" component in the source of most Whundo lavas (Supplementary Fig. 7a-b).

Supplementary Figures:

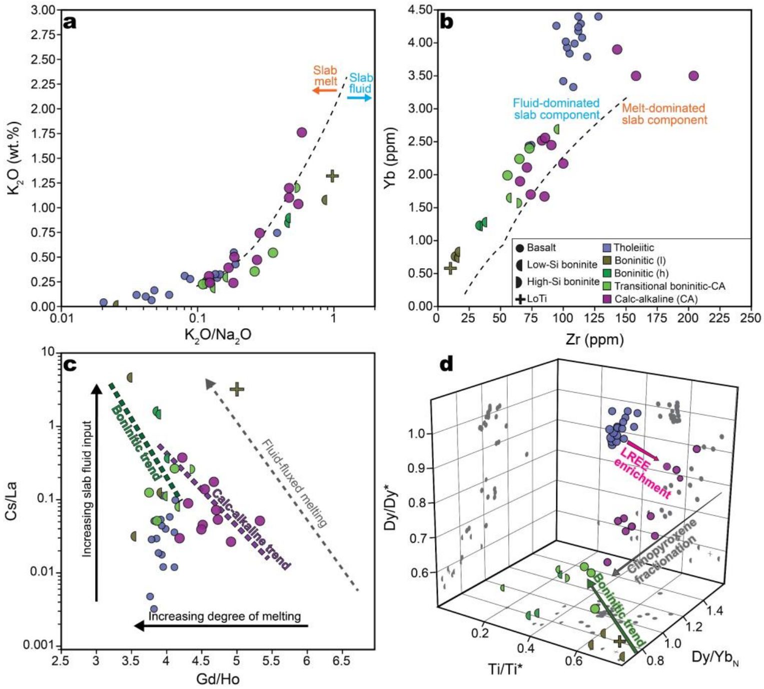

NEW: **Supplementary Figure 7 (merged Supplementary Figures 7 and 8 from original manuscript with Figure R1 from previous round of reviews):** added plots of K_2O vs K_2O/Na_2O (a) and Yb vs Zr (b) after Schmidt and Jagoutz (2017), differentiating between melt- vs fluid-dominated slab inputs.

Comment: **Line 263 – "First, we corrected representative samples from each group for fractional crystallization back to equilibrium with mantle olivine, which enabled us to calculate the trace element compositions of the parental melts."** – Include the composition(s) of the olivine back-calculated to (Fo86 and 90 I suppose).

Response: Change made.

Changes made in response to comment:
Main Text, lines 233-235 (changes made in bold): "First, we corrected representative samples from each group for fractional crystallization back to equilibrium with mantle olivine **(Fo_{86-90}),** which enabled us to calculate the trace element compositions of the parental melts."

Comment: Line 268 – "HREE budgets are fluid-immobile and unaffected by subduction enrichment, so we matched the calculated HREE abundances of the modified sources with those **produced by anhydrous partial melting of a primitive mantle source."** – This sentence is a bit confusing; would you mind rephrasing? I understood that you match the HREE of the modelled mantles, is that right?

Response: Yes, that is correct; we match the HREE of the modelled mantle sources. We have now clarified this in the main manuscript.

Changes made in response to comment:
Main Text, lines 238-242 (changes made in bold): "HREE budgets are fluid-immobile and unaffected by subduction enrichment, so we matched the calculated HREE abundances of the modified **mantle** sources **with the melting residues of a primitive mantle source along a mantle potential temperature adiabat, in this case 1,400°C.**"

Comment: **Line 271 – "This allowed us to estimate the amount of mantle wedge depletion before dripduction and characterize the contribution of the subduction component to mantle budgets of incompatible trace elements."** – Which incompatible elements? The calculation for the REE and some HFSE should be okay. I'm curious about the U and Th given the radiogenic nature, would the decay of 238U and 235U influence the concentration given the 3.1 Ga age of the samples and the sub ppm concentration measured?

Response: Whole rock U and Th contents in Archean samples aren't corrected for radioactive decay; Archean studies take these contents at face value. U and Th contents in zircons are often corrected, yes, but we can't think of a single example where this has been done for whole rock samples. As we do not know the isotope ratios, there's no way to accurately correct for radioactive decay, nor is there any precedent to merit this convention. To answer the question, it would likely increase the Th and U concentrations, but not by much. This would be dwarfed by both natural variations and analytical precision.

No changes made in response to comment.

Comment: **Line 276; line 965 ¶ on how the mass balance was done;** – In simple terms the calculation is the [x]initial mantle – [x]modified mantle = [x]change; where the change is presented as % and [x] is the concentration of each element. I suggest having an equation on the explanation to be clearer given thar some equations are already used on the supplements.

Response: We thank the reviewer for the suggestion; we have updated the *Methods* section of the Main Text accordingly to address their recommendation.

Changes made in response to comment:

Main Text (Methods), lines 1063-1070 (changes made in bold): "The relative contributions of the dripduction components were calculated by taking the difference in incompatible trace element abundances between the modified and pre-dripduction mantle wedges as a percentage relative to the modified wedge abundances **using the following equation:**

$$\Delta C\ (\%) = \left(\frac{C_{mw} - C_s}{C_{mw}}\right) \times 100 \qquad \text{(Eq. 11)}$$

Where ΔC is the contribution of the dripduction component to the budget of a given incompatible trace element, C_{mw} is the concentration of that element in the modified mantle wedge, and C_s is the concentration in the unmodified mantle wedge."

Comment: **Line 388- "… surrounding mantle (Fig. 6ci), providing up to 98% of the element budget of the…"** I just realized that U is up to 98%. See my comment about line 271 above!

Response: We are not entirely sure what the reviewer wants us to do here and feel that this point is not particularly germane. ^{232}Th hasn't even gone through one half-life since the Big Bang. Furthermore, as discussed above, there's no precedent to

warrant U and Th corrections in our samples. This would be dwarfed by both natural variations and analytical precision.

In the revised version of the manuscript, the revised methodology has lowered the dripduction contribution to the U budget of the boninitic mantle sources from 98% to 92%. This is well within the range of what has been inferred for Phanerozoic boninites (e.g., König *et al.*, 2010) (as was also the case at 98%), so it is unlikely that it has been affected by radioactive decay.

No changes made in response to comment.

Comment: **Line 393 – "Calc-alkaline basalts form when the arc melting regime comprises relatively undepleted lherzolite (Fig. 6ci). Later, when residual harzburgites enter the dripduction arc melting regime, fluxed melting produces boninites exhibiting the "ghost garnet" memory of the source's prior depletion history (Fig. 6cii)."** – So, the boninites would form after the calc-alkaline. That is the opposite of what normally occurs in modern settings, where boninites are the very first to form in most cases. The model needs the depletion of a lherzolite, and refertilize the depleted mantle, now a harzburgite, to produce boninites with some of the boninites mixing with the calc-alkaline.

Response: The boninites do not necessarily have to form after the calc-alkaline basalts, as both are diachronous (while boninites do appear first in the stratigraphy, this might be due to the base of Whundo being an intrusive contact). We have removed the word "later" from the second sentence to avoid giving any sense of one only occurring after the other.

While this is the case in forearc subduction-initiation settings, it is a common misconception that boninites are always the first to form in subduction zones, likely because many studies on boninites have relied on samples from forearcs themselves, such as the Izu-Bonin Marianas, or from ophiolites such as Oman and Troodos, which record subduction initiation in a forearc setting. However, there are other parts of subduction systems in space and time where boninites occur with much more random stratigraphy. For example, the Yavuna Group of Fiji contains interbedded boninitic, tholeiitic and calc-alkaline lavas erupted contemporaneously during the transition from the protoarc to the first arc stages of subduction initiation, away from the plate boundary (e.g., Pearce and Reagan, 2019; Todd *et al.*, 2012). Similarly, Pearce and Reagan (2019) suggested that the Whundo boninites best

resemble those from modern arc-basin settings, where there is no diagnostic stratigraphic progression. Thus, as we mentioned in our response to Reviewer 1, this observation demonstrates that the Whundo Group did not erupt in a forearc-analogous setting. We have added a paragraph that touches on this point.

Our model for boninite petrogenesis, involving transport of residues via mantle flow towards the arc melting regime, invokes processes similar to those inferred to generate boninites in modern arc-basin settings, where a similar lack of systematic stratigraphic variation is observed, and calc-alkaline BADR magmas can erupt both before and after (e.g., Cooper *et al.*, 2010; Pearce and Reagan, 2019). However, we stress to the reviewer that the depletion event that produces the ultra-refractory harzburgite component of the boninite source occurs long before dripduction starts, a point emphasized in both the text and Figure 6.

Changes made in response to comment:

Main Text:

Lines 139-141: added information about the boninites' closest phanerozoic analogues, based on previous work by Pearce and Reagan (2019): "Previous work based on their major element and Nb/Yb-Th/Yb systematics have shown that low-Si arc-basin boninites may be the Whundo boninites' closest Phanerozoic analogues[25]."

Line 363: removed "Later"

Lines 411-424: addressed the stratigraphic variability and lack of resemblance to forearcs and ophiolites that record subduction initiation, and how this can be reconciled with other parts of a convergent margin system: "As evidenced by the varied, albeit weakly cyclic stratigraphy of the Whundo Group (Fig. 1b), it is important to recognize that these primitive lavas, particularly the calc-alkaline basalts, boninites and their hybrids, were erupted diachronously. This contrasts heavily with the typical uniformity and stratigraphic progression from tholeiite to boninite and finally to calc-alkaline BADR lavas that is characteristic of subduction initiation recorded in Phanerozoic forearcs and ophiolites[24,25]. Thus, given the available evidence, including the similarities between the Whundo boninites and their modern arc-basin counterparts[25], Whundo is unlikely to be an Archean analogue of subduction initiation in a forearc setting. Understanding this stratigraphic variability and cyclicity is beyond the scope of this study, but these observations remain reconcilable with Whundo as an analogue of another portion of a convergent margin setting, such as an arc-front or arc-basin setting, where different lavas erupt diachronously[25,28], even during subduction initiation[63]."

A few points regarding the model:

Comment: – the timing difference comparing to modern subduction is interesting, I'd highlight that and expand on it.

Response: That is beyond the scope of this study. It is something that will be addressed in Vandenburg *et al.* (*in prep.*).

No changes made in response to comment.

Comment: – I'll point out (again) that contamination needs to be addressed. Mixing magmas, where both are liquids on the source, and contamination with a liquid and a solid on the crust, can be difficult to tell a part purely by WR major and trace element geochemistry. If the calc-alkaline composition can be used to mix and obtain some of the boninites, for me that also suggest that some of your boninites are contaminated. Especially given the proposed model where the green intrudes the gray on panel 6 c(ii). Bottom line, the mixing between the calc-alkaline and boninites could also be seem as contamination, and some of your calc-alkaline are show strong signs of contamination by the Whundo felsic.

Response: We appreciate the reviewer's concern about crustal contamination and agree that this can be difficult to elucidate with whole-rock geochemistry. That is why we take a holistic approach to ruling out contamination from a pre-existing ancient felsic basement, incorporating multiple lines of geochemical and geological evidence rather than relying entirely on whole rock geochemistry. We refer the reviewer to lines 172-185 in the previous version of the manuscript:

"The absence of >3.3 Ga Nd isotopic model ages[27] and >3.25 Ga inherited zircons in the Whundo felsic lavas and later intrusions in the area[24,35] are consistent with a thin, juvenile basement produced by rifting of the APB at 3.18 Ga. This is also observed in craton-scale variations in lithospheric thickness[36]. Furthermore, the similarity between the Th/Yb-Nb/Yb systematics of the primitive and felsic[24] lavas (Fig. 2b) suggests that they were not contaminated by ancient crust and therefore, erupted off-plateau. While some degree of contamination by coeval felsic units cannot be ruled out, the lack of covariation of contamination-sensitive proxies with increasing differentiation indicates that contamination was not a major contributor to the incompatible element budgets of the primitive lavas (Supplementary Fig. 7). Therefore, the Th-LREE enrichments and Nb-Ta depletions observed in the boninitic and calc-alkaline lavas likely result from the mantle source's characteristics, specifically enrichment[23,24,27,35,37,38]."

We have now expanded upon this in the revised version of the manuscript, as noted above.

The boninites form an array parallel to- and located at the bottom of the modern arc array in Th/Yb-Nb/Yb space; such positioning is not produced by crustal contamination. Although one outlier sample, which is a hybrid boninite, seems like it has experienced some degree of crustal contamination at first glance, its major element and Th/Yb and Nb/Yb systematics are more consistent with modern high-Si boninites. It should be noted that even then, crustal contamination cannot produce high-Si boninitic compositions (refer to Figure 5 in Pearce and Reagan, 2019) and therefore likely is not the predominant factor. For modern arcs, this has also been demonstrated by Bénard et al. (2017, 2018).

We stress to the reviewer that Fig. 6 is a *cartoon* designed to illustrate the key processes discussed in this paper, and we do not believe it is reasonable to infer contamination on that basis. If we believed that contamination was a major process contributing to the generation of the boninites, tholeiites or calc-alkaline lavas, we would have labelled this process in the cartoon.

Changes made in response to comment:
Main Text, lines 179-189 (changes made in bold): "**Furthermore, the boninites and tholeiitic basalts exhibit array-parallel trends on a Th/Yb-Nb/Yb diagram, inconsistent with crustal contamination (Fig. 2b)**. **While the calc-alkaline basalts exhibit an oblique Th/Nb trend**, similarities between the Th/Yb-Nb/Yb systematics of the primitive and felsic**Error! Reference source not found.** lavas (Fig. 2b) suggest they were not contaminated by ancient crust and therefore, erupted off-plateau. While some degree of contamination by coeval felsic units cannot be ruled out, contamination-sensitive proxies lack covariations with increasing differentiation, indicating that contamination was not a major contributor to the incompatible element budgets of the primitive lavas (Supplementary Fig. 6). **Furthermore, some calc-alkaline basalts with the highest MgO also have the highest Th/Nb, thus reconcilable with variable mantle wedge enrichment (Fig. 2b).**"

Comment: – The mixing calculations used average compositions. I'd suggest also using the extreme compositions (highest and lowest MgO, SiO2 or whichever differentiation parameter you choose). That might be more realistic instead of an average in terms of significance. If you decide to use the average, it's important to tell the number of samples used to calculate it and the 2 standard deviation.

Response: We appreciate the reviewer's suggestions here, however, adding extreme compositions to the diagram would make the panel too busy in our opinion. Furthermore, because spidergrams are log-scaled, it is not straightforward to add 2σ values, as the lower bound will technically extend into negative space. We note that other modelling papers published in similar journals (e.g., Smithies *et al.*, 2021) do not plot 2σ for their averages in their spidergram modelling figures.

We have added sample counts to the annotations. Owing to the smaller sample sizes of the l-boninites and hybrid basalts, we have rerun the calculations using the geometric mean rather than arithmetic mean for all three starting lithologies in panel d, as this is less susceptible to outliers in small sample sizes.

Changes made in response to comment:

Main Text Figures:

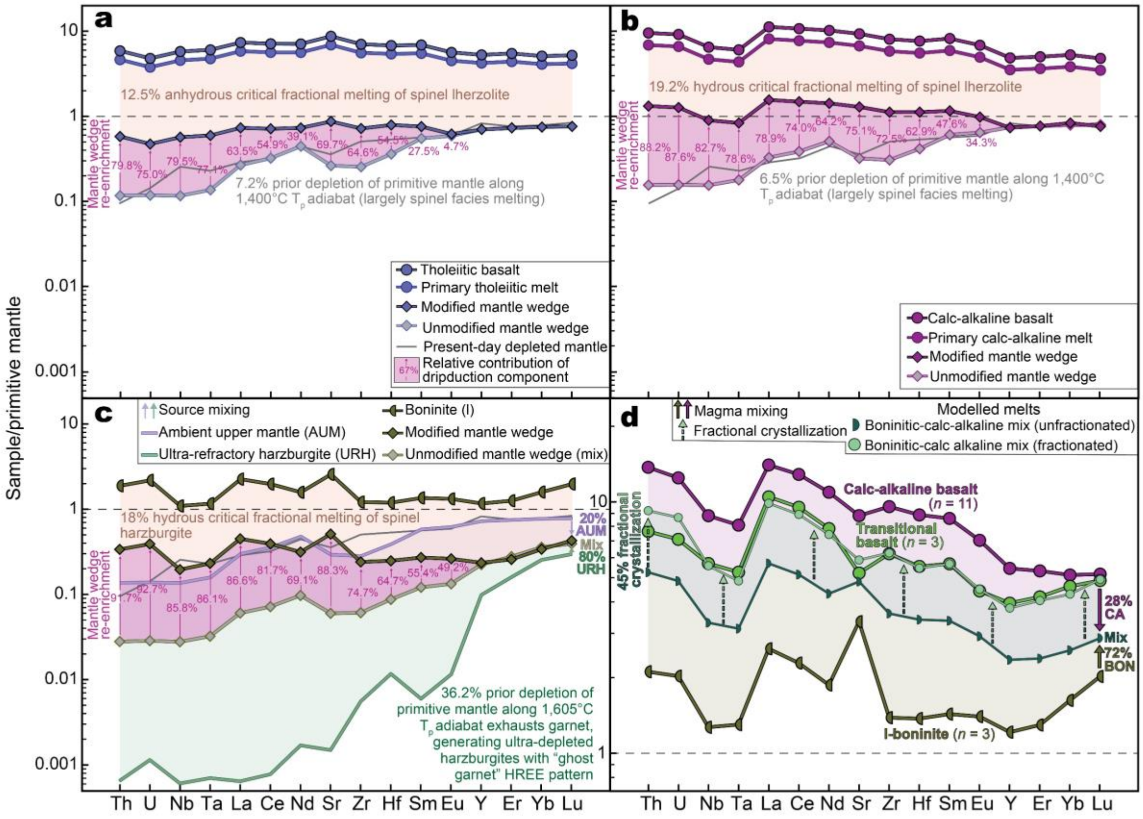

UPDATED: **Figure 4:** reran calculations for panel d using geometric means and added sample counts to the panel.

Responses to feedback from Reviewer #4:

The manuscript addresses a highly interesting topic, combining geochemical data with thermodynamic modeling, with particular emphasis on the role of water in the formation of boninite and calc-alkaline rocks during the Archean, prior to the onset of plate tectonics. I was invited as a co-reviewer specifically to evaluate the methodological aspects of the study; therefore, my comments will focus on part of the methodology (modeling) and will not address interpretations or discussions presented beyond that section.

1) Main Text:

Comment: Most of my comments in the main text relate to the figures. Overall, the figures would benefit from improved readability and visual optimization. In several cases, font sizes are too small or lack sufficient contrast to be easily read at a standard 100% page scale. Adjusting font sizes, improving color contrast, and adding clearer backgrounds where necessary would significantly enhance figure clarity and accessibility.

Response: We thank the reviewer for the suggestion; the changes have been made.

Changes made in response to comment:
See below.

Comment: Figure 1: The legends in the map (a) and stratigraphic column (b) are too small and difficult to read without zooming in beyond 100% of the page. These should be adjusted to an appropriate font size to facilitate readability. In addition, the text within the field photographs is hard to read at the current figure size. If the size is maintained, I suggest adding a solid background to the text rather than relying solely on shadowing.

Response: Changes made.

Changes made in response to comment:
Main Text Figures:

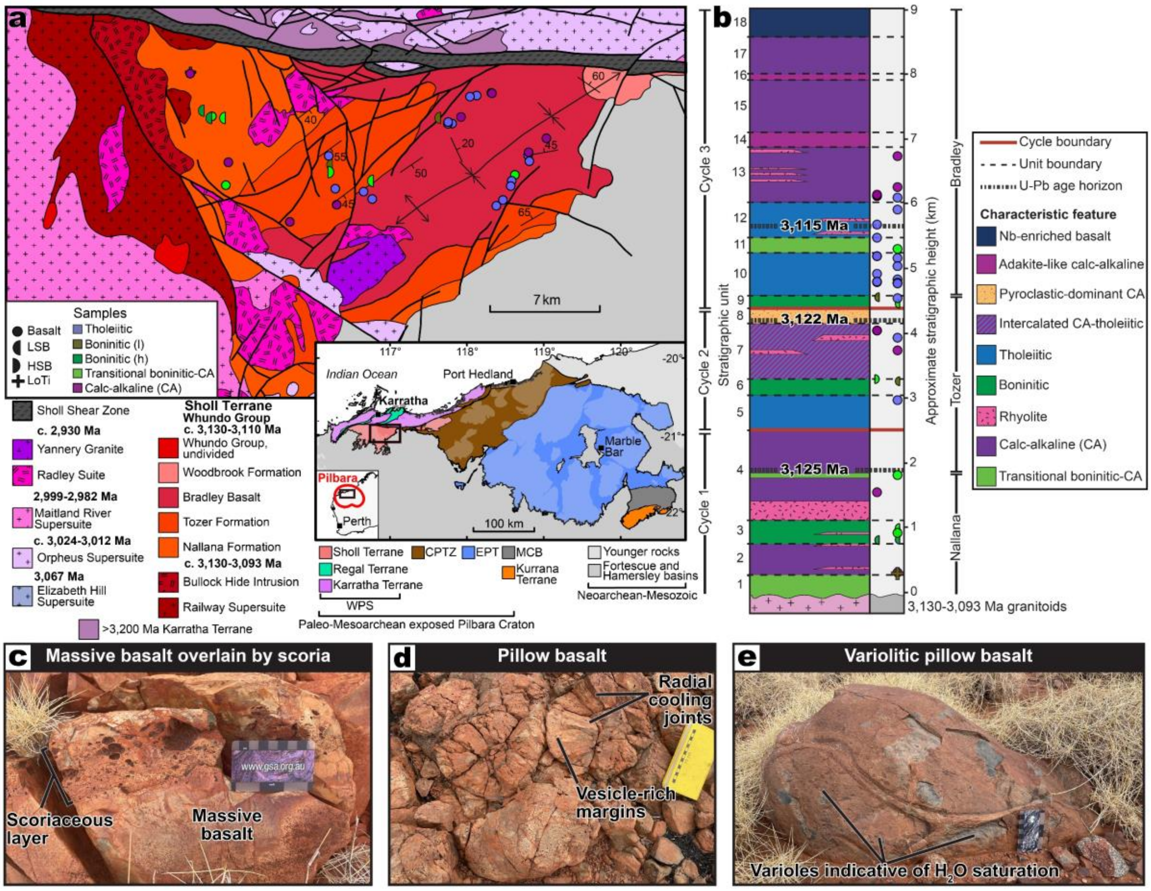

UPDATED: Figure 1: increased font size in legends and field photographs.

Comment: Figure 3: All graphs use a range from 0.1 to 100, resulting in approximately two-thirds of the plotted area being blank. While I understand the intention to maintain a consistent scale across panels, I suggest reducing the lower limit of the range (e.g., starting at ~0.8), which would eliminate a large unused portion of the plots. This adjustment would make similarities and differences between the traces more apparent, as the current wide range tends to visually flatten the data.

Response: We thank the reviewer for this suggestion. This change has been made for both Figure 3 and Supplementary Figure 5.

Changes made in response to comment:

Main Text Figures:

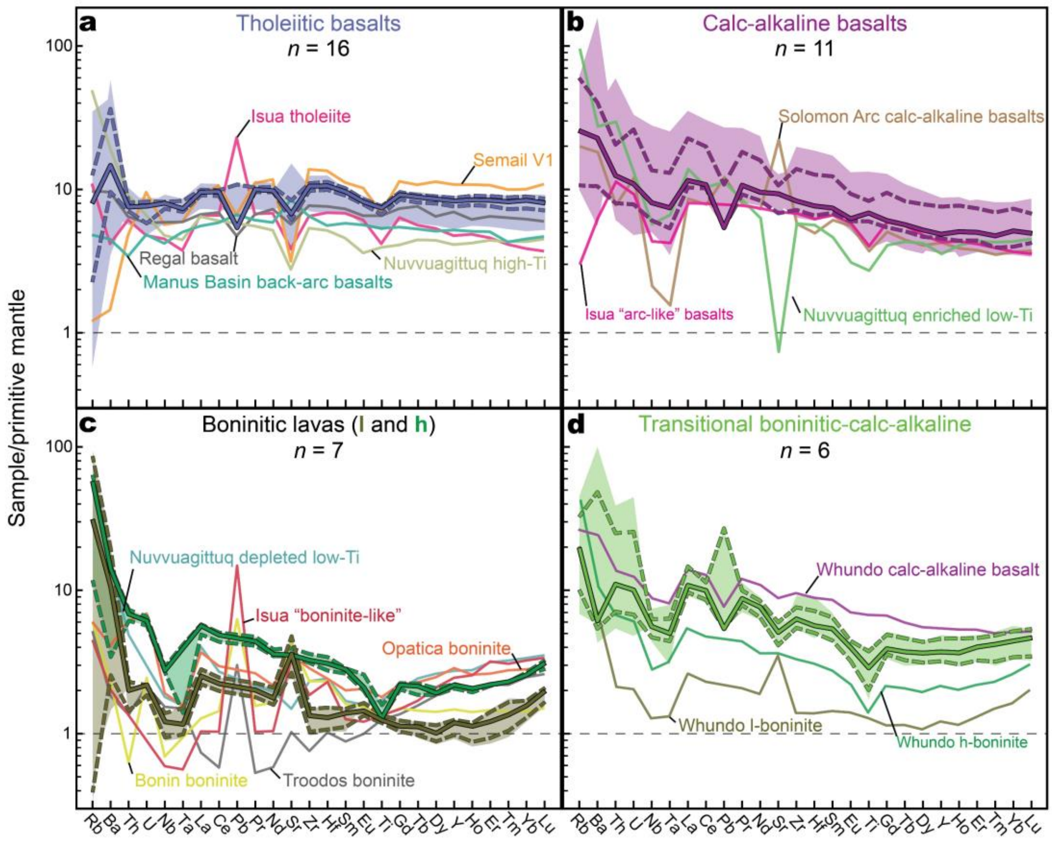

UPDATED: **Figure 3:** changed lower bound to 0.3 and decreased the aspect ratio of the figure to accentuate the similarities and differences between traces.

Supplementary Figures:

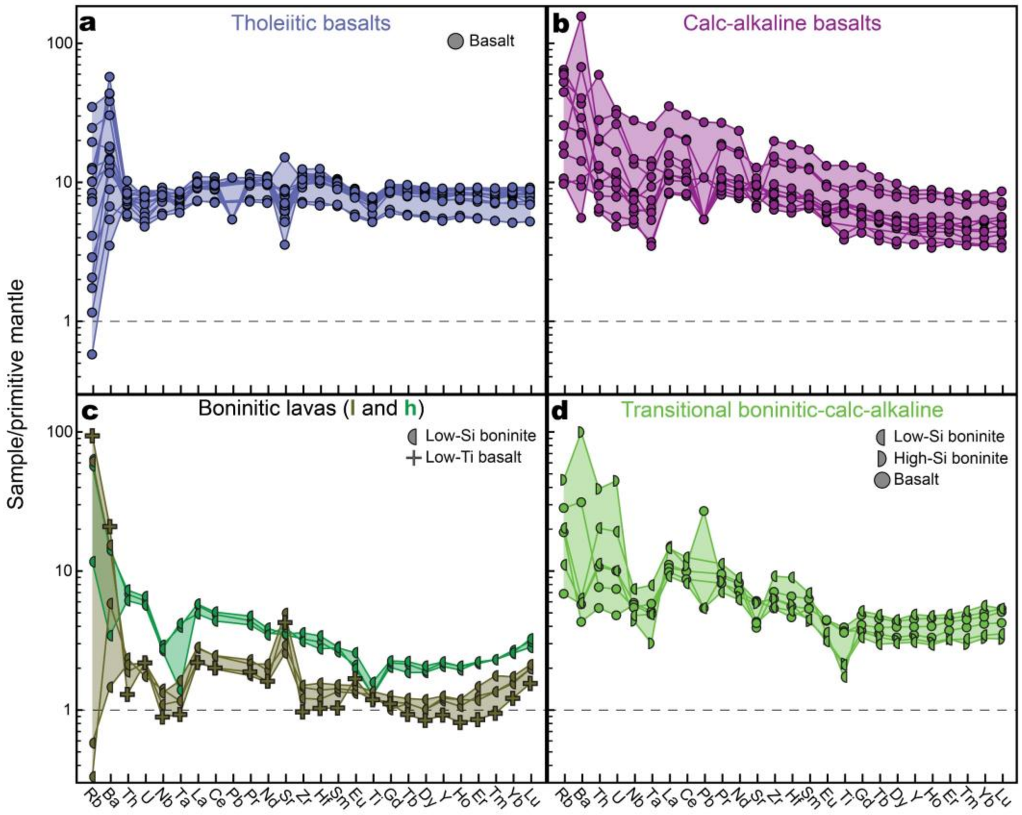

UPDATED: Supplementary Figure 5: changed lower bound to 0.3 and decreased the aspect ratio of the figure to accentuate the similarities and differences between traces.

Comment: Figure 4: The internal numbering within the figure panels is small and difficult to read and should be increased for better clarity.

Response: Good suggestion, we have increased the internal numbering within the figure panels where possible.

Changes made in response to comment:
Main Text Figures:

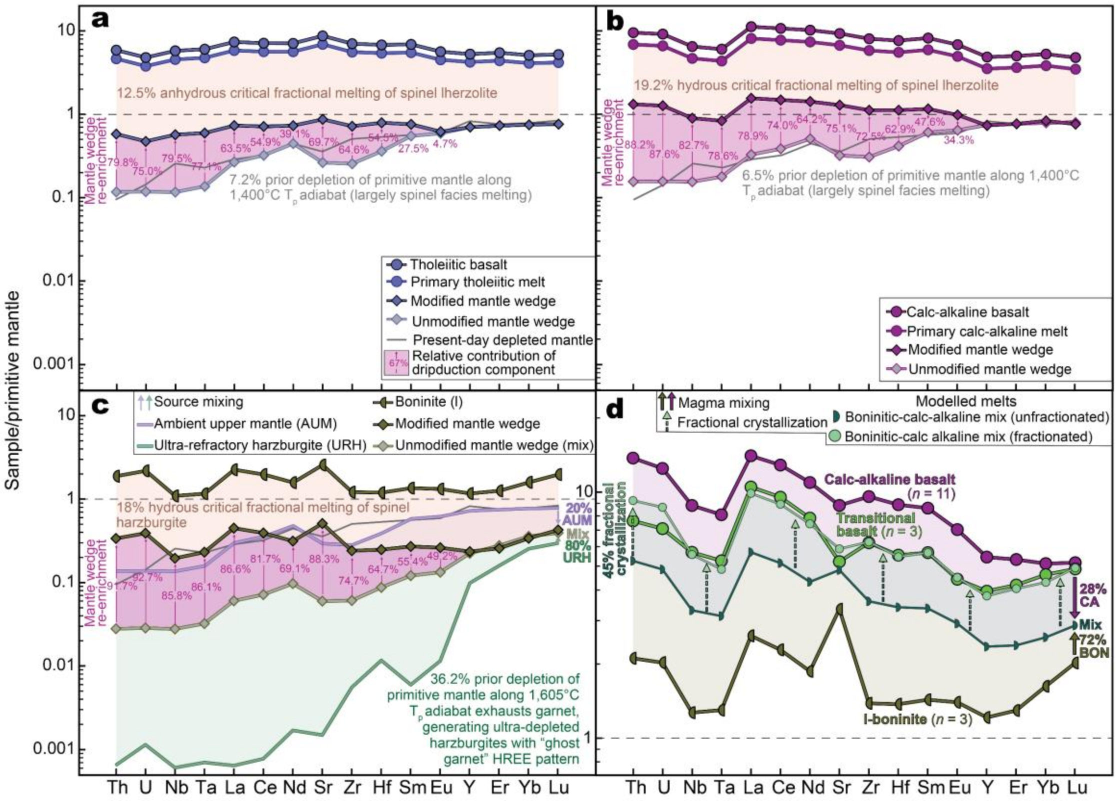

UPDATED: Figure 4: increased the size of the internal numbering in panels a-c, slightly decreased the aspect ratio to accentuate the similarities and differences between traces.

Comment: Figure 6: Some font sizes and color choices also hinder readability and would benefit from adjustment to improve contrast and legibility.

Response: We are unsure which fonts and colour choices the reviewer considers hindering readability. The lavas are coloured the way they are to ensure consistency within the figures, so we believe that those are best kept as-is. However, we have increased font sizes as much as possible within the figure.

Changes made in response to comment:

Main Text Figures:

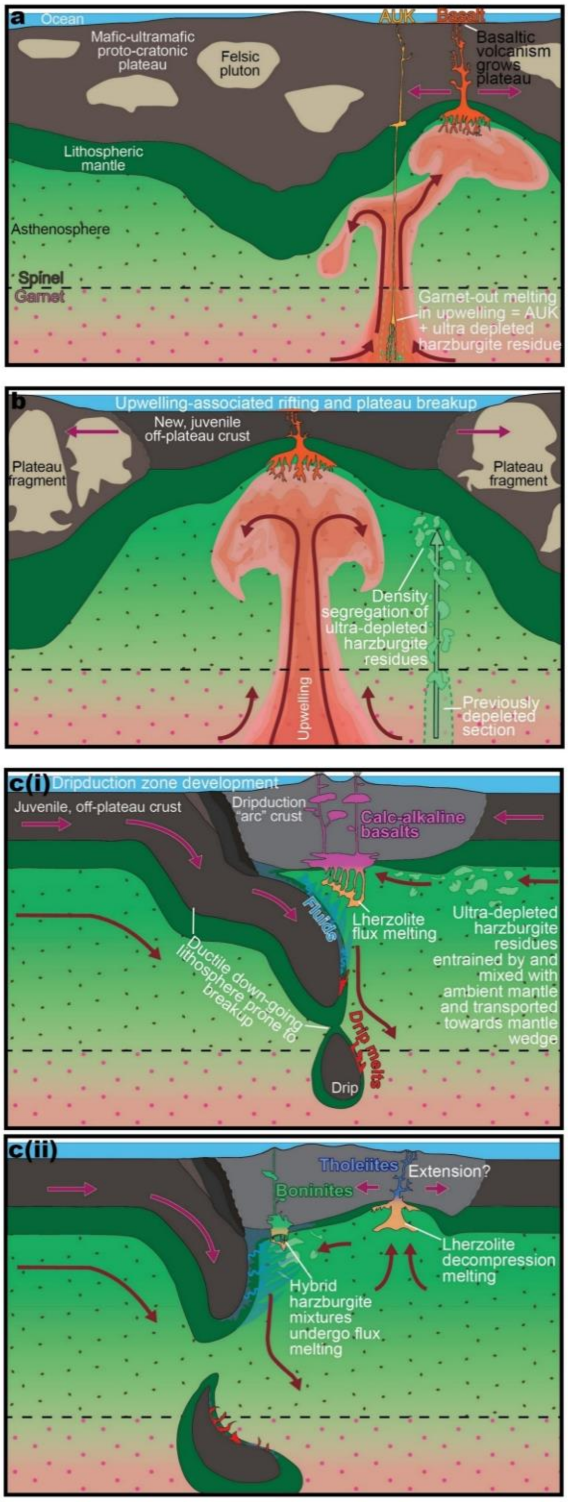

UPDATED: **Figure 6:** Increased font sizes in panels.

Comment: 2) Method and Supplementary Text 1
The manuscript presents a technically demanding and time-consuming modeling study. While such models are valuable tools for exploring rock-forming processes, they must be firmly grounded in geological evidence. At several points in the manuscript, the distinction between measured parameters and inferred or assumed parameters is difficult to follow, which complicates assessment of the model robustness.

Response: We appreciate the concern. We hope that our modifications to the manuscript and SI, and our responses to the reviewer, can help clarify and assuage their concerns.

Changes made in response to comment:
See below.

Comment: A critical issue concerns the characterization of the studied rocks. Much of the study is based on the premise that the rocks are pristine. Given that the samples are described as Archean boninites, it is essential to demonstrate convincingly that they preserve primary igneous features. Archean rocks are rarely pristine and are commonly affected by metamorphism and/or weathering. Therefore, the central premise of the manuscript (that the models represent primary igneous rock-forming processes) requires explicit and well-documented supporting evidence. Although the authors state that the samples are neither metamorphosed nor weathered, this claim is not supported by petrographic evidence in the current manuscript. The study relies heavily on thermodynamic and geochemical modeling, yet provides no thin-section documentation demonstrating the preservation of primary igneous mineral assemblages and textures. This represents a significant shortcoming.

Response: We once again appreciate the reviewer's concern, but we strongly believe that we have provided all requisite evidence in the Main Manuscript, SI, and our responses to the reviewers' comments. As we mentioned previously, many of these aspects have already been investigated in Smithies *et al.* (2004, 2005) and touched upon to a lesser extent in Smithies *et al.* (2007, 2018). It is certainly possible for magmatic LILE to be preserved in Archean rocks; one such example would be the 3.33 Ga Commondale komatiites of the Kaapvaal Craton (Hoffmann and Wilson, 2017). Age forms no real criteria for gauging alteration. MORB that formed yesterday (in a geological sense) could be more altered than Archean basalt due to

hydrothermal activity. Alteration is not a guarantee of element mobility, as it can be isochemical, and hence, petrography is certainly not a silver bullet in addressing this. The gold standard in assessing chemical mobility in rocks, whether they be Archean arc-like basalts or modern MORB, is chemical parameters and proxies.

We note that we clearly stated in the original manuscript that the rocks in this study have undergone some degree of metamorphism/alteration. We refer the reviewer to the following passage in the previous version of the manuscript:

Main Text, lines 104-109: "*In particular, as* ***a result of the low metamorphic grade, testified by alteration screening****, the highly fluid-mobile elements in primitive lavas of the Whundo Group retain their primary concentrations (Supplementary Methods and Supplementary Fig. 2), making it an ideal suite to test the hypothesis of Archean dripduction using geochemical modelling.*"

In the supplementary information, we simply ruled out significant overprint on LILEs, so we are unsure where the reviewer got the notion that we state that there was no metamorphism/alteration. However, we have added some hedging to the main manuscript to emphasize this point.

Changes made in response to comment:

Main Text, lines 104-109 (changes made in bold): "In particular, as a result of the **relatively** low metamorphic grade, testified by alteration screening, the highly fluid-mobile elements in primitive lavas of the Whundo Group retain their primary concentrations (Supplementary Methods, Supplementary Fig. 2, **and ref. 26**), making it an ideal suite to test the hypothesis of Archean dripduction using geochemical modelling.

Comment: Petrography is a fundamental starting point in geological studies, particularly when dealing with Archean rocks, which are rarely pristine and are commonly affected by metamorphism, deformation, and/or alteration. Petrographic observations are necessary to assess the presence or absence of metamorphic recrystallization, deformation fabrics, and secondary alteration phases that could substantially influence whole-rock compositions. Without such information, it is difficult to evaluate whether the compositions used in the models genuinely represent primary magmatic conditions.

The authors rely primarily on geochemical diagrams to argue for the pristine nature of the samples. While informative, geochemical evidence alone is insufficient without complementary petrographic documentation. The inclusion of representative thin-section images (in plane- and cross-polarized light) showing preserved igneous textures would substantially strengthen the manuscript and increase confidence in

the modeling results.
In the absence of clear petrographic evidence for preserved igneous textures, the central premise of the study remains insufficiently supported. Addressing this issue is essential and, in my view, necessary.

Response: The gold standard in assessing chemical mobility in rocks, whether they be Archean arc-like basalts or modern MORB, is chemical parameters and proxies. We demonstrate there is little evidence that the LILE exhibited substantial mobility based on chemical parameters and proxies; this has also been verified by Reviewer 2.

Optical microscopy is not a reliable method of evaluating elemental mobility. It can provide insights into alteration assemblages, recrystallization and deformation fabrics present in a rock, but it is a qualitative rather than quantitative analytical method and can be misleading. Furthermore, metamorphism can occur without redistribution of fluid-mobile elements on the outcrop scale, depending on the metamorphic reactions, thereby accounting for the replacement of some primary minerals while retaining the magmatic LILE concentrations on the whole-rock sample scale.

Although we believe that a supplementary figure with photomicrographs is unnecessary, we have provided these in Figure R5 to assuage Reviewers 3 and 4's concerns. Note that because we only have a very limited number of thin sections, some of the photomicrographs belong to samples that have not been used in this study (either because they are Nb-enriched basalts or because they are basaltic andesites).

As is evident in Figure R5, igneous textures in the samples have been preserved. Many of the basalts are comprised of acicular pyroxenes set in a fine-grained to glassy groundmass. The mineral assemblage and texture in 201633, classified as a high-Si boninite and comprised of acicular orthopyroxene set in a groundmass dominated by clinopyroxene microlites and glass, with no olivine, is characteristic of modern high-Si boninites (e.g., Pearce and Arculus, 2021). In line with Reviewer 2's comment, it is also evident that alteration has been largely restricted to minor to moderate degrees of chloritization and lesser sericitization of feldspars, which is remarkable for 3.1 Ga rocks.

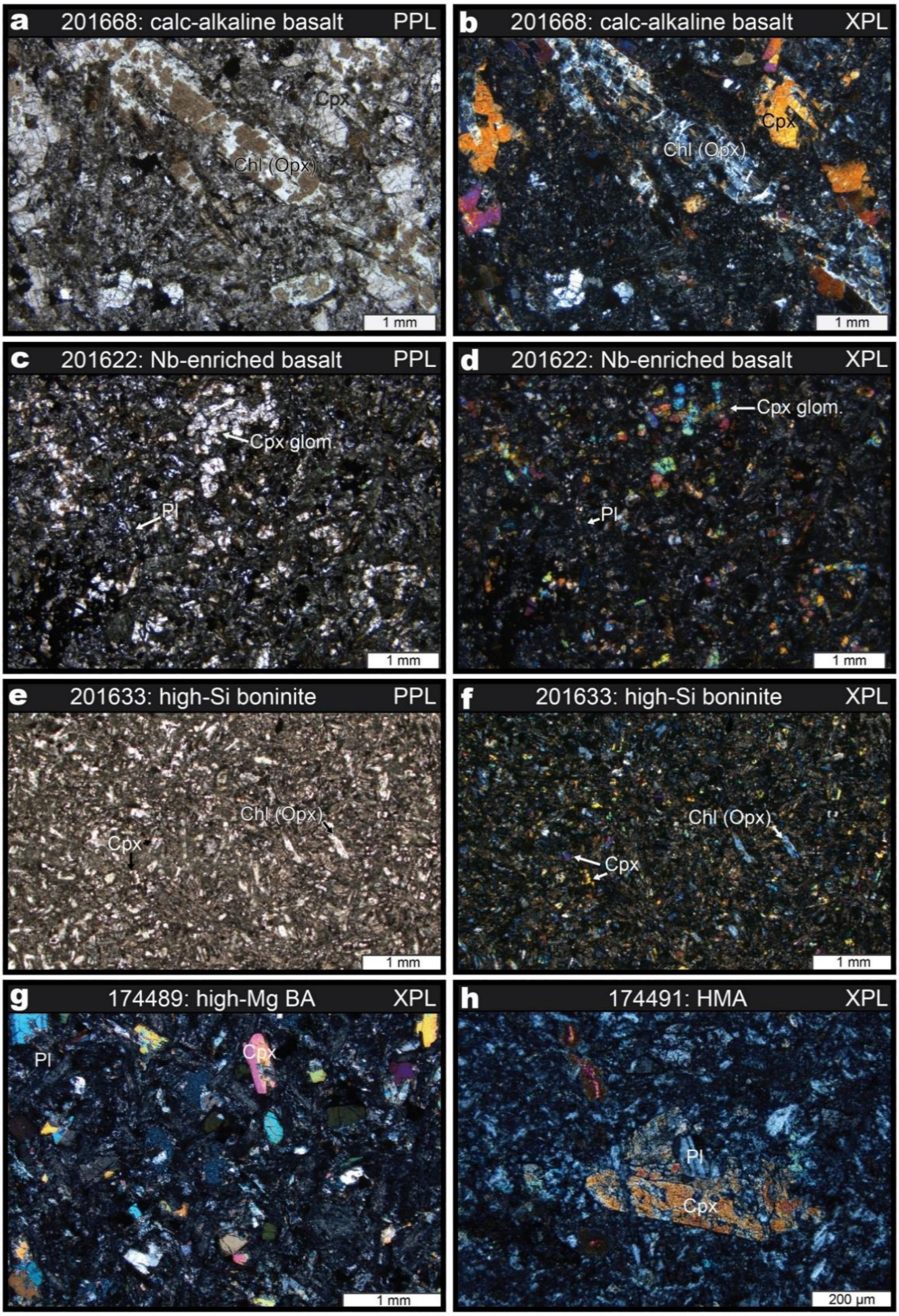

Figure R5. **Petrographic evidence for preserved igneous textures in Whundo Group lavas.** Plane-polarized (PPL; a) and cross-polarized (XPL; b) light photomicrographs of calc-alkaline basalt sample 201668 displaying medium-grained acicular orthopyroxene (now altered to chlorite) and subhedral clinopyroxene phenocrysts. PPL (c) and XPL (d) photomicrographs of Nb-enriched basalt sample 201622, with an intergranular texture

comprised of fine-grained clinopyroxene glomerocrysts, sericite-dusted plagioclase, oxides, and cryptocrystalline groundmass. PPL (e) and XPL (f) photomicrographs of high-Si boninite sample 201633, consisting of fine-grained acicular orthopyroxene set in a groundmass of microcrystalline to very fine-grained clinopyroxene microlites and glass; this is very similar to modern high-Si boninites (e.g., Pearce and Arculus, 2021). XPL (g) photomicrograph of intergranular-textured high-Mg basaltic andesite (HMBA) sample 174489, consisting of fine-grained subhedral to euhedral clinopyroxene and fine-grained sericite-dusted plagioclase laths. XPL (h) photomicrograph of high-Mg andesite (HMA) sample 174491, displaying very fine-grained plagioclase microphenocrysts set in a groundmass of plagioclase microlites. Sample 201622 is from unpublished data in Vandenburg *et al.* (in prep.). Sample 174489 and 174491 are from Smithies *et al.* (2005).

No changes made in response to comment.

Alteration boxplot method.

Comment: One of the ways the authors attempt to "prove" that the rocks were not affected by metamorphism is by using the alteration box plot method published by Large et al. (2001). According to the original paper describing this method, it has several limitations: (a) it was developed for the study of alteration associated with VHMS systems, which is not the case here; (b) "the box plot is most suited to the study of hydrothermal alteration in felsic volcanics rather than mafic volcanics"; and (c) the authors clearly state that "the alteration box plot should not be used in isolation but is most powerful in the understanding of alteration when combined with petrographic and/or mineralogical studies."

Response: We thank the reader for their concern. We are aware that the original study of Large *et al.* (2001) was developed for VMS systems, but this is a consequence of the alteration types that the diagram quantifies; CCPI tracks carbonation, AI tracks sericitization, and both indices trace chloritization. We are using the diagram for exactly this. As supported by Reviewer 2's comments, there is a minor amount of chloritization as evidenced by this plot, but it is not extensive. The Whundo Group is also host to a significant VMS deposit, so regardless, this diagram applies to our study. Second, subsequent studies have shown that the alteration boxplot method is just as effective for mafic volcanic rocks, provided that the boundaries for relatively unaltered samples are determined by first plotting up definitively unaltered equivalents of the samples in question (e.g., Mathieu, 2018). It is important to note that we have not used the bounds for unaltered rocks as presented in Large *et al.* (2001), because, as the reviewer points out, these would not be relevant to the compositions studied here. As per the caption for

Supplementary Figure 2, the boundaries for unaltered modern arc lavas were "*determined using 2σ values for boninites [n = 191], basalts [n = 3,930], basaltic andesites [n = 5,322], and andesites [n = 4,081], compiled from the GeoRoc [4] and EarthChem Portal [5] databases*." Third, we are not using this diagram in isolation; it is a single panel in a larger Supplementary Figure 2. Therefore, we believe that our use of this diagram is valid and no changes have been made in response to this comment.

No changes made in response to comment.

Comment: Furthermore, this reinforces my comment above: the simplest way to demonstrate and convincingly show that the rocks are neither altered nor metamorphosed is to present photomicrographs of thin sections documenting pristine volcanic textures and primary mineralogy, as well as the absence of metamorphic phases. The field photographs provided are not sufficient to demonstrate this, as the rocks appear weathered in the images. Although the authors explain that weathered material was removed prior to geochemical analysis (a common and necessary step in geochemical studies, and one that is also reflected in the weathering plot) additional evidence is required to rule out metamorphic or hydrothermal overprinting.

Response: We would like to remind the reviewer that we did not state that there was *no* alteration, simply that the LILE mostly retain their magmatic concentrations. As discussed by Reviewer 2, there is indeed a minor amount of chloritization in our samples. However, it is perfectly reasonable to believe that this may be largely isochemical.

In addition, relating the disturbances of LILE systematics to photomicrographs is tricky business without knowledge of hydration. There is no evidence for extensive fluid flow (which would redistribute fluid-mobile elements along large distances). Furthermore, metamorphism can occur without redistribution of fluid-mobile elements on the outcrop scale (depending on the metamorphic reactions, and especially if the permeability is low, as are the lithologies that we are dealing with here), thereby accounting for the replacement of some of the primary minerals while at the same time retaining the magmatic LILE concentrations on the whole-rock sample scale.

We have already constructed a six-panel figure (Supplementary Figure 2) to support our claims regarding the lack of metamorphic and hydrothermal overprinting in the

form of a (Supplementary Figure 2). We also do not believe that it is necessary to create a second figure to further address metamorphic and hydrothermal overprinting, as this is a paper on the petrogenesis of primitive lavas, rather than a metamorphic study. The reviewer is allowed to disagree with our interpretation, and that's one of the great things about science, but we have provided more than enough evidence to support this interpretation. We also refer the reviewer to Figure R3.

Changes made in response to comment:

Supplementary Figures:

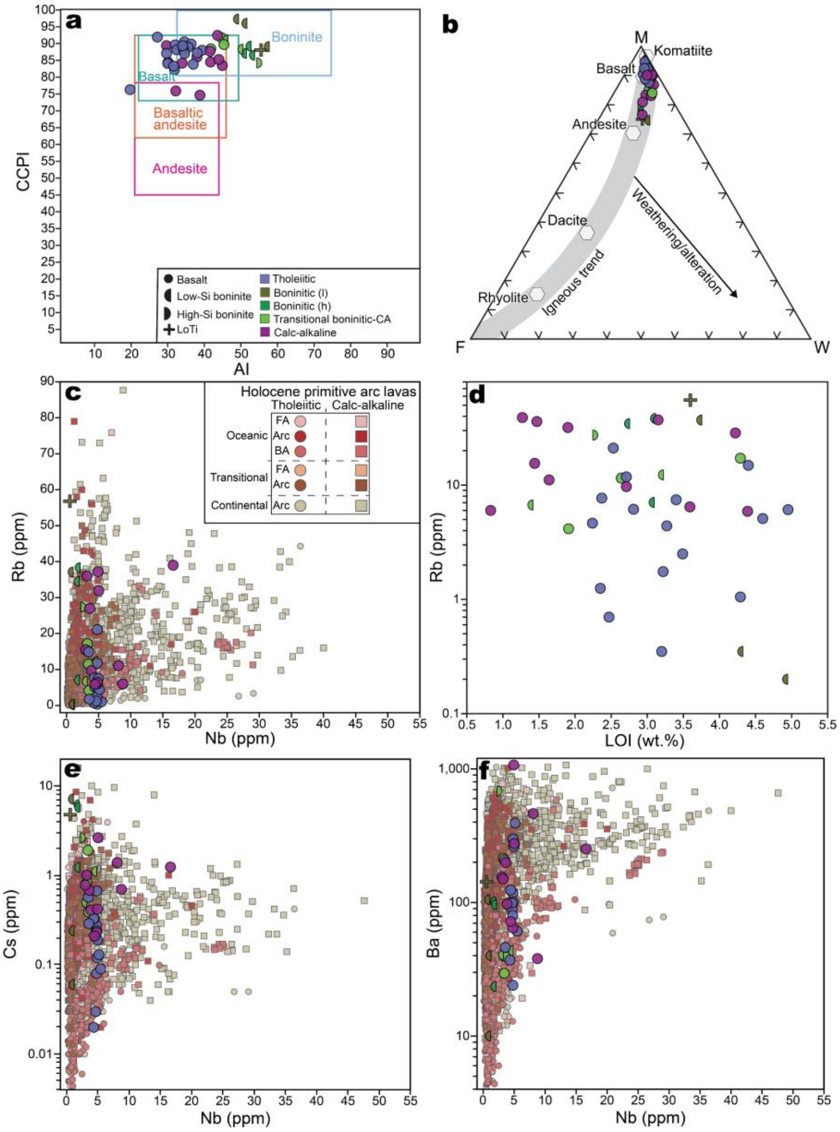

UPDATED: Supplementary Figure 2: added Cs vs Nb and Ba vs Nb plots, changed displayed Holocene arc lavas to unaltered basalts and boninites with MgO contents within the range of the samples presented in this study, rather than just primitive lavas.

Comment: Regarding the statement: “Moreover, Rb shows no correlation with loss-on-ignition (LOI), which is often invoked as a proxy for the degree of alteration, particularly in mafic–ultramafic rocks.” The absence of correlation alone in this graph is not sufficient to demonstrate a lack of alteration. Also, If LOI vs Rb standalone is indeed considered a “common” proxy for alteration, appropriate references supporting this assumption should be provided to support.

Response: We agree with the reviewer that the lack of correlation alone in Supplementary Fig. 2d is not sufficient to demonstrate a lack of alteration, but we would also like to emphasize to the reviewer that this is why the panel is part of a larger figure. None of the panels is standalone; they do not exist in isolation. Comparing abundances to LOI is a very common way of evaluating element mobility in mafic-ultramafic rocks (essentially undergraduate-level geochemistry/metamorphic petrology), but it’s not done in isolation. We do not feel that this comment is germane to the manuscript’s improvement. It would not be productive to provide a litany of studies on such a minor point of contention, so we hope that the addition of one reference is enough to appease the reviewer on this point.

Changes made in response to comment:
Supplementary Information, lines 38-40 (changes made in bold): “Moreover, Rb shows no correlation with loss-on-ignition (LOI), which is often invoked as a proxy for the degree of alteration, particularly in mafic-ultramafic rocks**[1]**.”

Thermodynamic Modelling

Comment: Theriak-Domino and pMELTS are both thermodynamic modeling tools, but they are not interchangeable and have important differences. Theriak-Domino is primarily optimized for subsolidus phase equilibria. Although recent studies have explored its application to melting processes, this use remains under development and should therefore be applied with caution (see comment below regarding errors in the Theriak-Domino database).

Response: We thank the reviewer for the concern, but this is factually inaccurate.

Theriak-Domino and MAGEMin (which we use in the revised version of the study) use the internally-consistent thermodynamic datasets of Holland and Powell (2011) and the subsequent a-x models developed from these. Otto *et al.* (2023) compared phase equilibrium models from pMELTS to the Holland and Powell (2011) derived datasets and found that the latter provide the best match to experimental results of

upper mantle melting. Furthermore, pMELTS lacks fidelity at >3 GPa and tends to overpredict garnet at unrealistically low pressures.

We remind the reviewer that just because these datasets remain under development, it does not mean they are imprecise or unsuitable for use; for example, these developments could be improvements that further increase precision or account for accessory/trace minerals. Because we are dealing with normal peridotitic mantle, the latter is even less of an issue (compared to granites, for example). Studies under suprasolidus conditions, including mantle melting, that use thermodynamic datasets built upon Holland and Powell (2011) have been published in a multitude of high-profile journals, including *Nature, Science Advances,* and *Nature Geoscience* (e.g., Walsh *et al.*, 2023; Ding *et al.*, 2025; Schmidt *et al.,* 2025; Lamont *et al.*, 2024; Soderman *et al.*, 2025).

Because we are no longer using Theriak-Domino, this comment has been addressed and resolved due to the detailed changes in the modelling. The modelling now solely employs MAGEMin (Riel *et al.*, 2022).

No changes made in response to comment.

Comment: An additional limitation is that Theriak-Domino does not calculate trace-element behavior. The modeling presented in this study focuses exclusively on major elements, yet trace elements are critical for evaluating melting, fractional crystallization, and magma mixing processes, particularly when comparing modeled results with natural rock compositions that have undergone these multiple evolutionary steps. While trace-element calculations cannot be performed directly within Theriak-Domino, they can be modeled using phase proportions derived from pMELTS. The manuscript would be significantly strengthened by including a comparison between observed and modeled trace-element compositions and by explicitly addressing expected trace-element behavior, especially where modeled and measured compositions diverge.

Response: We thank the reviewer for their concern, but we are unsure what they are getting at here.

Firstly, we have performed the trace element modelling of modified mantle wedge, as presented in Figure 4 and Supplementary Figure 8, using empirical methods and a flowchat can be found in Supplementary Figure 9. We have switched the mantle depletion modelling (first stage melting) over to a hybrid empirical-thermodynamic

model as discussed below, in the *Supplementary Methods*, and in Supplementary Figure 9.

While it cannot be done in the software itself, trace element modelling can be performed via empirical methods (i.e., partial melting equations) quite easily with the results of thermodynamic modelling software (e.g., MAGEMin, Theriak-Domino, Thermocalc, Perple_X). All that is needed is the phase abundances from the results, and mineral-melt partition coefficients for the elements and minerals of interest. In fact, this is quite a common method, particularly for crustal systems (e.g., Hernández-Montenegro *et al.*, 2021; Hoare *et al.*, 2023; Johnson *et al.*, 2017) and is equally applicable to the mantle, so we are unsure what the reviewer means when they say that Theriak-Domino is primarily for the subsolidus, given the published studies mentioned in the previous comment, and that the modern databases contain melt a-x models. We have switched our thermodynamic modelling over to MAGEMin because it is the next generation of thermodynamic phase equilibrium software, so any issues with Theriak-Domino are no longer a concern.

Rather than using static partition coefficients based on single experimental studies, we have increased the robustness of our first-stage mantle depletion modelling by using the outputs of the thermodynamic modelling to calculate variable mineral-melt partition coefficients using parameterized equations designed by previous studies incorporating experimental data; variable partition coefficients are only an option for clinopyroxene and garnet in pMELTS and are restricted to the lattice strain model, which is less accurate than the multi-parameter equations employed in our modelling. Furthermore, as discussed in the previous comment, the thermodynamic datasets employed in our modelling are much more aligned with the results of petrological experiments of mantle melting than pMELTS. So, in tandem, our trace element methodology is much more representative than pMELTS, especially considering that we model to much higher pressures (up to ~ 6 GPa).

Another important point to consider is the equations that we use here are the same as the ones in the integrated trace element framework in alphaMELTS (Smith and Asimow, 2005). The only difference is that ours are not built into the software. In essence, it all goes back to Denis Shaw and subsequent studies that build upon his work.

With respect to Figure 5, we are unsure what the reviewer means, as this figure is not concerned with trace element modelling.

Changes made in response to comment:

Modelling framework revised: the modelling now only uses MAGEMin, addressing Reviewer 4's concerns about consistency.

Comment: Section 1.7.2 introduces an additional modeling software, but the rationale for its use and its relationship to the other modeling approaches are not clearly explained. This lack of clarity makes it difficult to assess the internal consistency of the methodology.

Response: In response to the reviewer's comment, we have provided additional information that should clarify the relationship and rationale for the use of the Fractionated P-T software. Note that we already did this; provided Section 1.7.2 and Supplementary Figure 10 are considered together. However, we agree that it could be further clarified.

Changes made in response to comment:

Supplementary Information, lines 387-396 (changes made in bold): "**Therefore, their compositions can be input into the** Fractionated P-T software[54] to calculate melting P-T conditions for the primary boninitic magmas; **this data is then used to constrain the water content of the boninite mantle source** (Fig. 5b, Supplementary Figs. 8b, 10). **The pressure and temperature estimates produced by this program are independent of each other**. For each boninite sample, we calculated the P-T conditions of melting for a melt with 2, 3, and 5 wt.% H_2O (the range of H_2O in boninites from ref. 37), at molar $Fe^{3+}/\Sigma Fe$ of 0.11, 0.16, and 0.21 (0.21 being the $Fe^{3+}/\Sigma Fe$ of modern boninites; refs. 37, 55). **We used the olivine Fo content $Fo_{92.9}$, determined in MAGEMin using the major element composition of the modelled boninitic mantle source**."

Lines 399-400: added "For Figure 5b and Supplementary Figure 8b, we used a pressure of 1.70 GPa, which is in the middle of these estimates."

Comment: More generally, each modeling approach carries its own assumptions and limitations. The use of multiple software packages and calculation strategies within a single workflow can substantially increase uncertainty, particularly when outputs from one model are used as inputs or constraints for another. Without a clear justification for each modeling choice and an explicit discussion of uncertainty propagation across the workflow, it is difficult to any reader to evaluate and trust the robustness of the final interpretations.

Response: We understand the reviewer's concern on this point and have addressed this by consolidating all thermodynamic modelling into a single software package, MAGEMin (Riel *et al.*, 2022). In short, we have used MAGEMin to conduct our thermodynamic modelling because it currently provides the most robust and accurate results of any available method and is wrapped in a user-friendly UI. The dataset and a-x models employed here have been previously demonstrated to be vastly superior to pMELTS for modelling mantle melting and are robust up to a pressure of 7 GPa. As stated in Green *et al.* (2025), a σ uncertainty is ±1 kbar and ± 50°C for a boundary that introduces a phase that can reach significant proportions. We have added a section to the *Supplementary Methods* that discusses uncertainties associated with our approach. We also refer the reviewer to Holland *et al.* (2018), Riel *et al.* (2022), and Green *et al.* (2025) for a more general discussion.

Changes made in response to comment:
Supplementary Information, lines 494-548: Added section "Modelling uncertainties," which addresses Reviewer 4's concerns.

Comment: Given the large number of assumptions underlying the proposed model, methodological consistency is especially important. It is unclear for me why magma mixing and fractional crystallization were initially modeled using empirical approaches (e.g., the fractional melting equation of Shaw, 1970) rather than thermodynamic calculations using pMELTS (Ghiorso et al., 2002), which would provide a more modern and rigorous framework for evaluating phase relations and component behavior. Sections 1.2–1.6 could potentially be addressed using thermodynamic modeling, as was done in later sections, allowing element distributions to be evaluated within a phase-controlled framework using appropriate partition coefficients. This would result in a more internally consistent and robust interpretation.
Response: This comment has been addressed and resolved on account of the detailed changes in the modelling.

There's no straightforward way to perform inverse modelling of the mantle source using the pMELTS version of the alphaMELTS package. This is why we use empirical approaches for the mantle source modelling; it's straightforward to reproduce. Empirical equations such as the ones employed here have remained consistently in use for more than 50 years because they can be easily tailored to

incorporate the results of petrological experiments (e.g., non-modal melting reactions as employed here).

No changes made in response to comment.

Specific comments about each software:

Comment: a) Theriak Domino:
The manuscript states that the thermodynamic dataset dS733 was used. This database is not known, and if it was indeed employed, its source must be clearly specified. However, the reference cited in the text indicates the use of the well-known ds633 database. If this discrepancy is simply a typo, it should be corrected consistently throughout the manuscript.
If ds633 is the database actually used, the following issue must be addressed. An algebraic error in the formulation of the thermodynamic model for silicate melt in Holland et al. (2018) was identified, and Green et al. (2025) published a corrigendum for this dataset (DOI: 10.1093/petrology/egy048). Because this melt model forms a central component of the thermodynamic calculations employed in this study, the modeling should be re-run using the corrected database. The presence of a known error in the original formulation directly affects the reliability of the results presented here.

Response: As part of our revised modelling framework, we have re-run the calculations in MAGEMin using the corrected ds636/G25 of Green *et al.* (2025). With respect to mantle water content, this yielded very similar results to our previous methodology using the uncorrected dataset.

Changes made in response to comment:
Modelling framework revised using ds636/G25.

Comment: Moreover, the corrections and examples discussed in Green et al. (2025) are primarily applied to anhydrous peridotite systems. The applicability of this database to hydrous peridotite melting, which is fundamental to the present study, therefore remains uncertain and should be explicitly addressed. As noted by Green et al. (2025), “melting relations in peridotite systems are the subject of ongoing model development.” Consequently, the use of this database for hydrous peridotite modeling is itself non-trivial and requires careful justification and discussion of associated uncertainties.

Response: We appreciate the reviewer's concern here and have addressed this through our detailed changes in the modelling and by adding a section titled "modelling uncertainties" to the *Supplementary Methods*. As we mentioned before, just because these datasets remain under development, it does not mean they are unsuitable for use. In short, there remains significant debate surrounding the position of the water-saturated solidus in P-T space; experimental studies using the same bulk composition have yielded vastly different results at equal pressures (e.g., HZ1; Green *et al.*, 2010, 2014 vs Grove *et al.*, 2006 and Till *et al.,* 2011). To test the applicability of the x-eos to hydrous mantle melting, we calculated the H_2O-saturated solidus for fertile lherzolite HZ1 in MAGEMin, which we have plotted in Supplementary Figure 11. Unlike the solidus determined in pHMELTS, which substantially deviates from the results of experimental studies, the MAGEMin-determined H_2O-saturated solidus is reasonably similar to the results of most experimental studies at the pressures of interest to this study. A limitation of using MAGEMin and the Green *et al.* (2025) x-eos is that they don't account for water storage in nominally anhydrous minerals, which can be up to several hundred ppm (e.g., Green *et al.*, 2010). However, this means that our results can be considered minimum estimates of water content and remain valid.

Changes made in response to comment:

Supplementary Information, lines 494-548: Added section "Modelling uncertainties," which addresses Reviewer 4's concerns.

Supplementary Figures:

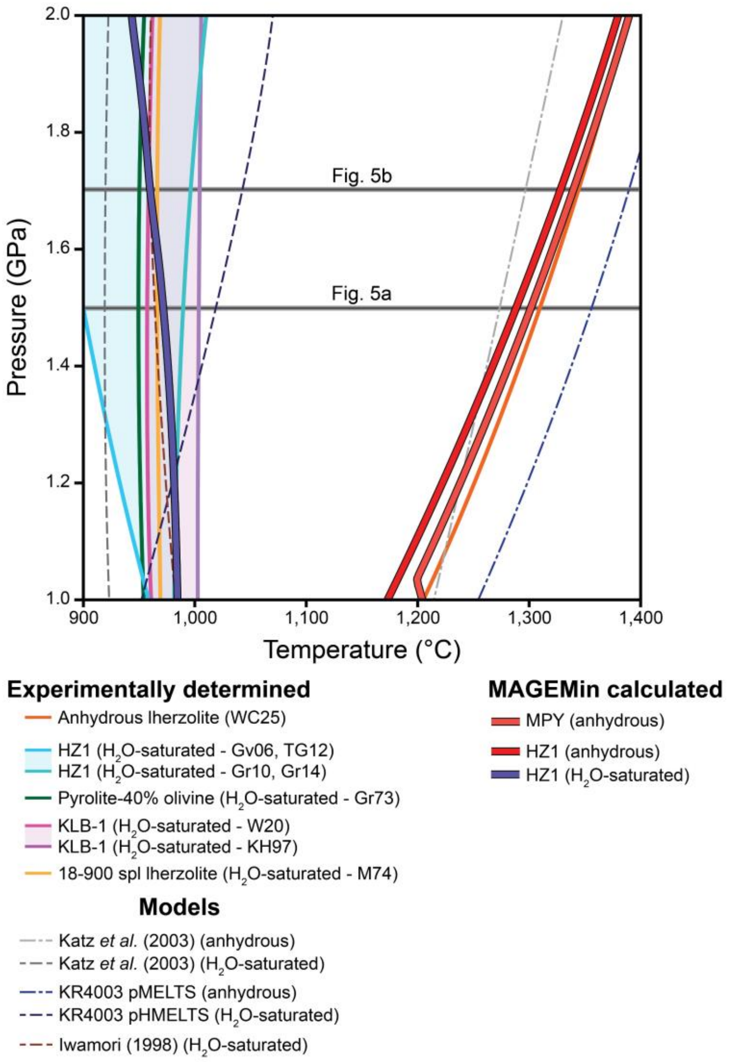

NEW: **Supplementary Figure 11.**

b) pMELTS

Comment: I note that several phases appear to have been suppressed in the modeling files. If this is the case, the rationale for phase suppression and its implications for the modeled melts must be explicitly discussed.

Response: This comment has been addressed and resolved on account of the detailed changes in the modelling.

No changes made in response to comment.

Comment: In addition, potassium (K) does not appear to have been treated as a major element in the melt modeling, where it is instead considered only as a trace element, whereas K is treated as a major element in the Theriak-Domino

calculations. Input data and element classifications should be consistent across all modeling approaches, and any deviations should be clearly justified since it adds more uncertainty.

Response: This comment has been addressed and resolved on account of the detailed changes in the modelling.

No changes made in response to comment.

Comment: Furthermore, the manuscript does not explain why a single mantle composition from Palme and O'Neill was used in combination with major-element compositions from Walter (2013). As I understand it, the latter represents a modeled composition resulting from melt extraction, rather than a primitive mantle composition.

Response: We refer the reviewer to Walter (2013). Admittedly, this isn't spelled out very well in Table 1 of Walter (2013), but in that text, it is explicitly stated that the major element composition that we have employed is a primitive mantle composition *inferred backwards from melt-extraction trends in peridotites*, rather than itself being the product of melt extraction. Therefore, it is perfectly reasonable to use this as the major element composition of a primitive mantle source for modelling in conjunction with the trace element compositions of primitive mantle from Palme and O'Neill (2013).

No changes made in response to comment.

Consequently, the modeling workflow appears to be based on a model-derived starting composition rather than a commonly accepted mantle reference composition. This choice requires clear justification, as it directly affects the validity and interpretation of the results.
In summary, I recommend the following revisions to improve the robustness of the manuscript:
1) Petrography

Comment: It is essential to include petrographic documentation, with representative thin-section photomicrographs, to demonstrate that the studied rocks are indeed pristine and preserve primary igneous mineral assemblages and textures.

Response: See comment above.

No changes made in response to comment.

Comment: 2) Methodological consistency:
I strongly recommend adopting a more methodologically consistent modeling approach. At least four different software packages, each based on different calculation methods and assumptions, are currently used. Where possible, calculations should be carried out within a single thermodynamic framework rather than being distributed among multiple empirical and thermodynamic approaches. This would reduce uncertainty propagation and significantly improve internal consistency, reproducibility, and clarity.
For example, the calculations presented in Sections 1.2–1.6 could be revisited and performed using MELTS-based software, as done in later sections. This would also allow direct comparison of thermodynamically modeled trace-element compositions with measured rock compositions.

Response: We have switched our thermodynamic modelling in its entirety to MAGEMin (Riel *et al.*, 2022). This should address the reviewer's concern about consistency, as MAGEMin is a much newer software than either alphaMELTS/pMELTS or Theriak-Domino and allows us to take the same methodological approaches that required the use of two software in the previous manuscript version. This software is, in our opinion, on the cutting edge of thermodynamic modelling. Furthermore, the x-eos and a-x models that we use are considered to produce much more accurate results when applied to mantle melting than pMELTS (Otto *et al.*, 2023). We have also discussed all errors associated with modelling in the *Supplementary Methods*.

Changes made in response to comment:
Modelling framework revised: the modelling now only uses MAGEMin, addressing Reviewer 4's concerns about consistency.
Supplementary Information, lines 494-548: Added section "Modelling uncertainties," which addresses Reviewer 4's concerns.

Comment: 3) Theriak-Domino modeling and database transparency:
I understand that Theriak-Domino was used for calculations in pressure ranges where the parameters in alphaMELTS are considered less reliable (i.e., at elevated pressures). One way to address this issue would be to run both approaches and compare the results. If the results are similar, pMELTS outputs could then be used to assess trace-element behavior.
If the authors decide to retain Theriak-Domino melting calculations, it is necessary to re-run the models using the corrected thermodynamic database, given the

documented errors in the previously used version, and to explicitly discuss the limitations of applying this model to hydrous peridotitic systems. In addition, I suggest making the thermodynamic database available and clearly indicating which phases were activated or suppressed.

Response: See comments above. The thermodynamic database is already available as part of the MAGEMin software, and the phases activated are clearly stated in the *Methods* and *Supplementary Methods*.

Changes made in response to comment:

Modelling framework revised: the modelling now only uses MAGEMin, addressing Reviewer 4's concerns about consistency.

Supplementary Information, lines 494-548: Added section "Modelling uncertainties," which addresses Reviewer 4's concerns.

References

Belgrano T. M., Diamond L. W., Vogt Y., Biedermann A. R., Gilgen S. A. and Al-Tobi K. (2019) A revised map of volcanic units in the Oman ophiolite: insights into the architecture of an oceanic proto-arc volcanic sequence. *Solid Earth* **10**, 1181–1217.

Bénard A., Arculus R. J., Nebel O., Ionov D. A. and McAlpine S. R. B. (2017) Silica-enriched mantle sources of subalkaline picrite-boninite-andesite island arc magmas. *Geochim. Cosmochim. Acta* **199**, 287–303.

Bénard A., Le Losq C., Nebel O. and Arculus R. J. (2018) Low-Ca boninite formation by second-stage melting of spinel harzburgite residues at mature subduction zones: new evidence from veined mantle xenoliths from the West Bismarck Arc. *Contrib. to Mineral. Petrol.* **173**, 1–25.

Brown M., Pearce J. A. and Johnson T. E. (2024) Is plate tectonics a post-Archean phenomenon? A petrological perspective. *J. Geol. Soc. London.* **181**.

Cooper L. B., Plank T., Arculus R. J., Hauri E. H., Hall P. S. and Parman S. W. (2010) High-Ca boninites from the active Tonga Arc. *J. Geophys. Res. Solid Earth* **115**.

Dilek Y. and Furnes H. (2014) Ophiolites and Their Origins. *Elements* **10**, 93–100.

Ding C.-L., Nemchin A., Johnson T., Norman M. D., Guan Y., Tian L.-L., Xie W.-L., Li L.-S., Zhou S.-D., Xu K.-X. and Wang X.-L. (2025) A potential mantle origin for precursor rocks of high-Mg impact glass beads in Chang'e-5 soil. *Sci. Adv.* **11**.

Golowin R., Portnyagin M., Hoernle K., Hauff F., Gurenko A., Garbe-Schönberg D., Werner R. and Turner S. (2017) Boninite-like intraplate magmas from Manihiki Plateau require ultra-depleted and enriched source components. *Nat. Commun.* **8**, 14322.

Green D. H., Hibberson W. O., Kovács I. and Rosenthal A. (2010) Water and its influence on the lithosphere–asthenosphere boundary. *Nature* **467**, 448–451.

Green D. H., Hibberson W. O., Rosenthal A., Kovács I., Yaxley G. M., Falloon T. and Brink F. (2014) Experimental study of the influence of water on melting and phase assemblages in the upper mantle. *J. Petrol.* **55**, 2067–2096.

Grove T. L., Chatterjee N., Parman S. W. and Médard E. (2006) The influence of H2O on mantle wedge melting. *Earth Planet. Sci. Lett.* **249**, 74–89.

Guilmette C., Smit M. A., van Hinsbergen D. J. J., Gürer D., Corfu F., Charette B., Maffione M., Rabeau O. and Savard D. (2018) Forced subduction initiation recorded in the sole and crust of the Semail Ophiolite of Oman. *Nat. Geosci.* **11**, 688–695.

Hernández-Montenegro J. D., Palin R. M., Zuluaga C. A. and Hernández-Uribe D. (2021) Archean continental crust formed by magma hybridization and voluminous partial melting. *Sci. Rep.* **11**, 1–9.

Hoare L., Rzehak L. J. A., Kommescher S., Jansen M., Rosing M. T., Nagel T., Millet M.-A., Hoffmann J. E. and Fonseca R. O. C. (2023) Titanium isotope constraints on the mafic sources and geodynamic origins of Archean crust. *Geochemical Perspect. Lett.* **28**, 37–42.

Hoffmann J. E. and Wilson A. H. (2017) The origin of highly radiogenic Hf isotope compositions in 3.33 Ga Commondale komatiite lavas (South Africa). *Chem. Geol.* **455**, 6–21.

Holland T. J. B. and Powell R. (2011) An improved and extended internally consistent thermodynamic dataset for phases of petrological interest, involving a new equation of state for solids. *J. Metamorph. Geol.* **29**, 333–383.

Holland T. J. B., Green E. C. R. and Powell R. (2018) Melting of Peridotites through to Granites: A Simple Thermodynamic Model in the System KNCFMASHTOCr. *J. Petrol.* **59**, 881–900.

Ishikawa Y., Sawaguchi T., Iwaya S. and Horiuchi M. (1976) Delineation of prospecting targets for Kuroko deposits based on modes of volcanism of underlying dacite and alteration halos. *Min. Geol*. **26**, 105–117

Ishizuka O., Tani K., Reagan M. K., Kanayama K., Umino S., Harigane Y., Sakamoto I., Miyajima Y., Yuasa M. and Dunkley D. J. (2011) The timescales of subduction initiation and subsequent evolution of an oceanic island arc. *Earth Planet. Sci. Lett.* **306**, 229–240.

Johnson T. E., Brown M., Gardiner N. J., Kirkland C. L. and Smithies R. H. (2017) Earth's first stable continents did not form by subduction. *Nature* **543**, 239–242.

König S., Münker C., Schuth S., Luguet A., Hoffmann J. E. and Kuduon J. (2010) Boninites as windows into trace element mobility in subduction zones. *Geochim. Cosmochim. Acta* **74**, 684–704.

Lamont T. N., Loader M. A., Roberts N. M. W., Cooper F. J., Wilkinson J. J., Bevan D., Gorecki A., Kemp A., Elliott T., Gardiner N. J. and Tapster S. (2024) Porphyry copper formation driven by water-fluxed crustal melting during flat-slab subduction. *Nat. Geosci.* **17**, 1306–1315.

Large R. R., Gemmell J. B., Paulick H. and Huston D. L. (2001) The Alteration Box Plot: A Simple Approach to Understanding the Relationship between Alteration Mineralogy and Lithogeochemistry Associated with Volcanic-Hosted Massive Sulfide Deposits. *Econ. Geol.* **96**, 957–971.

Lehmann W., Regelous M., Haase K., Keith M. and Woelki D. (2025) Geochemistry of the sheeted dyke complex, Troodos Ophiolite (Cyprus): Evidence for the magmatic evolution during subduction initiation. *Chem. Geol.* **681**, 122743.

Mathieu L. (2018) Quantifying Hydrothermal Alteration: A Review of Methods. *Geosciences* **8**, 245.

Moyen J. F. and van Hunen J. (2012) Short-term episodicity of Archaean plate tectonics. *Geology* **40**, 451–454.

Mullen E. K. and McCallum I. S. (2014) Origin of Basalts in a Hot Subduction Setting: Petrological and Geochemical Insights from Mt. Baker, Northern Cascade Arc. *J. Petrol.* **55**, 241–281.

O'Neill H. S. C. (2016) The Smoothness and Shapes of Chondrite-normalized Rare Earth Element Patterns in Basalts. *J. Petrol.* **57**, 1463–1508.

Otto T., Stevens G., Mayne M. J. and Moyen J. F. (2023) Phase equilibrium modelling of partial melting in the upper mantle: A comparison between different modelling methodologies and experimental results. *Lithos* **444**–**445**, 107111.

Palme H. and O'Neill H. S. C. (2013) Cosmochemical Estimates of Mantle Composition. In *Treatise on Geochemistry: Second Edition* Elsevier Ltd. pp. 1–39.

Pearce J. A. (2008) Geochemical fingerprinting of oceanic basalts with applications to ophiolite classification and the search for Archean oceanic crust. *Lithos* **100**, 14–48.

Pearce J. A. and Reagan M. K. (2019) Identification, classification, and interpretation of boninites from Anthropocene to Eoarchean using Si-Mg-Ti systematics. *Geosphere* **15**, 1008–1037.

Ribeiro J., MacLeod C. J. and Lissenberg C. J. (2023) Did the Troodos ophiolite of Cyprus form during subduction inception? *Chem. Geol.* **641**, 121774.

Riel N., Kaus B. J. P., Green E. C. R. and Berlie N. (2022) MAGEMin, an Efficient Gibbs Energy Minimizer: Application to Igneous Systems. *Geochemistry, Geophys. Geosystems* **23**, 1–27.

Rollinson H. (2022) No plate tectonics necessary to explain Eoarchean rocks at Isua (Greenland). *Geology* **50**, 147–151.

Schmidt M. E., Kizovski T. V., Liu Y., Hernandez-Montenegro J. D., Tice M. M., Treiman A. H., Hurowitz J. A., Klevang D. A., Knight A. L., Labrie J., Tosca N. J., VanBommel S. J., Benaroya S., Crumpler L. S., Horgan B. H. N., Morris R. V., Simon J. I., Udry A., Yanchilina A., Allwood A. C.,

Cable M. L., Christian J. R., Clark B. C., Flannery D. T., Heirwegh C. M., Henley T. L. J., Henneke J., Jones M. W. M., Orenstein B. J., Herd C. D. K., Randazzo N., Shuster D. and Wadhwa M. (2025) Diverse and highly differentiated lava suite in Jezero crater, Mars: Constraints on intracrustal magmatism revealed by Mars 2020 PIXL. *Sci. Adv.* **11**, 1–22.

Schmidt M. W. and Jagoutz O. (2017) The global systematics of primitive arc melts. *Geochemistry, Geophys. Geosystems* **18**, 2817–2854.

Sizova E., Gerya T., Stüwe K. and Brown M. (2015) Generation of felsic crust in the Archean: A geodynamic modeling perspective. *Precambrian Res.* **271**, 198–224.

Smith P. M. and Asimow P. D. (2005) Adiabat-1ph: A new public front-end to the MELTS, pMELTS, and pHMELTS models. Geochemistry, Geophys. Geosystems 6, 1–8.

Smithies R. H., Champion D. C. and Sun S.-S. (2004) The case for Archaean boninites. *Contrib. to Mineral. Petrol.* **147**, 705–721.

Smithies R. H., Champion D. C., Van Kranendonk M. J., Howard H. M. and Hickman A. H. (2005) Modern-style subduction processes in the Mesoarchaean: Geochemical evidence from the 3.12 Ga Whundo intra-oceanic arc. *Earth Planet. Sci. Lett.* **231**, 221–237.

Smithies R. H., Champion D. C., Van Kranendonk M. J. and Hickman A. H. (2007) *Geochemistry of volcanic units of the northern Pilbara Craton, Western Australia*.

Smithies R. H., Ivanic T. J., Lowrey J. R., Morris P. A., Barnes S. J., Wyche S. and Lu Y. J. (2018) Two distinct origins for Archean greenstone belts. *Earth Planet. Sci. Lett.* **487**, 106–116.

Smithies R. H., Lu Y., Kirkland C. L., Johnson T. E., Mole D. R., Champion D. C., Martin L., Jeon H., Wingate M. T. D. and Johnson S. P. (2021) Oxygen isotopes trace the origins of Earth’s earliest continental crust. *Nature* **592**, 70–75.

Soderman C. R., Weller O. M., Beard C. D., Riel N., Green E. C. R. and Holland T. J. B. (2025) A mid-crustal tipping point between silica-undersaturated and silica-oversaturated magmas. *Nat. Geosci.* **18**, 555–562.

Till C. B., Grove T. L. and Withers A. C. (2012) The beginnings of hydrous mantle wedge melting. *Contrib. to Mineral. Petrol.* **163**, 669–688.

Todd E., Gill J. B. and Pearce J. A. (2012) A variably enriched mantle wedge and contrasting melt types during arc stages following subduction initiation in Fiji and Tonga, southwest Pacific. *Earth Planet. Sci. Lett.* **335**–**336**, 180–194.

Turner S. J. and Langmuir C. H. (2022) An Evaluation of Five Models of Arc Volcanism. *J. Petrol.* **63**, 1–25.

van Hunen J. and van den Berg A. P. (2008) Plate tectonics on the early Earth: Limitations imposed by strength and buoyancy of subducted lithosphere. *Lithos* **103**, 217–235.

Walsh C., Kamber B. S. and Tomlinson E. L. (2023) Deep, ultra-hot-melting residues as cradles of mantle diamond. *Nature* **615**, 450–454.

Walter M. J. (2013) Melt Extraction and Compositional Variability in Mantle Lithosphere. In *Treatise on Geochemistry: Second Edition* Elsevier Ltd. pp. 393–419.

Wang X., Nebel O., Churchus A. and Cawood P. A. (2025) Tin isotope systematics in subduction zones. *Geochim. Cosmochim. Acta* **399**, 18–34.

Wanke M., Clynne M. A., von Quadt A., Vennemann T. W. and Bachmann O. (2019) Geochemical and petrological diversity of mafic magmas from Mount St. Helens. *Contrib. to Mineral. Petrol.* **174**, 0.

Responses to reviewer feedback on revised manuscript of
Modern arc-like water content in the source of 3.1-billion-year-old volcanic rocks

Eric D. Vandenburg *et al.*

We thank the reviewers for taking the time to review our revised manuscript and providing us with excellent feedback. We have revised the manuscript to reflect these suggestions. Below, we respond to the comments and edits suggested by the reviewers and outline how we have strengthened the manuscript in response to their feedback. Reviewer feedback is highlighted in cyan.

Responses to feedback from Reviewer #3

This was the second time I'm reading this manuscript, and I believe the revision has been going on for longer. I appreciate the authors reading and replying to all my comments and suggestions. The manuscript has now all calculations on the same software making it more consistent. I'm not familiar with the software that was used but I was able to follow the steps using the supplementary files. A good number of my suggestions did not make into the manuscript, but I understand that's the authors choice.

Comment: However, my main concern is still about the LILE and sample preservation. The authors and I have opposing views here since a good number of geochemical plots can have varying interpretations. For example, figure R2 displays the lack of covariation between Rb, Cs, Ba and MgO of the Whundo calc-alkaline basalts and modern calc-alkaline basalt. There is no statistical trend on this figure given the low R2 values, and assuming a similarity is simply a false premise. Instead of replying and debating the interpretation of each plot, I'd rather raise the point that geochemistry is extremely powerful tool, but there are limitations on what it can do alone. Hence the need to include the petrography. Exactly the qualitative aspect showing that the samples contain chlorite and sericite, needs to be on the main text.

Figure R5 is ready and could be added to the supplements. With this information, the reader can approach the manuscript and be clear what is the alteration. Whether it is an isochemical system will be up to reader to make up their mind.

In my opinion, the rocks are relevant enough to be worth publishing. The main conclusions are highly debatable but at this point this discussion should be made public after minor revisions just to include the petrography text and supplementary figure.

Response: We are grateful for the concern but disagree with the reviewer that the comparison between Whundo calc-alkaline basalts and modern central Aleutian arc calc-alkaline basalts in Figure R2 in the previous response document is a false premise, considering that the purpose of the diagram was to demonstrate that modern arc lavas do not show correlations in Rb, Cs and Ba vs MgO space if they are from disparate eruptive centres. Given the spatiotemporal extent of the Whundo Group (~10 km-thick and 114 km^2 over 20-30 Myr), it would be an equally false premise to ascribe the lack of correlation entirely to alteration, especially considering the metamorphic grade is relatively low.

However, we agree with the reviewer that a petrographic figure should be included in the Supplementary Information, given that Smithies et al. (2005) and subsequent studies did not include images of the rocks themselves. Therefore, we have added a modified version of Figure R5 to the Supplementary Information as a new Supplementary Figure 3. We thank the reviewer for the recommendation.

Likewise, we have added a brief description of the petrography to the main text, noting that there are minor degrees of chloritization and sericitization evident in the samples.

Changes made in response to comment:

Main Text, lines 112-124: added paragraph **"**The low metamorphic grade of the Whundo Group is such that the rocks have largely escaped the high degrees of alteration that are typical of rocks older than 3 Ga and fully retain their igneous textures. Many of the basalts are comprised of acicular pyroxenes set in a fine-grained to glassy groundmass, which has been devitrified (Supplementary Fig. 3). Alteration has been largely restricted to locally sericite-dusted plagioclase and minor to moderate degrees of chloritization affecting olivine and (partially) orthopyroxene; clinopyroxene is fully preserved in most samples, which is uncommon for 3.1 Ga rocks. In line with previous findings[26], alteration screening (Supplementary Methods, Supplementary Fig. 2) suggests that the low degrees of alteration have not substantially affected the large-ion lithophile element (LILE) concentrations of the Whundo lavas, which have values that overlap with modern arc lavas. While there is some compositional scatter, this is expected given the thickness (~10 km), extent (114 km^2) and age range (20-30 Myr) of the studied package; such scatter is also observed in modern arcs[28]. We refer the reader to the Supplementary Methods for further discussion on alteration."

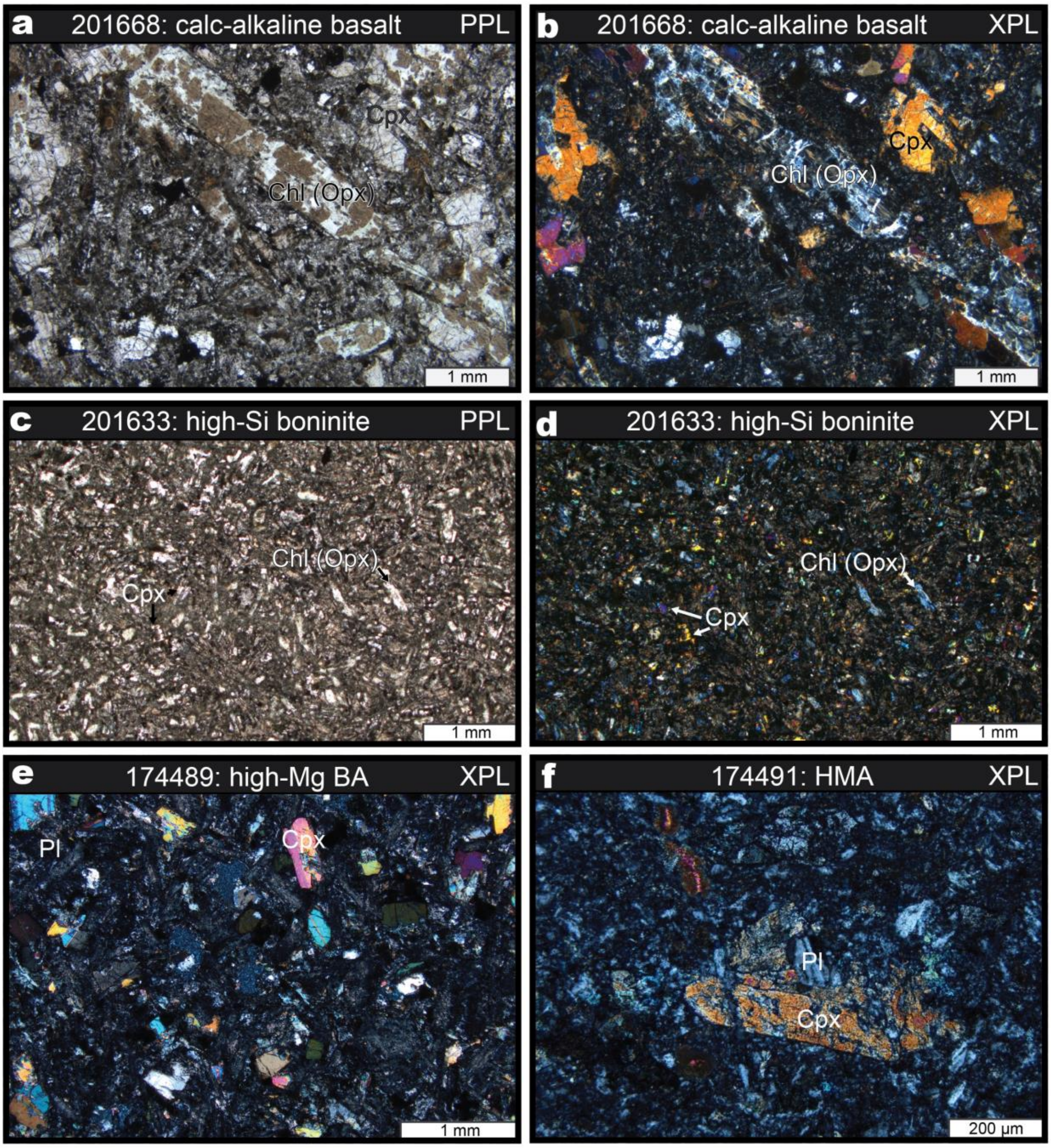

NEW: **Supplementary Figure 3.**

References

Smithies R. H., Champion D. C., Van Kranendonk M. J., Howard H. M. and Hickman A. H. (2005) Modern-style subduction processes in the Mesoarchaean: Geochemical evidence from the 3.12 Ga Whundo intra-oceanic arc. *Earth Planet. Sci. Lett.* **231**, 221–237.

General comments on Vandenburg et al.

"Modern arc-like water content in the source of 3.1-billion-year-old volcanic rocks" or "Earth's first primitive subduction occurred in the missing 2 majority of Archean crust"

This is the first time I'm reading this manuscript, but I understand it has already been through some rounds of revision. I suspect some of my comments might have been previously raised, but that happens when joining an on-going revision. The manuscript is polished and well-written presenting a series of samples with boninite signature, which is rightfully uncommon during the Archean and only known on a handful of locations. The identification and description of those rocks alone would be worth of publication – even considering the boninite like signature of the Whundo group was already mentioned by Smithies et al. (2005); However, the authors focused on an elaborate petrological model instead. My major concern is the very basic assumption that the rocks are pristine and represent lavas, with REE, HFSE and LILE elements preserved. At the current state of the manuscript, I'm not convinced that is the case.

The REE and HFSE could likely represent the original signature, but expanding that to the LILE (K, Rb, Ba, Pb, Ce etc.) is much more difficult. I'll use as an example the work by Shervais et al. (2021), reference 74. of the manuscript. Their work drilled 800 meters into the boninite type locality, and show that K, Rb, Ce are significantly altered with minor mobility on Pb, Ba, Sr. The lack of correlation with MgO is used as argument for mobility, and further conclusion about those elements are very limited. The lack of correlation with Rb presented on figure sup 2 c and b of the manuscript is similar to the lack of correlation found on boninites, but that doesn't make the sample boninites nor show that their LILE are preserved. I find it incredibly difficult to understand that, for an Archean boninite that has been tectonized to at least some extent, that those elements are preserved. Even at low temperature sea-floor alteration, low-temperature metamorphism, or subaerial exposure those elements are known to be mobilized. Therefore, a major aspect of the conclusions should be reconsidered, and I believe the data is overinterpreted in those terms. Additionally, there are signs of contamination that needs to be addressed.

The authors put a lot of effort to explain the models, which I appreciate. I think the calculations itself are reasonable, and only some minor changes would be needed. My main question is why the mixing and fractional crystallization (section 1.6) wasn't done using alphamelts, which technically is very similar to an AFC model. That would provide an entirely consistent thermodynamic model. I've also added a collaborator as a co-reviewer who's very qualified in thermodynamic modelling who's providing a co-revision on the calculations.

My main suggestion for the manuscript is focusing on presenting a well-characterized description of the samples with petrography and outcrop photos – essentially circling back to some images that seem to be progressively vanishing with each revision. Archean boninites are rare and on it is own represent a significant finding and should be very carefully presented before elaborating on geochemical models. I'd suggest a major revision where all the considerations about the LILE are readdressed. Implications regarding the dripduction and REE and maybe HFSE are fine.

Specific points:

Line 43 – **"These samples provide a rare Archean analogue of modern arc volcanism, with fluid-mobile elements consistent with primary magmatic values."** - I'd remove this

sentence. It is a very bold statement for altered Archean rocks. Maybe rephrase it like : "…provide rare Archean analogue for crustal recycling and mantle refertilization."

Line 58 - **"If widespread, such regional proto-subductions likely promoted volatile exchange between mantle and ocean-atmosphere reservoirs during the Archean."** – If it was widespread there'd be more boninites across the Archean localities. AS made clear on the manuscript, it is not the case. I'd simply remove the sentence.

LOI values on table 667710_0_data_set_11698630_t4trxx - those are high, if those are pristine lavas which hydrated phase would hold this much water? Amphiboles would take up to 2 wt.% and micas about 4-5 wt.% being generous. I'm suspicious the 5% LOI is okay for some modern lavas where the formation environment is clear, but I don't think it's the case for your samples.

Fig. 2b - The vertical trend on the plot could indicate a few things. An AFC trend would have a similar shape with end-member on each end of the curve. With your mixing model, I'd expect to see the calc-alkaline and the boninites on the same trend and one of the end-points with the calc-alkaline composition. I'm don't think the mixing is clear from this plot, so maybe the vertical spread could have other causes such as the ones argued in Pearce (2008).

Line 211 **– "Unlike many Archean basalts, evidence for crustal contamination by evolved proto cratonic basement in the Whundo Group lavas is scarce."** – See my comment below on line 393, but I think your mixing model work just as well with assimilation giving there is no difference between the solid and liquid composition of the calc-alkaline basalts. The relative timing of your model with calc-alkaline forming first also opens this possibility.

Line 216 – **"Furthermore, the similarity between the Th/Yb-Nb/Yb systematics of the primitive and felsic26 lavas (Fig. 2b) suggests that they were not contaminated by ancient crust and therefore, erupted off-plateau."** – See the previous comment about figure 2b. There is a vertical array on your plot, and you should expand on that.

Line 218 – **"While some degree of contamination by coeval felsic units cannot be ruled out, the lack of covariation of contamination-sensitive proxies with increasing differentiation indicates that contamination was not a major contributor to the incompatible element budgets of the primitive lavas (Supplementary Fig. 6)."** – Your plots on Fig. sup. 6 show a shift towards the Whundo felsic lavas following your "calc-alkaline trend" purple arrow, which is exactly how a contamination would look like. Inclusive, some of the calc-alkaline are within the felsic field. Maybe there's another trend on your plot? The differentiation trends using ratios on your axis should have a curved shaped, not linear.

Line 229 – **"The role of fluids/melts in the melting process can be studied by comparing petrogenetic trace element ratios that track fluid enrichment (e.g., Pb/Ce, Cs/La, Ba/Th) to those that monitor the degree of melting (e.g., Sm/Lu, Gd/Ho)."** – Some of those elements are unlikely to be preserved, but you don't need them to suggest flux melting. Those ratios might as well reflect some post emplacement alteration that you simply can't tell. Even for modern lavas it is difficult dealing with those elements. However, just by having a boninite u-shaped REE, the mantle source needs to be previously depleted and followed by refertilization. How the refertilization happens is up for debate, where you suggested dripduction and that's reasonable.

Fig 5 and Line 234 – "Negative covariations of Pb/Ce vs. Sm/Lu and Ba/Th and Cs/La vs. Gd/Ho indicate that the calc-alkaline magmas were produced by fluxed melting" – same lines as the previous comment. The U-shaped REE is a very strong petrological indicator, together with some other HFSE. The manuscript would be much better if the arguments were built around those elements instead of LILE. There's no need to use Pb/Ce or Ba/Th ratios to claim flux melting, given that calc-alkaline rocks, by definition, have a late Al enrichment caused by the depolymerization of a fluxed magma.

Line 263 – "First, we corrected representative samples from each group for fractional crystallization back to equilibrium with mantle olivine, which enabled us to calculate the trace element compositions of theparental melts." – Include the composition(s) of the olivine back-calculated to (Fo86 and 90 I suppose).

Line 268 – "HREE budgets are fluid-immobile and unaffected by subduction enrichment, so we matched the calculated HREE abundances of the modified sources with those produced by anhydrous partial melting of a primitive mantle source." – This sentence is a bit confusing; would you mind rephrasing? I understood that you match the HREE of the modelled mantles, is that right?

Line 271 – "This allowed us to estimate the amount of mantle wedge depletion before dripduction and characterize the contribution of the subduction component to mantle budgets of incompatible trace elements." – Which incompatible elements? The calculation for the REE and some HFSE should be okay. I'm curious about the U and Th given the radiogenic nature, would the decay of 238U and 235U influence the concentration given the 3.1 Ga age of the samples and the sub ppm concentration measured?

Line 276; line 965 ¶ on how the mass balance was done; – In simple terms the calculation is the [x]initial mantle – [x]modified mantle = [x]change; where the change is presented as % and [x] is the concentration of each element. I suggest having an equation on the explanation to be clearer given thar some equations are already used on the supplements.

Line 388- "… surrounding mantle (Fig. 6ci), providing up to 98% of the element budget of the…" I just realized that U is up to 98%. See my comment about line 271 above!

Line 393 – "Calc-alkaline basalts form when the arc melting regime comprises relatively undepleted lherzolite (Fig. 6ci). Later, when residual harzburgites enter the dripduction arc melting regime, fluxed melting produces boninites exhibiting the "ghost garnet" memory of the source's prior depletion history (Fig. 6cii)." – So, the boninites would form after the calc-alkaline. That is the opposite of what normally occurs in modern settings, where boninites are the very first to form in most cases. The model needs the depletion of a lherzolite, and refertilize the depleted mantle, now a harzburgite, to produce boninites with some of the boninites mixing with the calc-alkaline. A few points regarding the model:

1 – the timing difference comparing to modern subduction is interesting, I'd highlight that and expand on it.

2 – I'll point out (again) that contamination needs to be addressed. Mixing magmas, where both are liquids on the source, and contamination with a liquid and a solid on the crust, can be difficult to tell a part purely by WR major and trace element geochemistry. If the calc-alkaline composition can be used to mix and obtain some of the boninites, for me that also suggest that some of your boninites are contaminated. Especially given the proposed model where the green intrudes the gray on panel 6 c(ii). Bottom line, the mixing between the calc-

alkaline and boninites could also be seem as contamination, and some of your calc-alkaline are show strong signs of contamination by the Whundo felsic.

3 – The mixing calculations used average compositions. I'd suggest also using the extreme compositions (highest and lowest MgO, SiO2 or whichever differentiation parameter you choose). That might be more realistic instead of an average in terms of significance. If you decide to use the average, it's important to tell the number of samples used to calculate it and the 2 standard deviation.

References

J.A. (2008) Geochemical fingerprinting of oceanic basalts with applications to ophiolite classification and the search for Archean oceanic crust. *Lithos* 100, 14–48, https://doi.org/10.1016/j.lithos.2007.06.016.
